# The cost-effectiveness of interventions used for the screening, diagnosis and management of anaemia in pregnancy: A systematic review

**DOI:** 10.1371/journal.pgph.0004392

**Published:** 2025-04-24

**Authors:** Connor Luke Allen, Katherine Eddy, Joshua F. Ginnane, Sarah Cheang, Renae Nguyen, Nick Scott, Joshua P. Vogel, Annie McDougall

**Affiliations:** 1 School of Public Health and Preventive Medicine, Monash University, Melbourne, Australia; 2 Women’s, Children’s and Adolescent’s Health Program, Burnet Institute, Melbourne, Australia; 3 Monash Institute of Pharmaceutical Sciences, Monash University, Melbourne, Australia; University College London, UNITED KINGDOM OF GREAT BRITAIN AND NORTHERN IRELAND

## Abstract

An estimated 40% of pregnant women worldwide are anaemic, of which 80% live in low- and middle-income countries (LMICs). The reality of finite health budgets, particularly in low-resource settings, means that interventions used for screening, diagnosing, and treating anaemia in pregnancy need to be informed by cost-effectiveness evidence. We conducted a systematic review to identify all studies evaluating the cost-effectiveness of managing anaemia in pregnancy. We searched two health economics (NHS EED and EconLit) and four medical (MEDLINE, Embase, CINAHL Plus and CENTRAL) databases for relevant studies published up to the 9^th^ of August 2024. Studies were eligible if they conducted an economic evaluation of any intervention used in the management of anaemia in pregnancy regardless of aetiology, provided that anaemia was a specified outcome. Data were extracted and study quality assessed by two independent reviewers using the extended CHEC-E tool. Due to significant heterogeneity, data were analysed narratively. 19 eligible cost-effectiveness studies were identified. Nine studies related to iron deficiency anaemia, finding that intravenous rather than oral iron supplements were cost-effective in most instances. Multiple micronutrient supplements were also found to be cost-effective compared to iron and folic acid supplements. Ten studies related to malaria-related anaemia, identifying several cost-effective antimalarial regimens; both preventative and therapeutic. Cost-effective delivery channels of antimalarials as well as non-pharmacological interventions were also identified. This review identifies several avenues through which the management of anaemia in pregnancy can be optimised from an economic perspective. Despite this, there is a significant deficit of cost-effectiveness evidence relating to this condition, which limited the deduction of cost-effectiveness for many of the interventions assessed.

## Introduction

Anaemia is a common haematological condition characterised by an inadequate number of red blood cells and/or inadequate haemoglobin concentration [1,2]. Anaemia in pregnancy represents an important public health concern for women worldwide. Data derived from 29 countries in the World Health Organization (WHO) Multicountry Survey found that severe anaemia during pregnancy or postpartum doubles the risk of maternal death [3]. Increased rates of maternal mortality are the direct result of a higher incidence of adverse outcomes such as stillbirth, preterm birth and intrauterine growth restriction [3,4]. An estimated 36% of pregnant women suffer from anaemia worldwide, however this varies significantly by region. West and central Africa and South Asia, for example, are reported as having the highest prevalences of anaemia among pregnant women, at 52% and 48% respectively [5]. Findings from the Global Burden of Disease Study covering 204 countries in 2021 demonstrated an inverse relationship between anaemia and socioeconomic development [6]. This is reflected in the skewed geographical distribution of anaemia during pregnancy and concentration of cases in low- and middle-income countries (LMICs) [5,7,8].

Anaemia can be caused by several factors, such as malnutrition or infectious or chronic diseases, many of which are more common in LMICs. Iron deficiency is the dominant cause of anaemia in pregnancy worldwide, causing an estimated 50% of cases [9]. Parasites are another leading cause of anaemia in pregnancy. Malaria in particular is an important parasitic cause of haemolytic anaemia throughout sub-Saharan Africa and South-East Asia, where more than 100 million women are estimated to be at risk of infection during pregnancy [10]. Soil-transmitted helminths, such as hookworm, are a parasitic aetiology which precipitate anaemia by extracting blood through the host’s intestinal wall. Parasites such as these are common in settings where poverty and poor sanitation are abundant [11,12]. Blood loss due to antepartum or threatened miscarriages can also lead to haemorrhagic anaemia during pregnancy. The likelihood of these adverse outcomes are significantly higher in low resource settings where effective preventive measures or treatments for maternal haemorrhage may be limited or unavailable [13]. Other causes of anaemia in pregnancy include haemolytic anaemias, which are predominantly the result of inherited conditions that induce haemolysis such as sickle cell disease and thalassaemias [14].

Economic evaluations compare alternative courses of action in relation to their costs and outcomes [15]. Cost-effectiveness analyses are one type of economic evaluation that compare the costs of two alternative interventions relative to health outcomes, e.g., cost per life saved [16]. The incremental cost of gaining a single health unit associated with choosing one intervention instead of another is represented by an Incremental Cost-Effectiveness Ratio (ICER). The amount of money deemed acceptable to spend per health unit gained is represented by a cost-effectiveness threshold [17]. By comparing ICERs against cost-effectiveness thresholds – set by individual studies or as reported elsewhere in the literature – determinations of interventions’ cost-effectiveness can be made. In instances where the cost per unit health gained is calculated for a single intervention, i.e., not in relation to a comparator, an Average Cost-Effectiveness Ratio (ACER) can be constructed [18]. Cost-effectiveness evidence can help identify efficient ways of allocating finite resources in health settings by identifying effective and inexpensive interventions [16]. Cost-effectiveness analyses, therefore, have the capacity to empower stakeholders by ensuring that both clinical outcomes and economic feasibility are enshrined in decision making processes. In low-resource settings, cost-effectiveness evidence becomes even more relevant in identifying whether clinical recommendations are feasible for implementation from an economic perspective. Given the concentration of anaemia in pregnant women in LMICs, the cost-effectiveness of interventions used in its management must be considered [7,8]. To date, there has been no synthesis of cost-effectiveness evidence of interventions used for the management of anaemia in pregnancy. This review aims to collate, appraise and interpret evidence from economic evaluations of interventions used in the screening, diagnosis, and management of anaemia in pregnancy.

## Materials and methods

This review followed the Preferred Reporting Items for Systematic Reviews and Meta-Analyses (PRISMA) statement (S1 Appendix) [19]. The protocol was registered prospectively on the PROSPERO (ID: CRD42022330169) on the 4^th^ of May 2022.

### Search strategy and selection criteria

The search strategy was designed with the help of an expert librarian and consisted of three broad domains: ‘economics’, ‘anaemia’, and ‘pregnancy’. The search was executed across two health economics (NHS EED and EconLit) and four medical (MEDLINE, Embase, CINAHL Plus and CENTRAL) databases on the 9^th^ of August 2024 (S2 Appendix).

Studies that performed a full economic evaluation of interventions used for detecting, diagnosing and managing anaemia in pregnancy were included. Studies pertaining to any aetiology of anaemia during pregnancy were eligible, i.e., haemoglobinopathies, iron deficiency, folate and vitamin B12 deficiency, malaria, hookworm, bone marrow disorders, chronic infections, autoimmune haemolytic anaemia and antepartum haemorrhage. This review only included studies that overtly specified anaemia as an outcome. Studies that utilised composite measures such as disability- and quality-adjusted life years (DALYs/QALYs) to determine an intervention’s cost-effectiveness must have specified that anaemia was included in this composite to be eligible. Interventions used at any stage of the continuum of care including prevention, screening, diagnosis, and management were eligible. Studies from all languages, settings and dates were eligible. Studies were excluded if they conducted assessments of an intervention in the absence of a comparator (i.e., partial economic evaluations). Studies that did not include pregnant women or did not report subset outcomes for pregnant women in the study population were excluded. Review articles and abstracts were not eligible.

### Data analysis

Search results were imported to Endnote v20.2 (https://endnote.com) and subsequently uploaded to Covidence (https://www.covidence.org). Two authors (CLA, RN, SC, AMcD) independently reviewed the identified studies, initially by title and abstract and subsequently by examination of full texts. Two authors (CLA, RN, SC, AMcD) then independently extracted key characteristics and outcome data as recommended by Wijnen et al., (2016) using Excel (S3 Appendix) [20]. The data that best answered our research question(s) are presented within the body of this manuscript, the complete data set has been included as S4 Appendix. Any discrepancies at screening or extraction stage were discussed in consultation with a third reviewer (AMcD, KE). Studies that were excluded are listed in S5 Appendix.

Cost effectiveness ratios were first extracted in the year and currency described in each study. These were subsequently converted to 2024 United States Dollars (USD) using a tool developed by the Campbell and Cochrane Economics Methods Group (CCEMG) and the Evidence for Policy and Practice Information and Coordinating Centre (EPPI-Centre) [21]. The CCEMG-EPPI Centre Cost Converter used a Gross Domestic Product (GDP) deflator index to create price-year adjusted cost estimates. These adjusted cost effectiveness ratios were then converted from the original currency to a target currency, in our review USD, using conversion rates based on Purchasing Power Parities (PPP) for GDP. The final values are appropriately adjusted for differences in price levels between countries, facilitating a comparison of costs from different settings and years. Where studies did not specify the year for which costs were obtained, the first year of data collection was used. Where costs were obtained across multiple years, the earliest was chosen. Prices described in the results section are the converted prices.

Study quality was assessed using an extended version of the Consensus Health Economic Criteria (CHEC-E) tool (S6 Appendix) [22,23]. Studies were evaluated against several criteria encompassing methodology, bias and comprehensiveness for which a score of up to 20 could be awarded. Where certain CHEC-E criteria were not applicable to a study, those domains were marked as “not applicable” and the study was scored out of the number of criteria that were applicable. Based on this evaluation, studies were reported as high (>75%), moderate (50%-74%), or low (<50%) quality (S7 Appendix). Two reviewers (CLA, KE) independently conducted quality assessment of the included papers using this approach with disagreements resolved through discussion. The heterogenous nature of the studies included favoured descriptive analysis of study findings rather than meta-analysis.

Cost-effectiveness calculations are highly dependent on effectiveness estimates used by individual studies, therefore, ensuring the accuracy of these estimates is vital in ensuring cost-effectiveness calculations are representative of most recent evidence [24]. To achieve this, we extracted effectiveness data described in each individual study and compared it to up-to-date effectiveness estimates from recent systematic and Cochrane reviews (S8 Appendix). When a study calculated cost-effectiveness using effectiveness estimates that differed significantly (outside of 95% confidence intervals) to those found in the literature, we considered this in the interpretation of their findings.

## Results

### Characteristics of included studies

**Fig 1:**
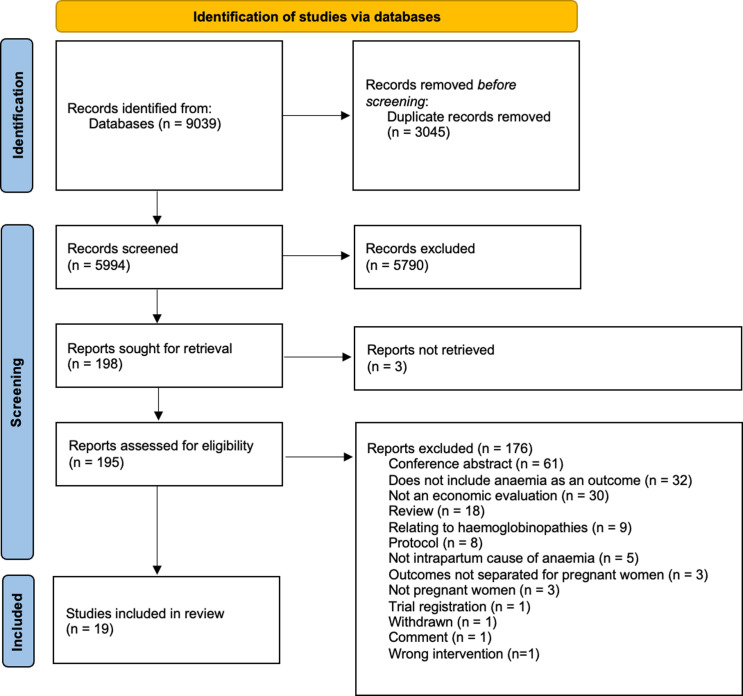
PRISMA Flowchart.

The search yielded 9,038 results, of which 19 studies [25–43] were eligible for inclusion (Fig 1). Of the included studies, nine [25,26,29,34,35,37,39,40,43] assessed interventions for use in iron-deficiency anaemia (Tables 1 and 2), and ten [27,28,30–33,36,38,41,42] for use in malaria-related anaemia (Tables 3 and 4). No cost-effectiveness studies were identified for other aetiologies of anaemia during pregnancy including folate and B12 deficiency, haemoglobinopathies, hookworm, antepartum haemorrhage and bone marrow disorders. Quality assessment identified 15 [26–28,30–34,36,38–43] high quality, one [35] moderate quality and three [25,29,37] low quality studies. Studies most frequently lost points in quality assessment for failing to state ethical issues, time horizon and study perspective. Included studies were published between 2004 and 2024. Studies were predominantly conducted in LMICs; two [33,36] were conducted in low-income countries, seven [29,34,35,37–40] in lower middle-income countries, one [25] in a high-income country. Seven studies [27,28,30–32,42,43] were conducted across multiple income levels; these included five [27,28,30–32] in both low- and lower middle- income countries, and two [42,43] in low-, lower middle- and upper middle-income countries. Two studies [26,41] did not report specific countries and therefore income levels were not determined. In four studies [28,30,37,40], effectiveness estimates for at least one outcome used to determine cost-effectiveness were significantly different to that described in the literature. These have been reported alongside cost-effectiveness findings below and in S8 Appendix.

**Table 1:** Characteristics of studies pertaining to iron deficiency related anaemia.

Study	Country	World Bank Income Level	Care setting	Interventions	Aim	Design/ analytic approach	Year of cost estimates	Cost-effectiveness ratio	Analytic viewpoint (perspective)	Time horizon (for effects)	CHEC overall quality assessment
Intravenous versus oral iron therapies											
Aftab et al., 2021 [25]	United Arab Emirates	High	Inpatient facility	Oral iron vs intravenous iron	To evaluate maternal-fetal outcomes and the cost-effectiveness of using iron supplementation during pregnancy.	Cost-effectiveness analysis based on a non-randomised quasi-experimental study	Unspecified	ICER per rise to desired Hb	Unspecified	Throughout the antenatal supplementation period to one week postpartum	6/18 (low quality)
Murugesan et al., 2023 [37]	India	Lower middle	Unspecified	Oral iron vs intravenous iron	To assess the mean change in the haemoglobin levels from baseline up to 60th day of treatment with different iron supplements and to assess cost-effectiveness.	Cost-effectiveness analysis based on a randomised control trial	2019-2020	ACER per increase in Hb%.	Unspecified	60 days	7/18 (low quality)
Ray et al., 2020 [39]	India	Lower middle	Inpatient facility	Oral iron vs intravenous iron	To conduct a cost-effectiveness analysis of intravenous iron sucrose over oral therapy for treatment of severe anaemia in pregnancy, alongside the randomised control trial, to inform policy.	Cost-effectiveness analysis based on a randomised control trial	2018	ICER per safe delivery	Limited societal perspective	Throughout the antenatal intervention period to six weeks postpartum	16/18 (high quality)
Saha et al., 2024 [40]	India	Lower middle	Primary health centre	Oral iron vs intravenous iron	To compare the cost- effectiveness of the intravenous iron sucrose with oral iron therapy among pregnant women with IDA.	Cost-effectiveness analysis based on a prospective study	Unspecified	ICER per QALY gained	Societal perspective	One year	16/20 (high quality)
No intervention versus iron fortification versus iron supplementation											
Baltussen et al., 2004 [26]	Various^*^	Various	Various	Varying levels of iron fortification vs varying levels of iron supplementation	To estimate the cost-effectiveness of iron supplementation and fortification in four regions of the world.	Cost-effectiveness based on a simulated population model	2000	ICER per DALY averted	Unspecified	Antenatal period	15/20 (high quality)
Multiple micronutrient supplementation versus iron and folic acid supplementation											
Kashi et al., 2019 [34]	Pakistan, India and Bangladesh	Lower middle	Outpatient setting	Iron and folic acid supplementation vs multiple micronutrient supplementation	To evaluate the cost-effectiveness of transitioning from iron and folic acid to multiple micronutrient supplementation.	Cost-effectiveness analysis based on meta-analyses and simulated model	2016	ICER per DALY averted	Unspecified	Antenatal and postpartum periods	19/20 (high quality)
Verney et al., 2023 [43]	Indonesia, Nigeria, Pakistan and Tanzania ^†^	Upper-middle, lower-middle and low-income	Unspecified	Iron and folic acid supplementation vs multiple micronutrient supplementation	To describe the underling methodology of the MMS Tool, present the results of a hypothetical MMS scale up scenario for four focus countries and 29 additional countries with pre‐loaded data, and discuss the application of the MMS Tool for supporting the translation of evidence into action.	Cost-effectiveness and cost-benefit analysis based on a modelling tool	2021	ICER per DALY averted and benefit-cost ratio	Unspecified	Antenatal and postpartum periods	18/20 (high quality)
Branded versus generic formulations of iron supplements											
Eeesha et al., 2022 [29]	India	Lower middle	Community and outpatient clinic	Generic Ferrous Ascorbate vs Branded Ferrous Ascorbate	To find out the least expensive alternative (of branded and generic drugs) for treating anaemic pregnant women.	Cost-effectiveness analysis based on a prospective randomised active control open label study	Unspecified	ACER per rise of 1 g/dL of Hb	Unspecified	Antenatal period (14–24 weeks gestation)	8/18 (low quality)
Training of health care workers to deliver education around iron supplementation versus no intervention											
Kurzawa et al., 2020 [35]	Bangladesh	Lower middle	Primary health centre	No intervention vs training of frontline healthcare workers	To investigate the cost-effectiveness of a program to train healthcare workers with the aim to increase supplementation consumption and adherence among pregnant women.	Cost-effectiveness analysis based on a quasi-experimental non-equivalent control group study	2018	ICER per DALY averted	Unspecified	Antenatal period	14/19 (moderate quality)

Abbreviations: ACER: average cost-effectiveness ratio, DALY: disability-adjusted life year, Hb: haemoglobin, ICER: incremental cost-effectiveness ratio, IDA: iron deficiency anaemia, IFA: iron and folic acid, IV: intravenous, MMS: multiple micronutrient supplementation, QALY: quality-adjusted life year.

**Table 2:** Cost-effectiveness findings of studies pertaining to iron deficiency anaemia.

Study	Comparator	Intervention	ICER per X	Cost-Effective (threshold)	Author conclusion(s)
Intravenous versus oral iron therapies					
Aftab et al., 2021 [25]	Oral iron supplements	IV iron supplements	US$108,633 unspecified year (US$138,580.53 2024) per rise to desired Hb As no year for costs was specified, the first year of data collection (2016) was used	No (US$75,000-US$100,000)	Oral iron supplementation was more cost-effective than intravenous to achieving a desired increase in Hb.
Ray et al., 2020 [39]	Oral iron therapy	IV iron sucrose	INR₹31,951 2018 (US$2143.75 2024) per safe delivery	Yes (Half GNI per capita)	IV iron supplementation was more cost-effective than oral iron supplementation for 67% of the models ran.
Saha et al., 2024 [40]	Oral iron therapy	IV iron sucrose	US$9.84 2020 (US$11.67 2024) per QALY gained As no year for costs was specified, the first year of data collection (2020) was used	Yes (India’s GDP per capita – unspecified figure)	IV iron sucrose was more efficacious and more cost-effective than oral iron therapy among pregnant women with moderate/severe anaemia.
Murugesan et al., 2023 [37]	–	Ferrous sulfate 200 mg twice daily for 60 days	INR₹675* 2019 (US$44.23 2024)	Not stated	All iron preparations were equally efficacious in improving Hb concentration. Cost per increase in Hb% was lowest in ferrous sulfate followed by intravenous iron sucrose.
		Ferrous ascorbate 200 mg twice daily for 60 days	INR₹1782.9* 2019 (US$116.81 2024)	Not stated	
		Ferrous fumarate 200 mg twice daily for 60 days	INR₹1110.7* 2019 (US$72.77 2024)	Not stated	
		IV iron sucrose 200 mg, based on iron requirement in divided doses and administered once in two weeks for a period of 60 days	INR₹786.7* 2019 (US$51.54 2024)	Not stated	
No intervention versus iron fortification versus iron supplementation					
Baltussen et al., 2004 [26]	Iron fortification 50% coverage	Iron fortification 80% coverage	African Subregion: dominated (iron fortification 80% coverage dominated by iron fortification 50% coverage) South American Subregion: dominated (iron fortification 80% coverage dominated by iron fortification 50% coverage) European Subregion: dominated (iron fortification 80% coverage dominated by iron fortification 50% coverage) Southeast Asian Subregion: Int$32 2000 (US$55.32 2024) per DALY averted	Yes (WHO < 3x GDP per capita recommendation) [44]	Iron fortification was the overall most-cost effective intervention; however, supplementation achieved greater health benefits and was still within the considered cost-effectiveness threshold.
	Iron fortification 80% coverage	Iron fortification 95% coverage	African Subregion: Int$20 2000 (US$34.58 2024) per DALY averted South American Subregion: Int$134 2000 (US$231.66 2024) per DALY averted European Subregion: Int$5,573 2000 (US$9,634.63 2024) per DALY averted Southeast Asian Subregion: Int$49 2000 (US$84.71 2024) per DALY averted	Yes (as above)	
	Iron fortification 95% coverage	Iron supplementation 50% coverage	African Subregion: Int$86 2000 (US$148.68 2024) per DALY averted South American Subregion: dominated (iron supplementation 50% coverage dominated by iron fortification 95% coverage) European Subregion: dominated (iron supplementation 50% coverage dominated by iron fortification 95% coverage) Southeast Asian Subregion: Int$32 2000 (US55.32 2024) per DALY averted	Yes (as above)	
	Iron supplementation 50% coverage	Iron supplementation 80% coverage	African Subregion: dominated (iron supplementation 80% coverage dominated by iron supplementation 50% coverage) South American Subregion: dominated (iron supplementation 80% coverage dominated by iron supplementation 50% coverage) European Subregion: dominated (iron supplementation 80% coverage dominated by iron supplementation 50% coverage) Southeast Asian Subregion: Int$179 2000 (US$309.46 2024) per DALY averted	Yes (as above)	
	Iron supplementation 80% coverage	Iron supplementation 95% coverage	African Subregion: Int$105 2000 (US$181.52 2024) per DALY averted South American Subregion: Int$1,349 2000 (US$2,332.16 2024) per DALY averted European Subregion: Int$35,762 2000 (US$61,825.52 2024) per DALY averted Southeast Asian Subregion: Int$182 2000 (US$314.64 2024) per DALY averted	Yes (as above)	
Multiple micronutrient supplementation versus iron and folic acid supplementation					
Kashi et al., 2019 [34]	Iron and folic acid supplementation	Multiple micronutrient supplementation	The Cochrane Scenario (MMS versus IFA or iron alone): -Pakistan: US$41.54 2016 (US$52.99 2024) per DALY averted -India: US$31.62 2016 (US$40.34 2024) per DALY averted -Bangladesh: US$21.26 2016 (US$27.12 2024) per DALY averted The Lancet Scenario (MMS versus IFA): -Pakistan: US$9.61 2016 (US$12.26 2024) per DALY averted -India: US$14.99 2016 (US$19.12 2024) per DALY averted -Bangladesh: US$10.74 2016 (US$13.70 2024) per DALY averted	Yes (WHO < 3x GDP per capita recommendation) [44]	MMS was more clinically efficacious and cost-effective than IFA.
Verney et al., 2023 [43]	Iron and folic acid supplementation	Multiple micronutrient supplementation	Baseline scenario -Indonesia: US$23.55 2021 (US$26.72 2024) per DALY averted -Nigeria: US$13.00 2021 (US$14.75 2024) per DALY averted -Pakistan: US$9.00 2021 (US$10.21 2024) per DALY averted -Tanzania: US$15.00 2021 (US$17.02 2024) per DALY averted Unit cost parity scenario -Indonesia: US$5.38 2021 (US$6.10 2024) per DALY averted -Nigeria: US$3.45 2021 (US$3.91 2024) per DALY averted -Pakistan: US$1.84 2021 (US$2.09 2024) per DALY averted -Tanzania: US$3.31 2021 (US$3.76 2024) per DALY averted	Yes (WHO < 3x GDP per capita recommendation) [44]	Transitioning from IFA to MMS was cost-effective across a range of settings and scenarios.
Branded versus generic formulations of iron supplements					
Eeesha et al., 2022 [29]	–	Generic Ferrous Ascorbate	INR₹269.38* unspecified year (US$20.38 2024) per increase in 1g/dL of Hb *As no year for costs was specified, the first year of data collection (2014) was used	No (author determination)	Branded Ferrous Ascorbate had better efficacy, a lower ACER and was associated with fewer adverse events compared to Generic Ferrous Ascorbate.
		Branded Ferrous Ascorbate	INR₹250.77* unspecified year (US$19.19 2024) per increase in 1g/dL of Hb *As no year for costs was specified, the first year of data collection (2014) was used	Yes (author determination)	
Training of health care workers to deliver education around iron supplementation versus no intervention					
Kurzawa et al., 2020 [35]	Standard care	Training healthcare workers	US$47.11 2018 (US$57.59 2024) per DALY averted	Yes (WHO < 3x GDP per capita recommendation) [44] Yes (Woods et al. supply side threshold for cost-effectiveness of $1400 USD) [45]	Capacity building of frontline health care workers was a cost-effective method of reducing anaemia among pregnant women.

*Cost-effectiveness ratios presented are ACERs

International dollars (Int) were represented as USD for the purposes of conversion

Abbreviations: ACER: average cost-effectiveness ratio, DALY: disability-adjusted life year, Hb: haemoglobin, GDP: Gross Domestic Product, ICER: incremental cost-effectiveness ratio, IFA: iron and folic acid, IV: intravenous, MMS: multiple micronutrient supplementation, QALY: quality-adjusted life year, WHO: World Health Organization.

**Table 3:** Characteristics of studies pertaining to malaria-related anaemia.

Study	Country	World Bank Income Level	Care setting	Interventions	Aim	Design/ analytic approach	Year of cost estimates	Cost-effectiveness ratio	Analytic viewpoint (perspective)	Time horizon (for effects)	CHEC overall quality assessment
Comparing different doses/coverage of IPTp-SP											
Choi et al., 2017 [27]	Ghana, Kenya, Malawi, Mozambique and Tanzania	Low and lower middle	Health facility (nurse administered)	Two doses of IPTp-SP Low coverage vs three doses of IPTp-SP Low coverage Two doses of IPTp-SP Low coverage vs three doses of IPTp-SP High coverage Two doses of IPTp Low coverage vs CTX	To compare the cost-effectiveness of CTX and IPTp-SP among HIV positive women.	Cost-effectiveness analysis of a microsimulation model	2015	ICER per DALY averted	Societal perspective	Antepartum and postpartum periods	17/20 (high quality)
Fernandes et al., 2015 [31]	sub-Saharan Africa (including Burkina Faso, Kenya, Malawi, Mali, Tanzania, and Zambia)	Low and lower middle	Health facility (nurse administered)	Two doses of IPTp-SP vs 3 or more doses of IPTp-SP	To project the cost-effectiveness of three or more doses of IPTp-SP versus two doses.	Cost-effectiveness based on a meta-analysis and cohort simulations	2012	ICER per DALY averted	Societal perspective	Antepartum and postpartum periods	19/20 (high quality)
Scott et al., 2020 [41]	Various*	Various	Unspecified	Baseline coverage of IPTp vs 95% coverage IPTp	To assess the cost-effectiveness of scaling up IPTp for anaemia reduction in pregnant women.	Cost-effectiveness analysis based on modelling	2017	ICER per case of anaemia averted	Unspecified	Unspecified	15/19 (high quality)
IPTp vs other interventions											
Fernandes et al., 2016 [30]	Burkina Faso, Ghana, Mali and The Gambia	Low and lower middle	Health facility (nurse administered)	IPTp-SP vs ISTp-AL	To estimate the cost-effectiveness of IPTp-SP and ISTp-AL.	Cost-effectiveness analysis based on a multi-centre, non-inferiority trial	2012	ICER per DALY averted	Health provider perspective	Antepartum and postpartum periods	20/20 (high quality)
Fernandes et al., 2020 [32]	Uganda and Kenya	Low and lower middle	Health facility (nurse administered)	Three doses of IPTp-SP vs three of IPTp-DP Monthly doses of IPTp-SP vs monthly doses of IPTp-DP	To determine the cost-effectiveness of IPTp-DP versus IPTp-SP to prevent clinical malaria and its sequelae in pregnancy.	Cost-effectiveness analysis based on three randomised control trials	2018	ICER per DALY averted	Health provider perspective	Antepartum and postpartum periods	19/20 (high quality)
Hansen et al., 2012 [33]	Uganda	Low	Various	Two doses of IPTp-SP vs insecticide treated bed nets (ITNs) Two doses of IPTp-SP vs ITNs and IPTp-SP	To determine the cost-effectiveness of IPTp, insecticide-treated bed nets and a combined intervention of both.	Cost-effectiveness analysis based on a randomised control trial	2004/2005	ICER per DALY averted	Health provider perspective	Antepartum and postpartum periods	17/19 (high quality)
Paintain et al., 2020 [38]	Indonesia	Lower middle	Health facility (nurse administered)	SST-DP vs monthly IPTp-DP	To determine the cost-effectiveness of administering SST-DP compared to IPTp-DP.	Cost-effectiveness analysis based on a randomised control trial	2016	ICER per DALY averted	Health provider perspective	Antepartum and postpartum periods	18/19 (high quality)
Sicuri et al., 2015 [42]	Benin, Gabon, Mozambique, Tanzania, Kenya	Low, lower middle and upper middle	Health facility (nurse administered)	Three doses of IPTp-SP vs two doses of IPTp-MQ (HIV negative) Daily CTX, ITNs and IPTp-placebo vs daily CTX, ITNs and three doses of IPTp-MQ (HIV positive)	To determine the cost-effectiveness of IPTp with a number of drugs.	Cost-effectiveness based on an open label randomised trial (HIV negative women) and a double-blind placebo-controlled trial (HIV positive)	2012	ICER per DALY averted	Health system perspective	Antenatal period to 6 weeks postpartum	17/19 (high quality)
Community vs health-centre delivery of IPTp											
Cirera et al., 2023 [28]	Madagascar, Mozambique, Nigeria and the Democratic Republic of the Congo (DRC)	Low and lower middle	Health centres and community settings	H-IPTp (health centre-based delivery of IPTp-SP at antenatal care [ANC] clinics) vs combined H-IPTp and C-IPTp (community-based delivery of IPTp-SP through community health workers [CHWs])	To assess the cost-effectiveness of C-IPTp in project intervention districts in addition to its delivery at the ANC clinics, compared with distributing IPTp at ANC clinics alone.	Cost-effectiveness based on a simulated population model	2018	ICER per DALY averted	Health provider perspective	Antepartum and postpartum periods	17/20 (high quality)
Mbonye et al., 2008 [36]	Uganda	Low	Hospital and community settings	Health centre-based delivery of IPTp-SP (H-IPTp) vs Community based delivery of IPTp-SP (C-IPTp)	To identify whether local health workers administer IPTp-SP to pregnant women cost-effectively.	Cost-effectiveness based on an interventional study	2003/2004	ICER per DALY averted	Unspecified	Antepartum and postpartum periods	16/19 (high quality)

Abbreviations: AL: artemether-lumefantrine, ANC: antenatal care, CHW: community health worker, CTX: cotrimoxazole, DALY: disability-adjusted life year, DRC: Democratic Republic of the Congo, DP: dihydroartemisinin-piperaquine, HIV: human immunodeficiency virus, ICER: incremental cost-effectiveness ratio, IPTp: intermittent preventative treatment, ISTp: individual screening and treatment, ITN: insecticide-treated net, MQ: mefloquine, QALY: quality-adjusted life year, SP: sulfadoxine-pyrimethamine, SST: single screening and treatment.

**Table 4:** Cost-effectiveness findings of studies pertaining to malaria-related anaemia.

Study	Comparator	Intervention	ICER per X	Cost-Effective (threshold)	Author conclusion(s)
Comparing different doses/coverage of IPTp-SP					
Choi et al., 2017 [27]	Two doses of IPTp-SP Low Coverage	Three doses of IPTp-SP Low Coverage	Ghana: US$348 2015 (US$448.38 2024) per 143 DALYs averted Malawi: US$972 2015 (US$1252.38 2024) per 108 DALYs averted Kenya: US$1,097 2015 (US$1,413.44 2024) per 66 DALYs averted Mozambique: US$2,502 2015 (US$3,223.72 2024) per 402 DALYs averted Tanzania: US$2,627 2015 (US$3,394.78 2024) per 37 DALYs averted	Yes (Willingness to pay threshold of less than each country’s GDP per capita) [27]	A regimen providing daily CTX to HIV infected pregnant women living in regions where malaria is endemic is more cost-effective and clinically efficacious than strategies that provide two or three doses of intermittent preventative treatment.
	Two doses of IPTp-SP Low Coverage	Three doses of IPTp-SP High Coverage	Ghana: US$1,602 2015 (US$2,064.11 2024) per 621 DALYs averted Malawi: US$5,400 2015 (US$6,957.68 2024) per 2,289 DALYs averted Kenya: US$5,351 2015 (US$6,894.54 2024) per 817 DALYs averted Mozambique: US$2,739 2015 (US$,3529.09 2024) per 427 DALYs averted Tanzania: US$3,576 2015 (US$4,607.53 2024) per 133 DALYs averted	Yes (as above)	
	Two doses of IPTp-SP Low Coverage	CTX	Ghana: US$0.37 2015 (US$0.48 2024) per DALY averted Malawi: US$0.84 2015 (US$1.08 2024) per DALY averted Kenya: US$1.99 2015 (US$2.56 2024) per DALY averted Mozambique: dominant (CTX dominated two doses of IPTp-SP Low Coverage) Tanzania: US$3.85 2015 (US$4.96 2024) per DALY averted	Yes (as above)	
Fernandes et al., 2015 [31]	Two doses of IPTp-SP	Three or more doses of IPTp-SP	US$7.28 2012 (US$9.82 2024) per DALY averted	Yes (WHO historical willingness to pay thresholds adjusted for inflation (low and middle thresholds) and the mean GDP per capita of the included countries (high threshold) [31,46]	Three or more doses IPTp-SP were cost effective in preventing malaria during pregnancy. These results support WHO guidelines that recommend a monthly dose of IPTp-SP beginning from the second trimester.
Scott et al., 2020 [41]	Baseline coverage of IPTp	95% coverage IPTp	US$9 2020 (US$10.67 2024) per case of anaemia averted As no year for costs was specified, the first year of data collection (2020) was used.	Yes (author discretion)	Increased IPTp coverage led to improved outcomes in areas with significant malaria risk.
IPTp vs other interventions					
Fernandes et al., 2016 [30]	IPTp-SP	ISTp-AL	US-$177.1 2012 (US-$238.9 2024) per DALY averted (ISTP-AL dominated by IPTp-SP)	No (WHO historical willingness to pay thresholds adjusted for inflation) [46]	Changing from IPTp-SP to ISTp-AL was more costly and less effective in reducing negative health outcomes attributable to malaria.
Fernandes et al., 2020 [32]	Three doses of IPTp-SP	Three doses of IPTp-DP	Meta-analysis: US$8 2018 (US$9.78 2024) per DALY averted	Yes (Country based threshold estimates by Woods et al. and Ochalek et al. adjusted for inflation) [45,47]	In areas with a high degree of malaria transmission and SP resistance, IPTp-DP3 and IPTp-DP (monthly) are likely to be highly cost-effective.
	Monthly doses of IPTp-SP	Monthly doses of IPTp-DP	Uganda-II: US$25 2018 (US$30.56 2024) per DALY averted	Yes (as above)	
Hansen et al., 2012 [33]	Two doses of IPTp-SP	Insecticide-treated bed nets (ITNs)	US$54 2004 (US$85.85 2024) per DALY averted	Yes (World Bank historical willingness to pay thresholds adjusted for inflation) [48]	There were no significant differences in health outcomes between the three interventions. Cost-effectiveness analysis did not provide convincing support for replacing current strategies of two-dose IPTp-SP with a combined intervention or ITNs alone.
	Two doses of IPTp-SP	Combined ITNs and IPTp-SP	US$-53 2004 (US-$84.26 2024) per DALY averted (Combined ITNs and IPTp-SP were dominated by two doses of IPTp-SP)	No (as above)	
Paintain et al., 2020 [38]	SST-DP	Monthly IPTp-DP	US$53 2016 (US$67.61 2024) per DALY averted	Yes (WHO historical willingness to pay thresholds adjusted for inflation (low and middle thresholds) and Ochalek et al. (high threshold)) [46,47]	Monthly IPTp-DP represents a cost-effective alternative to SST-DP in Papua.
Sicuri et al., 2015 [42]	Three doses of IPTp-SP (HIV negative women)	Two doses of IPTp-MQ (HIV negative women)	US$136.30 2012 (US$183.86 2024) per DALY averted US$237.78 2012 (US$320.74 2024) per DALY averted if Gabon is included	Yes (World Bank historical willingness to pay thresholds adjusted for inflation) [48]	The addition of IPTp-MQ was very cost-effective for both HIV positive and negative women. However, the poor tolerability of MQ does not favour its use as IPTp.
	Daily CTX, ITNs and IPTp-placebo (HIV positive women)	Daily CTX, ITNs and three doses of IPTp-MQ (HIV positive women)	US$6.96 2012 (US$9.39 2024) per DALY averted	Yes (as above)	
Community vs health-centre delivery of IPTp					
Cirera et al., 2023 [28]	H-IPTp: IPTp-SP administered at ANCs	Combined H-IPTp and C-IPTp: combined health-centre and community-based delivery of IPTp-SP at ANCs and through CHWs	Programmatic mode: - DRC: US$15 2018 (US$18.34 2024) per DALY averted - Madagascar: US$9 2018 (US$11 2024) per DALY averted - Mozambique: US$104 2018 (US$127.14 2024) per DALY averted - Nigeria: US$2 2018 (US$2.45 2024) per DALY averted	Yes (WHO < 3x GDP per capita recommendation) [44]	Combined community and health centre-based delivery of IPTp-SP is a highly cost-effective alternative to health centre only delivery of IPTp.
			TIPTOP mode: -DRC: US$119 2018 (US$145.48 2024) per DALY averted -Madagascar: US$53 2018 (US$64.79 2024) per DALY averted -Mozambique: US$543 2018 (US$663.82 2024) per DALY averted -Nigeria: US$66 2018 (US$80.69 2024) per DALY averted	Yes (WHO < 3x GDP per capita recommendation) [44]	
Mbonye et al., 2008 [36]	H-IPTp	C-IPTp	US$1.10 2003 (US$1.80 2024) per DALY averted	Yes (World Bank historical willingness to pay thresholds adjusted for inflation) [48]	Community rather than health centre-based delivery of IPTp-SP was cost-effective in improving adherence and accessibility.

Abbreviations: AL: artemether-lumefantrine, ANC: antenatal care, CHW: community health worker, CTX: cotrimoxazole, DALY: disability-adjusted life year, DRC: Democratic Republic of the Congo, DP: dihydroartemisinin-piperaquine, GDP: gross domestic product, HIV: human immunodeficiency virus, ICER: incremental cost-effectiveness ratio, IPTp: intermittent preventative treatment, ISTp: individual screening and treatment, ITN: insecticide-treated net, MQ: mefloquine, QALY: quality-adjusted life year, SP: sulfadoxine-pyrimethamine, SST: single screening and treatment, WHO: World Health Organization.

### Iron deficiency-related anaemia

Nine studies [25,26,29,34,35,37,39,40,43] investigated the cost-effectiveness of interventions for the management of iron-deficiency anaemia in pregnancy (Table 1). Studies evaluated the cost-effectiveness of different routes of delivering iron, combination supplements, food fortification and healthcare worker training.

Four studies [25,37,39,40] compared the cost-effectiveness of oral and intravenous iron therapy for pregnant women with iron deficiency anaemia. Three of these studies [37,39,40] were conducted in India and found intravenous iron was cost-effective compared to oral iron therapies in most instances. In two of these studies however [37,40], the effectiveness of intravenous iron appears to have been overestimated compared to recent systematic reviews (Table 1.8 in S8 Appendix). Only one study [25], conducted in the United Arab Emirates, found that intravenous iron therapy was not cost-effective compared to oral iron therapy. This study found that the incremental cost of delivering iron intravenously compared to orally was $138,580.53 per rise to desired Hb, deemed not cost-effective. This study was based on a non-randomised quasi-experimental study where women chose the intervention they wanted to receive and is therefore likely subject to significant bias.

In two studies [34,43], multiple micronutrient supplements (MMS) were found to be cost-effective alternatives to iron and folic acid supplements (IFA). One multi-national study [34] derived effectiveness estimates from two meta-analyses, finding the cost of this transition to be $12.26-$52.99 (Pakistan), $19.12-$40.34 (India) and $13.70-$27.12 (Bangladesh) per DALY averted respectively, depending on which meta-analysis data was used. The second study [43] presented the findings of a modelling tool scaling up MMS in lieu of IFA in 33 countries whilst providing detailed analysis for four countries. Incremental costs in these countries were $6.10-$26.72 (Indonesia), $3.91-$14.75 (Nigeria), $2.09-$10.21 (Pakistan) and $3.76-$17.02 (Tanzania), depending on which meta-analysis data was used. In both studies, MMS was deemed cost-effective compared to IFA in all settings.

Additional pharmacological interventions were evaluated by one study each. One Indian study [29] compared the ACER of branded ($19.19) versus generic ($20.38) ferrous ascorbate per rise in 1 g/dL of haemoglobin. The authors concluded that branded ferrous ascorbate was more favourable overall due to having a lower ACER and fewer reported adverse events. A population model used by one study [26] extrapolated the effects of varying levels of coverage of food fortification and supplementation with iron across Africa, South America, Southeast Asia and Europe. This study found increased rates of fortification and supplementation were cost-effective in all regions compared to no intervention. Whilst supplementation was found to be more effective, increased coverage of fortification from 80% to 95% in Africa was found to be the most cost-effective intervention overall at $34.58 per DALY averted.

Only one study [35] evaluated the cost-effectiveness of a non-pharmacological intervention. This study identified that training frontline healthcare workers to communicate the benefits of IFA supplementation to expectant mothers during antenatal care visits improved uptake and adherence to IFA supplementation in Bangladesh. Compared to standard care, this was found to cost $57.59 per DALY averted and was deemed cost-effective.

### Malaria-related anaemia

Ten studies [27,28,30–33,36,38,41,42] pertaining to malaria-related anaemia in pregnancy were identified (Table 3). All studies evaluated the cost-effectiveness of different antimalarial drug regimens, administrated as either intermittent preventative treatment (IPTp), single screening and treatment (SST) or individual screening and treatment (ISTp). Comparisons were evaluated between drug types, doses and delivery channels. The antimalarial drugs used in these regimens included sulfadoxine pyrimethamine (SP), artemether-lumefantrine (AL), cotrimoxazole (CTX), dihydroartemisinin-piperaquine (DP) and mefloquine (MQ).

Many of the included studies compared the cost-effectiveness of different doses, levels of coverage and ways of delivering antimalarial treatments. Two studies [30,38] compared the cost-effectiveness of administering antimalarials as ISTp and SST rather than IPTp. In both instances, IPTp regimens were found to be economically superior. In one of these studies [30], the effectiveness of ISTp was described as greater than in the literature, potentially underestimating the cost-effectiveness of IPTp even further (Table 2.7 in S8 Appendix). Two studies [32,42] compared the cost-effectiveness of different drugs for use as IPTp. A 2020 study [32] found that both three-dose and monthly IPTp-DP were cost-effective compared to three-dose and monthly IPTp-SP, incurring incremental costs of $9.78 and $30.56 per DALY averted respectively. Three studies [27,31,41] evaluated different doses/coverage of the same drugs. One study [31] found that administering at least three instead of two doses of IPTp-SP improved health outcomes at a modest incremental cost of $9.82 per DALY averted. Another study [41] found that increasing coverage of IPTp-SP to 95% from baseline levels was cost-effective at $10.67 per case of anaemia averted.

Two studies [27,42] evaluated the cost-effectiveness of IPTp versus CTX specifically for pregnant women with HIV in sub-Saharan Africa, with varying results. One study [42] identified that a combination of both CTX and IPTp-MQ was cost-effective compared to CTX alone at $9.39 per DALY averted. However, the authors raised concerns about the poor tolerability of MQ and its suitability for use as IPTp. The second study [27] compared the cost-effectiveness of different doses and levels of coverage of IPTp-SP against each other and daily CTX in five sub-Saharan African countries where malaria was endemic (Ghana, Kena, Malawi, Mozambique and Tanzania). Overall, daily CTX was cost-effective compared to both two or three-dose IPTp-SP. Using daily CTX instead of two-dose IPTp-SP yielded the lowest ICERs; in one country (Mozambique) daily CTX was found to be cost-saving whilst in the remaining four countries modest incremental costs of $0.48-$4.96 per DALY averted were reported.

Community (C-IPTp) instead of/in addition to health centre (H-IPTp) based delivery of IPTp-SP was evaluated by two studies [28,36]. One study [36] found that C-IPTp was more cost-effective than H-IPTp in Uganda at an incremental cost of $1.80 per DALY averted. The second study [28] calculated the cost-effectiveness of using combined C-IPTp and H-IPTp versus H-IPTp alone in four sub-Saharan African countries. Incremental costs per DALY averted varied depending on whether costs were derived from the national health system or estimates from a pilot project and were $11-$64.79 (Madagascar), $127.14-$663.82 (Mozambique), $2.45-$80.69 (Nigeria) and $18.34-$145.48 (Democratic Republic of the Congo (DRC)). In all four countries combined C-IPTp and H-IPTp was deemed cost-effective, however, the effectiveness of the intervention in increasing the coverage of IPTp appears to have been overestimated in three countries (DRC, Madagascar and Nigeria) and underestimated in one country (Mozambique) (Table 2.7 in S8 Appendix).

Only one study [33] compared the cost-effectiveness of pharmacological and non-pharmacological interventions. This study compared the cost-effectiveness of two doses of IPTp-SP with insecticide treated bed nets (ITNs), or a combination of both in Uganda. All three interventions were found to have very similar clinical and cost-effectiveness profiles.

## Discussion

### Main findings

This systematic review identified 19 studies [25–43] that assessed the cost-effectiveness of various interventions to screen, diagnose, prevent or treat anaemia in pregnancy. Only studies pertaining to anaemia secondary to iron-deficiency and malaria were identified. No studies were identified for other aetiologies. Studies were predominately from LMICs. There was significant heterogeneity in terms of interventions compared, cost-effectiveness thresholds and outcomes assessed, which complicated direct comparisons between studies. Overall, most studies used accurate estimates of efficacy, based on or in alignment with most recent evidence from meta-analyses.

### Comparison to existing evidence

Systematic reviews reporting the cost-effectiveness of interventions used for the management of anaemia in pregnancy and its aetiologies are limited. A 2019 systematic review [49] of ten studies that sought to investigate the cost and clinical effectiveness of screening and treating obstetric anaemia failed to identify a single cost-effectiveness study. Similarly, a 2014 systematic review [50] of four studies that evaluated the cost-effectiveness of anaemia screening in vulnerable populations failed to find a single study relating to anaemia in pregnant women. A 2011 systematic review [51] of 48 studies pertaining to the cost-effectiveness of various malaria interventions, identified three studies demonstrating IPTp as the most cost-effective intervention for malaria prevention in pregnant women. This echoes the finding of our review that when compared to alternative interventions, IPTp was cost-effective in most instances. There were no available systematic reviews synthesising the cost-effectiveness of managing iron-deficiency, with or without anaemia, in pregnancy.

Cost-effectiveness evidence from non-pregnant populations provides additional insights into how management of anaemia and its aetiologies in pregnant women might be optimised clinically and economically. In regard to malaria, a 2021 systematic review [52] of 103 costing studies elucidated cost-effectiveness ratios for several preventative interventions for malaria control not assessed by our review that have the potential to be used in reducing anaemia secondary to malaria. For example, indoor residual spraying (IRS) was found to, on average, cost US$25.16 per DALY averted compared to no intervention, however, no final cost-effectiveness determination was made. Additionally, a 2019 systematic review [53] of 15 economic evaluations compared rapid diagnostic tests (RDT) to other means of diagnosing malaria, finding that in ten of these studies RDTs were likely to be cost-effective when evaluated against comparators, with the caveat that this could be influenced by malaria prevalence and type of RDT utilised. Neither of these interventions were evaluated by the studies included in our review.

### Policy implications

This review illuminated several instances where cost-effectiveness evidence affirms most recent clinical guidance. For example, the 2023 *WHO guidelines for malaria* make strong recommendations for the use of IPTp in malaria-endemic areas [54]. Our review demonstrated that IPTp was consistently as or more cost-effective to alternative interventions. These guidelines also advocate for an expansion of personnel able to deliver IPTp-SP, including community health workers [54]. Community health worker delivery of IPTp-SP was found to be cost-effective by both studies in our review that evaluated it. Therefore, based on the findings of our review, both recommendations are supported from an economic perspective.

Our review elucidated several examples as to how potentially economically superior interventions might be suitable for implementation to those currently recommended. For example, the most recent *WHO guidelines for malaria* did not formally consider alternative drug regimens to IPTp-SP, however, our review illustrated that other antimalarials such as IPTp-DP could be cost-effective in certain contexts [54]. This is particularly relevant given rising rates of resistance to SP which necessitates the identification of suitable alternatives such as DP [55]. Regarding women co-infected with malaria and HIV, the *WHO guidelines for malaria* state that there is insufficient evidence to recommend modifying standard malaria treatment recommendations. Our review demonstrated that CTX was a cost-effective alternative to IPTp-SP, suggesting that economically viable alternatives to standard treatments exist. In the *WHO recommendations on antenatal care for a positive pregnancy experience,* WHO does not currently recommend a preferred administration route for iron supplementation [56]. Our review found that in many cases, intravenous rather than oral routes of iron therapy for treatment of iron-deficiency anaemia in pregnancy were cost-effective, with the caveat that this effectiveness appeared to be overestimated in two studies. In doing so, we identified an economically viable alternative to oral iron supplements capable of bypassing prevalent gastrointestinal side effects associated with continuous oral regimens [39]. A 2020 update to these same guidelines*, Nutritional interventions update: Multiple micronutrient supplements during pregnancy,* stated that the cost-effectiveness of MMS compared with IFA supplements varied depending on anaemia prevalence and health outcomes [56]. In both studies included in our review that made this comparison, adopting MMS in favour of IFA supplements was deemed to be cost-effective.

The significance of the cost-effectiveness evidence deficits illuminated by this review are also relevant in clinical and decision-making contexts. For example, the *WHO guidelines for malaria* recommend blood transfusions for immediate management of severe anaemia secondary to malaria, however, our review did not identify a single cost-effectiveness study for this intervention [54]. Vector control, through the deployment of ITNs and IRS were two ‘strong’ recommendations made in these guidelines for use in certain contexts. We only identified one cost-effectiveness study for ITNs, which found it as cost-effective as two doses of IPTp-SP. Cost-effectiveness evidence will be important in determining the feasibility of these recommendations, particularly in regard to their suitability for implementation in low resource settings.

### Research implications

During the process of conducting this review it was apparent that there exists a scarcity of cost-effectiveness evidence for the management of anaemia in pregnancy caused by certain conditions. For example, no studies were identified for interventions used in the management of hookworm; a parasite that infects approximately 44 million pregnancies worldwide, including up to 38% of pregnancies in South-East Asia and an estimated 6.9 million pregnant women in sub-Saharan Africa [57–59]. Similarly, we did not identify any cost-effectiveness analyses for other aetiologies of anaemia such as haemoglobinopathies, autoimmune conditions and bone marrow disorders. Evaluating the cost-effectiveness of interventions used in the management of these and other aetiologies of anaemia in pregnancy, particularly those with a predominance in low resource settings, is a public health priority. Even within the two aetiologies of anaemia evaluated in this review, the small body of cost-effectiveness evidence made it difficult to make conclusive cost-effectiveness determinations, demonstrating the need for further research.

### Strengths and limitations

This review is the first to assess the body of cost effectiveness evidence for the screening, diagnosis, prevention and management of anaemia in pregnancy. We collated data reflecting an array of income levels and clinical contexts for which economic evidence could be evaluated. The breadth of included interventions enabled a full appreciation for the ways in which cost-effectiveness evidence can be incorporated across various stages of managing anaemia in pregnancy.

There were a number of limitations encountered when conducting this review. Firstly, cost-effectiveness data for anaemia secondary to certain common aetiologies, such as hookworm, were not identified. Additionally, it is possible that despite an extensive literature search across a range of databases that some eligible studies were missed. In instances where a composite measure of disease burden such as DALYs were used, studies that failed to specify how anaemia was included in this were excluded. It is possible that in some of these studies, anaemia was a component of this measure but wasn’t reported and was therefore excluded. The findings of this review should therefore be considered in the wider context of management for each of the aetiologies of anaemia. Alongside a diverse range of interventions assessed, there were a range of time horizons, cost-effectiveness thresholds and perspectives employed by different studies, further contributing to the heterogeneity of results. This can make broad interpretation across settings difficult, for example, in some studies (Aftab et al., 2021), a US cost-effectiveness threshold was applied. While potentially appropriate for this study, given it was conducted in a high-income country, this threshold may be too high for application in LMIC settings.

The use of arbitrary thresholds to determine cost-effectiveness, such as the “WHO 3x GDP per cost-effectiveness threshold” cited by several included studies, has been criticised. The failing of this and similar thresholds to account for context specific factors and uncertainty in cost-effectiveness modelling may limit their utility in accurately delineating which interventions are/are not cost-effective [60]. WHO has also advised against using this threshold at the country level for national funding decisions, therefore, bringing into question the robustness of cost-effectiveness determinations made using this threshold [61].

Another limitation of this review arises due to the significant portion of included studies that performed model-based rather than primary cost-effectiveness analyses. In model-based approaches the integration of data from multiple sources may mean that cost-effectiveness ratios do not accurately reflect the local contexts in which they are intended to be applied. This is particularly critical where model-based analyses performed in LMICs have used data from HICs, where factors such as costs, disease burden and healthcare infrastructure are likely to differ significantly [62]. Evaluating and improving the transferability of study results across countries represents an important step in applying the findings of economic evaluations to other settings. Although tools to facilitate this exist, no standardised approach to do so has been universally adopted [63].

Regarding quality assessment, no universally agreed upon methodology/tool to evaluate economic evaluations currently exists [63]. Therefore, while we assessed quality using the validated CHEC-E tool, alternative assessment tools may evaluate different domains to determine study quality, potentially resulting in different quality assessments. Finally, when reviewing effectiveness data described by each included study, we compared only to recent systematic reviews thus excluding data from individual randomised control trials not yet incorporated into meta-analyses, such as the IVON trial [64].

## Conclusion

The findings of this review identify a number of interventions used for the management of anaemia in pregnancy that are both cost-effective and align with or improve upon current best clinical practice. Despite this, the small number of identified studies and the representation of only two aetiologies of anaemia in pregnancy highlight the paucity of available cost-effectiveness evidence for this condition. Greater investment and research are needed to further identify the economic feasibility of interventions used in managing anaemia in pregnancy and to empower relevant stakeholders to be able to optimise care from both clinical and economic perspectives.

## Supporting information

S1 Appendix PRISMA 2020 checklist. (DOCX)

S2 Appendix Search strategy. S2.1 Appendix. Ovid MEDLINE. S2.2 Appendix. CENTRAL. S2.3 Appendix. Embase. S2.4 Appendix. CINAHL Complete. S2.5 Appendix. NHS EED. S2.6 Appendix. EconLit.
(DOCX)

S3 Appendix Data extraction variables. (DOCX)

S4 Appendix Complete data set. (DOCX)

S5 Appendix Excluded studies. (CSV)

S6 Appendix Criteria for quality assessment of included studies using the Extended Consensus Health Economic Criteria (CHEC-E). (DOCX)

S7 Appendix CHEC-E scores. (DOCX)

S8 Appendix Effectiveness comparisons. (DOCX)
