# Supplementary material for: The cost-effectiveness of interventions used for the screening, diagnosis and management of anaemia in pregnancy: A systematic review
Source: PLOS Glob Public Health. 2025 Apr 24;5(4):e0004392. doi: 10.1371/journal.pgph.0004392 (PMC12021152; doi:10.1371/journal.pgph.0004392)
Supplement: S2 Appendix — S2.1 Appendix. Ovid MEDLINE. S2.2 Appendix. CENTRAL. S2.3 Appendix. Embase. S2.4 Appendix. CINAHL Complete. S2.5 Appendix. NHS EED. S2.6 Appendix. EconLit. (DOCX) [file pgph.0004392.s002.docx]

# **S2 Appendix. Search strategy.**

*The initial search was conducted on 25 May 2022 followed by updated searches on 29 August 2023 and 9 August 2024. The search strategy remained identical in all instances.*

# **S2.1 Appendix. Ovid MEDLINE.**

| **#** | **Query** |
| --- | --- |
| 1 | "costs and cost analysis"/ or "cost allocation"/ or cost-benefit analysis/ or health care costs/ or direct service costs/ or drug costs/ or employer health costs/ or hospital costs/ or economics, hospital/ or hospital charges/ or economics, medical/ or fees, medical/ or public expenditures/ |
| 2 | pregnancy/ or pregnancy outcome/ or perinatal care/ or postnatal care/ or preconception care/ or prenatal care/ or obstetrics/ or reproductive health/ or maternal health/ or gravidity/ or pregnancy complications/ or pregnancy in adolescence/ or pregnant women/ or reproductive health/ or reproductive medicine/ or Pregnancy Complications, Hematologic/ |
| 3 | anemia/ or anemia, aplastic/ or anemia, hypoplastic, congenital/ or anemia, hemolytic/ or anemia, hemolytic, autoimmune/ or anemia, hypochromic/ or anemia, iron-deficiency/ or anemia, macrocytic/ or anemia, megaloblastic/ or anemia, pernicious/ or anemia, myelophthisic/ or anemia, refractory/ or anemia, sideroblastic/ or red-cell aplasia, pure/ |
| 4 | (cost* or economic* or hospital charges or medical fees or expenditure* or marginal analy* or affordabilit* or pricing or financ* or hospital management or hospital purchasing or low-value care).mp. |
| 5 | (Pregnanc* or perinatal or postnatal or preconception care or prenatal or obstetric* or reproductive health or matern* or gestation or h?ematological pregnancy complication or antenatal or peripartum or natal or gravidit* or gravida* or multigravid* or primigravid* or parturient* or childbearing or childbirth or child birth* or reproduct* or maternal care or maternity or labo?r or partum failur* or labo?r or conception or birth or parity or puerperium or trimester or womens health).mp. |
| 6 | ((expectant or expecting or gestating) adj (mother* or wom#n or female*)).mp. |
| 7 | (an?emi* or sickle cell or thalass?emi* or h?emoglobinopath* or pancytopenia or h?emoglobin disease or hereditary spherocytosis or hereditary h?emolytic disease or Megaloblastosis or Aplasia or hereditary elliptocytosis or glucosephosphate dehydrogenase deficiency or post-partum h?emorrhage or postpartum h?emorrhage or pph).mp. |
| 8 | exp Anemia/ec [Economics] |
| 9 | 1 or 4 |
| 10 | 2 or 5 or 6 |
| 11 | 3 or 7 or 8 |
| 12 | 9 and 10 and 11 |

# **S2.2 Appendix. CENTRAL.**

| **#** | **Query** |
| --- | --- |
| 1 | "costs and cost analysis"/ or "cost allocation"/ or cost-benefit analysis/ or "cost control"/ or "cost savings"/ or "cost of illness"/ or health care costs/ or direct service costs/ or drug costs/ or employer health costs/ or hospital costs/ or health expenditures/ or capital expenditures/ or low-value care/ or economics, hospital/ or hospital charges/ or economics, medical/ or fees, medical/ |
| 2 | pregnancy complications/ or maternal death/ or pregnancy complications, hematologic/ or obstetrics/ or women's health/ or maternal health/ |
| 3 | anemia/ or anemia, aplastic/ or anemia, hypoplastic, congenital/ or anemia, diamond-blackfan/ or fanconi anemia/ or anemia, hemolytic/ or anemia, hemolytic, autoimmune/ or anemia, hemolytic, congenital/ or anemia, dyserythropoietic, congenital/ or anemia, hemolytic, congenital nonspherocytic/ or anemia, sickle cell/ or acute chest syndrome/ or hemoglobin sc disease/ or sickle cell trait/ or elliptocytosis, hereditary/ or glucosephosphate dehydrogenase deficiency/ or favism/ or hemoglobin c disease/ or spherocytosis, hereditary/ or thalassemia/ or alpha-thalassemia/ or beta-thalassemia/ or delta-thalassemia/ or hemoglobinuria, paroxysmal/ or hemolytic-uremic syndrome/ or atypical hemolytic uremic syndrome/ or anemia, hypochromic/ or anemia, iron-deficiency/ or anemia, macrocytic/ or anemia, megaloblastic/ or anemia, pernicious/ or anemia, myelophthisic/ or anemia, neonatal/ or fetofetal transfusion/ or fetomaternal transfusion/ or anemia, refractory/ or "anemia, refractory, with excess of blasts"/ or anemia, sideroblastic/ or red-cell aplasia, pure/ |
| 4 | (Pregnanc* or perinatal or postnatal or preconception care or prenatal or obstetric* or reproductive health or matern* or gestation or h?ematological pregnancy complication or antenatal or peripartum or natal or gravidit* or gravida* or multigravid* or primigravid* or parturient* or childbearing or childbirth or child birth* or reproduct* or maternal care or maternity or labo?r or partum failur* or labo?r or conception or birth or parity or puerperium or trimester or womens health).mp. |
| 5 | ((expectant or expecting or gestating) adj (mother* or wom#n or female*)).mp. |
| 6 | (an?emi* or sickle cell or thalass?emi* or h?emoglobinopath* or pancytopenia or h?emoglobin disease or hereditary spherocytosis or hereditary h?emolytic disease or Megaloblastosis or Aplasia or hereditary elliptocytosis or glucosephosphate dehydrogenase deficiency or post-partum h?emorrhage or postpartum h?emorrhage or pph).mp. |
| 7 | (cost* or economic* or hospital charges or medical fees or expenditure* or marginal analy* or affordabilit* or pricing or financ* or hospital management or hospital purchasing or low-value care).mp. |
| 8 | 1 or 7 |
| 9 | 2 or 4 or 5 |
| 10 | 3 or 6 |
| 11 | 8 and 9 and 10 |

# **S2.3 Appendix. Embase.**

| **#** | **Query** |
| --- | --- |
| 1 | economic evaluation/ or "cost benefit analysis"/ or "cost control"/ or "cost effectiveness analysis"/ or "cost minimization analysis"/ or "cost of illness"/ or "cost utility analysis"/ or "health care cost"/ or "hospital cost"/ or hospital management/ or hospital finance/ or hospital purchasing/ or "hospital running cost"/ or "hospitalization cost"/ or "drug cost"/ or health care financing/ or "cost"/ |
| 2 | Pregnancy/ or obstetrics/ or childbirth/ or pregnancy disorder/ or pregnancy complication/ or obstetric emergency/ or pregnancy outcome/ or conception/ or parity/ or prenatal development/ or postnatal care/ or puerperium/ |
| 3 | anemia/ or "anemia of chronic disease"/ or aplastic anemia/ or blackfan diamond anemia/ or iron deficiency anemia/ or macrocytic anemia/ or microcytic anemia/ or myelophthisic anemia/ or newborn anemia/ or normochromic normocytic anemia/ or pure red cell anemia/ or refractory anemia/ or refractory anemia with excess blasts/ or sideroblastic anemia/ or hereditary sideroblastic anemia/ or refractory anemia with ringed sideroblasts/ or "refractory cytopenia with multilineage dysplasia and ringed sideroblasts"/ or pearson syndrome/ or x linked sideroblastic anemia/ or megaloblastic anemia/ or folate deficiency anemia/ or pernicious anemia/ or iron refractory iron deficiency anemia/ or plummer vinson syndrome/ or hemolytic anemia/ or acquired hemolytic anemia/ or autoimmune hemolytic anemia/ or coombs positive hemolytic anemia/ or experimental hemolytic anemia/ or heinz body anemia/ or hemolytic uremic syndrome/ or hereditary hemolytic anemia/ or microangiopathic hemolytic anemia/ or paroxysmal nocturnal hemoglobinuria/ or experimental anemia/ or aplastic crisis/ or congenital hypoplastic anemia/ or fanconi anemia/ or pancytopenia/ or hemoglobin c disease/ or hemoglobin d disease/ or hemoglobin h disease/ or hereditary spherocytosis/ or chemotherapy induced anemia/ |
| 4 | (cost* or economic* or hospital charges or medical fees or expenditure* or marginal analy* or affordabilit* or pricing or financ* or hospital management or hospital purchasing or low-value care).mp. |
| 5 | (Pregnanc* or perinatal or postnatal or preconception care or prenatal or obstetric* or reproductive health or matern* or gestation or h?ematological pregnancy complication or antenatal or peripartum or natal or gravidit* or gravida* or multigravid* or primigravid* or parturient* or childbearing or childbirth or child birth* or reproduct* or maternal care or maternity or labo?r or partum failur* or labo?r or conception or birth or parity or puerperium or trimester or womens health).mp. |
| 6 | ((expectant or expecting or gestating) adj (mother* or wom#n or female*)).mp. |
| 7 | (an?emi* or sickle cell or thalass?emi* or h?emoglobinopath* or pancytopenia or h?emoglobin disease or hereditary spherocytosis or hereditary h?emolytic disease or Megaloblastosis or Aplasia or hereditary elliptocytosis or glucosephosphate dehydrogenase deficiency or post-partum h?emorrhage or postpartum h?emorrhage or pph).mp. |
| 8 | 1 or 4 |
| 9 | 2 or 5 or 6 |
| 10 | 3 or 7 |
| 11 | 8 and 9 and 10 |

# **S2.4 Appendix. CINAHL Complete.**

| **#** | **Query** |
| --- | --- |
| 1 | (MH "Costs and Cost Analysis") OR (MH "Cost Benefit Analysis") OR (MH "Cost Control") OR (MH "Health Care Costs") OR (MH "Economic Aspects of Illness") OR (MH "Health Facility Charges") OR (MH "Health Services Purchasing") |
| 2 | (MH "Pregnancy") OR (MH "Obstetric Care") OR (MH "Delivery, Obstetric") OR (MH "Intrapartum Care") OR (MH "Postnatal Care") OR (MH "Prenatal Care") OR (MH "Prepregnancy Care") OR (MH “Postnatal Period”) OR (MH "Obstetric Emergencies") OR (MH "Pregnancy Complications") |
| 3 | (MH "Anemia") OR (MH "Anemia, Hemolytic") OR (MH "Anemia, Hypochromic") OR (MH "Anemia, Macrocytic") OR (MH "Anemia, Neonatal") OR (MH "Hemoglobinopathies") OR (MH "Anemia, Sickle Cell") OR (MH "Thalassemia") OR (MH "Hemolysis") OR (MH "Pregnancy Complications, Hematologic") |
| 4 | Pregnanc* or perinatal or postnatal or preconception care or prenatal or obstetric* or reproductive health or matern* or gestation or h?ematological pregnancy complication or antenatal or peripartum or natal or gravidit* or gravida* or multigravid* or primigravid* or parturient* or childbearing or childbirth or child birth* or reproduct* or maternal care or maternity or labo?r or partum failur* or labo?r or conception or birth or parity or puerperium or trimester or womens health |
| 5 | ((expectant or expecting or gestating) adj (mother* or wom#n or female*)) |
| 6 | (an?emi* or sickle cell or thalass?emi* or h?emoglobinopath* or pancytopenia or h?emoglobin disease or hereditary spherocytosis or hereditary h?emolytic disease or Megaloblastosis or Aplasia or hereditary elliptocytosis or glucosephosphate dehydrogenase deficiency or post-partum h?emorrhage or postpartum h?emorrhage or pph |
| 7 | cost* or economic* or hospital charges or medical fees or expenditure* or marginal analy* or affordabilit* or pricing or financ* or hospital management or hospital purchasing or low-value care |
| 8 | 1 or 7 |
| 9 | 2 or 4 or 5 |
| 10 | 3 or 6 |
| 11 | 8 and 9 and 10 |

# **S2.5 Appendix. NHS EED.**

| **#** | **Query** |
| --- | --- |
| 1 | "cost allocation"/ or cost-benefit analysis/ or "cost control"/ or "cost of illness"/ or "cost sharing"/ or health care costs/ or health expenditures/ or economics, hospital/ or economics, medical/ or fees, medical/ or hospital charges/ or health care sector/ |
| 2 | perinatal care/ or postnatal care/ or prenatal care/ or pregnancy/ or pregnancy complications, hematologic/ or obstetrics/ or pregnant women/ or women's health/ |
| 3 | anemia/ or anemia, aplastic/ or anemia, hypoplastic, congenital/ or anemia, diamond-blackfan/ or fanconi anemia/ or anemia, hemolytic/ or anemia, hemolytic, autoimmune/ or anemia, hemolytic, congenital/ or anemia, hypochromic/ or anemia, iron-deficiency/ or anemia, macrocytic/ or anemia, megaloblastic/ or anemia, pernicious/ or anemia, myelophthisic/ or anemia, refractory/ or "anemia, refractory, with excess of blasts"/ or anemia, sideroblastic/ or red-cell aplasia, pure/ or anemia, sickle cell/ or hemoglobin c disease/ or thalassemia/ or alpha-thalassemia/ or beta-thalassemia/ |
| 4 | (Pregnanc* or perinatal or postnatal or preconception care or prenatal or obstetric* or reproductive health or matern* or gestation or h?ematological pregnancy complication or antenatal or peripartum or natal or gravidit* or gravida* or multigravid* or primigravid* or parturient* or childbearing or childbirth or child birth* or reproduct* or maternal care or maternity or labo?r or partum failur* or labo?r or conception or birth or parity or puerperium or trimester or womens health).mp. |
| 5 | ((expectant or expecting or gestating) adj (mother* or wom#n or female*)).mp. |
| 6 | (an?emi* or sickle cell or thalass?emi* or h?emoglobinopath* or pancytopenia or h?emoglobin disease or hereditary spherocytosis or hereditary h?emolytic disease or Megaloblastosis or Aplasia or hereditary elliptocytosis or glucosephosphate dehydrogenase deficiency or post-partum h?emorrhage or postpartum h?emorrhage or pph).mp. |
| 7 | (cost* or economic* or hospital charges or medical fees or expenditure* or marginal analy* or affordabilit* or pricing or financ* or hospital management or hospital purchasing or low-value care).mp. |
| 8 | 1 or 7 |
| 9 | 2 or 4 or 5 |
| 10 | 3 or 6 |
| 11 | 8 and 9 and 10 |

# **S2.6 Appendix. EconLit.**

| **#** | **Query** |
| --- | --- |
| 1 | Pregnanc* or perinatal or postnatal or preconception care or prenatal or obstetric* or reproductive health or matern* or gestation or h?ematological pregnancy complication or antenatal or peripartum or natal or gravidit* or gravida* or multigravid* or primigravid* or parturient* or childbearing or childbirth or child birth* or reproduct* or maternal care or maternity or labo?r or partum failur* or labo?r or conception or birth or parity or puerperium or trimester or womens health |
| 2 | an?emi* or sickle cell or thalass?emi* or h?emoglobinopath* or pancytopenia or h?emoglobin disease or hereditary spherocytosis or hereditary h?emolytic disease or Megaloblastosis or Aplasia or hereditary elliptocytosis or glucosephosphate dehydrogenase deficiency or post-partum h?emorrhage or postpartum h?emorrhage or pph |
| 3 | cost* or economic* or hospital charges or medical fees or expenditure* or marginal analy* or affordabilit* or pricing or financ* or hospital management or hospital purchasing or low-value care |
| 4 | S1 AND S2 and S3 |
