# Supplementary material for: The cost-effectiveness of interventions used for the screening, diagnosis and management of anaemia in pregnancy: A systematic review
Source: PLOS Glob Public Health. 2025 Apr 24;5(4):e0004392. doi: 10.1371/journal.pgph.0004392 (PMC12021152; doi:10.1371/journal.pgph.0004392)
Supplement: S3 Appendix — (DOCX) [file pgph.0004392.s003.docx]

# **S3 Appendix. Data extraction variables.**

| **Variable** | **Description** |
| --- | --- |
| **Year** | Year of study publication |
| **Author(s)** | Author names |
| **Study name** | Study name |
| **Country** | The country/countries in which the study was conducted |
| **Income level** | As per the World Bank classifications:   - High Income - Upper Middle Income - Lower Middle Income - Low Income - Various |
| **Period of intervention** | The period of pregnancy in which the intervention is utilised, either one or multiple of the following:   - Antenatal - Intrapartum - Postpartum |
| **Facility-level setting** | The setting(s) in which the intervention would be used, examples included:   - Homes - Hospitals - Primary health centres - Specialist outpatient clinics |
| **Study aim** | Aim(s) of the study |
| **Analytic approach/decision model** | Which type of economic evaluation had been conducted and details of any modelling used |
| **Intervention(s)** | Details of interventions |
| **Comparator(s)** | Details of comparators |
| **Number of study arms** | How many arms comprised the study |
| **Aetiology of anaemia related to** | Aetiology of anaemia for which the intervention relates to |
| **Type of intervention** | Broad classification of the type of intervention:   - Lifestyle - Pharmacological - Mechanical/interventional - Various |
| **Health economic measure** | Details of how cost-effectiveness/cost-utility/cost-benefit of interventions were examined:   - Incremental cost-effectiveness ratio - Average cost-effectiveness ratio - Cost-benefit ratio |
| **Clinical outcomes** | Details of specific clinical outcomes recorded |
| **Clinical outcome composites** | Details of any composite outcome measures used:   - DALY - QALY |
| **Outcome measure data source / methodology** | Details of how data pertaining to outcomes were obtained or derived |
| **Source of cost data** | Details of how data pertaining to costs were obtained or derived |
| **Costs included** | Details of which costs were included and examined by each of the studies |
| **Cost-effectiveness time horizon** | The time horizon for which cost-effectiveness was evaluated |
| **Cost-effectiveness perspective** | The perspective for which cost-effectiveness was evaluated |
| **Cost-effectiveness threshold** | The threshold against which was cost-effectiveness was determined |
| **Cost-effectiveness determination** | Whether the intervention was deemed cost-effective |
| **Year of costs** | The year(s) from which costs were derived |
| **Currency of costs** | The currency for which costs were calculated |
| **Key result(s)** | Summary of study results |
| **Author conclusion(s)** | Author conclusions of study results |
| **Abstract** | Study abstracts |
| **DOI / PMID / ISSN** | Additional details of publication (DOI/PMID/ISSN) |
| **Journal** | Journal of publication |
| **Funding** | Source(s) of study funding |
