# Supplementary material for: The cost-effectiveness of interventions used for the screening, diagnosis and management of anaemia in pregnancy: A systematic review
Source: PLOS Glob Public Health. 2025 Apr 24;5(4):e0004392. doi: 10.1371/journal.pgph.0004392 (PMC12021152; doi:10.1371/journal.pgph.0004392)
Supplement: S4 Appendix — (DOCX) [file pgph.0004392.s004.docx]

**S4 Appendix. Complete data set.**

**Table 1. Bibliographic information**

| Study ID | Year | Author(s) | Study name |
| --- | --- | --- | --- |
| #2336 | 2021 | Aftab N.; Faraz S.; Hazari K.; Fahad A.; Alsawalhee N.; Alqedrah A.; Naqvi S.; Paulose L.; Abdelkareem W.; El Gergawi T. | Evaluation of the Impact of Iron Deficiency Anemia during Pregnancy on Hospital Admission and Utilization of Hospital Resources in Latifa Women and Children Hospital, Dubai, UAE |
| #1486 | 2004 | Baltussen, Rob; Knai, Cecile; Sharan, Mona | Iron fortification and iron supplementation are cost-effective interventions to reduce iron deficiency in four subregions of the world |
| #571 | 2017 | Choi, Sung Eun; Brandeau, Margaret L; Bendavid, Eran | Cost-effectiveness of malaria preventive treatment for HIV-infected pregnant women in sub-Saharan Africa |
| #7566 | 2023 | Cirera, Laia; Sacoor, Charfudin; Meremikwu, Martin; Ranaivo, Louise; Manun'Ebo, Manu F; Pons-Duran, Clara; Arikpo, Dachi; Ramirez, Maximo; Ramponi, Francesco; Figueroa-Romero, Antia; Gonzalez, Raquel; Maly, Christina; Roman, Elaine; Sicuri, Elisa; Pagnoni, Franco; Menendez, Clara | Cost-effectiveness of community-based distribution of intermittent preventive treatment of malaria in pregnancy in Madagascar, Mozambique, Nigeria, and the Democratic Republic of Congo |
| #7700 | 2022 | Eeseha, A; Yogita K; Manju T | Pharmacoeconomic Evaluation of Generic Vs Branded preparation of Ferrous Ascorbate in 14 to 24 weeks of Gestational Women |
| #4092 | 2015 | Fernandes S.; Sicuri E.; Kayentao K.; van Eijk A.M.; Hill J.; Webster J.; Were V.; Akazili J.; Madanitsa M.; ter Kuile F.O.; Hanson K. | Cost-effectiveness of two versus three or more doses of intermittent preventive treatment for malaria during pregnancy in sub-Saharan Africa: A modelling study of meta-analysis and cost data |
| #82 | 2016 | Fernandes, Silke; Sicuri, Elisa; Halimatou, Diawara; Akazili, James; Boiang, Kalifa; Chandramohan, Daniel; Coulibaly, Sheikh; Diawara, Sory Ibrahim; Kayentao, Kassoum; Ter Kuile, Feiko; Magnussen, Pascal; Tagbor, Harry; Williams, John; Woukeu, Arouna; Cairns, Matthew; Greenwood, Brian; Hanson, Kara | Cost effectiveness of intermittent screening followed by treatment versus intermittent preventive treatment during pregnancy in West Africa: analysis and modelling of results from a non-inferiority trial |
| #7183 | 2020 | Fernandes S; De Brouwere V; Gutman J; et al | Cost-effectiveness of intermittent preventive treatment with dihydroartemisinin-piperaquine for malaria during pregnancy: an analysis using efficacy results from Uganda and Kenya, and pooled data |
| #1062 | 2012 | Hansen, Kristian Schultz; Ndyomugyenyi, Richard; Magnussen, Pascal; Clarke, Sian E | Cost-effectiveness analysis of three health interventions to prevent malaria in pregnancy in an area of low transmission in Uganda |
| #438 | 2019 | Kashi, Bahman; M Godin, Caroline; Kurzawa, Zuzanna A; Verney, Allison M J; Busch-Hallen, Jennifer F; De-Regil, Luz M | Multiple Micronutrient Supplements Are More Cost-effective Than Iron and Folic Acid: Modeling Results from 3 High-Burden Asian Countries |
| #263 | 2021 | Kurzawa, Zuzanna; Cotton, Christopher S; Mazurkewich, Natasha; Verney, Allison; Busch-Hallen, Jennifer; Kashi, Bahman | Training healthcare workers increases IFA use and adherence: Evidence and cost-effectiveness analysis from Bangladesh |
| #1303 | 2008 | Mbonye, A K; Hansen, K S; Bygbjerg, I C; Magnussen, P | Intermittent preventive treatment of malaria in pregnancy: the incremental cost-effectiveness of a new delivery system in Uganda |
| #10363 | 2023 | Murugesan S; Sudakshina K; Adhimoolam M; Arthi S | Comparative study on efficacy, tolerability, and cost of different iron supplements among antenatal women with iron-deficiency anemia |
| #281 | 2020 | Paintain, Lucy; Hill, Jenny; Ahmed, Rukhsana; Umbu Reku Landuwulang, Chandra; Ansariadi, Ansariadi; Rini Poespoprodjo, Jeanne; Syafruddin, Din; Khairallah, Carole; Burdam, Faustina Helena; Bonsapia, Irene; Ter Kuile, Feiko O; Webster, Jayne | Cost-effectiveness of intermittent preventive treatment with dihydroartemisinin-piperaquine versus single screening and treatment for the control of malaria in pregnancy in Papua, Indonesia: a provider perspective analysis from a cluster-randomised trial |
| #264 | 2020 | Ray, Shomik; Neogi, Sutapa B; Singh, Ranjana; Devasenapathy, Niveditha; Zodpey, Sanjay | Is IV iron sucrose a cost-effective option for treatment of severe anaemia in pregnancy as compared with oral iron? |
| #11132 | 2024 | Saha, S., Raval, D., Shah, K. and Saxena, D | Cost-effectiveness analysis of parenteral iron therapy compared to oral iron supplements in managing iron deficiency anemia among pregnant women |
| #284 | 2020 | Scott, Nick; Delport, Dominic; Hainsworth, Samuel; Pearson, Ruth; Morgan, Christopher; Huang, Shan; Akuoku, Jonathan K; Piwoz, Ellen; Shekar, Meera; Levin, Carol; Toole, Mike; Homer, Caroline Se | Ending malnutrition in all its forms requires scaling up proven nutrition interventions and much more: a 129-country analysis |
| #7228 | 2015 | Sicuri E; Fernandes S; Macete E; et al | Economic evaluation of an alternative drug to sulfadoxine-pyrimethamine as intermittent preventive treatment of malaria in pregnancy |
| #7532 | 2023 | Verney, Allison M J; Busch-Hallen, Jennifer F; Walters, Dylan D; Rowe, Sarah N; Kurzawa, Zuzanna A; Arabi, Mandana | Multiple micronutrient supplementation cost–benefit tool for informing maternal nutrition policy and investment decisions |

**Table 2: Geographic information**

| Study | Country | Multi-country details | Income level |
| --- | --- | --- | --- |
| Aftab et al., (2021) | United Arab Emirates (UAE) | - | High |
| Baltussen et al., (2004) | Multiple | African, South American, European and Southeast Asian subregions; specific countries in supplemental table 1 | Unspecified |
| Choi et al., (2017) | Multiple | Ghana, Kenya, Malawi, Mozambique and Tanzania | Various (Mozambique, Malawi low, Tanzania, Kenya and Ghana lower middle) |
| Cirera et al., (2023) | Multiple | Madagascar, Mozambique, Nigeria, and the Democratic Republic of Congo. | Various (Madagascar, The Democratic Republic of the Congo and Mozambique low, Nigeria lower middle) |
| Eeesha et al., (2022) | India | - | Lower middle |
| Fernandes et al., (2015) | Multiple | Burkina Faso, Kenya, Malawi, Mali, Tanzania, and Zambia | Various (Burkina Faso, Mali, Malayi low, The Gambia, Tanzania and Kenya lower middle |
| Fernandes et al., (2016) | Multiple | Burkina Faso, Ghana, Mali and The Gambia | Various (Burkina Faso, Mali and The Gambia low, Ghana lower middle |
| Fernandes et al., (2020) | Multiple | Uganda and Kenya | Various (Uganda low, Kenya lower middle) |
| Hansen et al., (2012) | Uganda | - | Low |
| Kashi et al., (2019) | Multiple | Pakistan, India and Bangladesh | Lower middle |
| Kurzawa et al., (2021) | Bangladesh | - | Lower middle |
| Mbonye et al., (2008) | Uganda | - | Low |
| Murugesan et al., (2023) | India | - | Lower middle |
| Paintain et al., (2020) | Indonesia | - | Lower middle |
| Ray et al., (2020) | India | - | Lower middle |
| Saha et al., (2024) | India | - | Lower middle |
| Scott et al., (2020) | Multiple | 129 countries | Unspecified |
| Sicuri et al., (2015) | Multiple | Benin, Gabon, Mozambique, Tanzania, Kenya | Various (Benin, Tanzania and Kenya lower middle, Mozambique low, Gabon upper middle) |
| Verney et al., (2023) | Multiple | 33 countries | Various: low, lower middle and upper middle. |

**Table 3. Study aims, intervention mechanisms and analytic approaches**

| Study | Aetiology of Anaemia related to | Intervention mechanism | Period of intervention | Facility-level setting | Aim | Analytic approach/decision model |
| --- | --- | --- | --- | --- | --- | --- |
| Aftab et al., (2021) | Iron deficiency | Pharmacological | Antenatal | Inpatient facility | To evaluate the cost-effect and maternal-fetal outcome of IDA during pregnancy in Latifa Women and Children Hospital, Dubai, UAE | Cost-effectiveness analysis based on a quasi-experimental study |
| Baltussen et al., (2004) | Iron deficiency | Pharmacological and food fortification | Antenatal and postpartum | Multiple | We estimated the costs, effects, and cost-effectiveness of iron supplementation and iron fortification interventions in 4 regions of the world | Cost-effectiveness based on a simulated population model |
| Choi et al., (2017) | Malaria | Pharmacological | Antenatal | Health facility - nurse administered | To assess the cost-effectiveness of CTX compared to IPTp-SP among HIV-infected pregnant women. | Cost-effectiveness analysis of a microsimulation model of malaria and HIV among pregnant women |
| Cirera et al., (2023) | Malaria | Pharmacological | Antenatal | Community (ANC clinics and community health care workers) | We aimed to assess the cost-effectiveness (CE) of C-IPTp in project intervention districts in addition to its delivery at the ANC clinics, compared with distributing IPTp at the ANC clinics alone (standard delivery). | Cost-effectiveness based on a simulated population model |
| Eeesha et al., (2022) | Iron deficiency | Pharmacological | Antenatal | Community and outpatient clinic | Pharmacoeconomic evaluation of branded and generic drug will help to find out the least expensive alternative for treating anaemic pregnant women | Cost-effectiveness analysis based on prospective randomized active control open label study |
| Fernandes et al., (2015) | Malaria | Pharmacological | Antenatal | Health facility - nurse administered | To estimate the incremental cost-effectiveness of IPTp with three or more (IPTp-SP3+) versus two doses of sulfadoxinepyrimethamine (IPTp-SP2). | Cost-effectiveness based on a meta-analysis and cohort simulations |
| Fernandes et al., (2016) | Malaria | Pharmacological | Antenatal | Health facility - nurse administered | The objective of this analysis was to estimate the incremental cost effectiveness of ISTp-AL versus IPTp-SP and then to simulate the effects on cost effectiveness of changes in SP efficacy due to spread of resistance. | Cost-effectiveness analysis based on a multi-centre, non-inferiority trial |
| Fernandes et al., (2020) | Malaria | Pharmacological | Antenatal | Health facility - nurse administered | We aimed to estimate the cost-effectiveness of intermittent preventive treatment in pregnancy with dihydroartemisinin– piperaquine (IPTp-DP) versus IPTp-SP to prevent clinical malaria infection (and its sequelae) during pregnancy. | Cost-effectiveness analysis based three RCTs |
| Hansen et al., (2012) | Malaria | Pharmacological and preventative | Antenatal | Multiple | To determine the cost-effectiveness of IPTp, insecticide-treated bed nets and a combined intervention of both | Cost-effectiveness analysis based on a randomised control trial |
| Kashi et al., (2019) | Iron deficiency | Pharmacological | Antenatal | Outpatient/clinic setting | We evaluated the incremental cost-effectiveness of transitioning from IFA to MMS. | Cost-effectiveness analysis based on meta-analyses and simulated model |
| Kurzawa et al., (2021) | Iron deficiency | Training | Antenatal | Primary health care level | Using a quasi-experimental design, this study investigates the programme's effectiveness and cost-effectiveness at increasing IFA supplement consumption and adherence among pregnant women. | Cost-effectiveness analysis based on a quasi-experimental non-equivalent control group study |
| Mbonye et al., (2008) | Malaria | Pharmacological | Antenatal | Hospital and Community | The main objective of this study was to assess whether traditional birth attendants, drug-shop vendors, community reproductive health workers and adolescent peer mobilisers could administer intermittent preventive treatment (IPTp) with sulfadoxine-pyrimethamine (SP) to pregnant women. | Cost-effectiveness based on interventional study |
| Murugesan et al., (2023) | Iron deficiency | Pharmacological | Antenatal | Community | The aims of this study were to assess the mean change in the hemoglobin levels from baseline up to 60th day of treatment with different iron supplements and to assess its cost effectiveness ratio. | Cost-effectiveness analysis based on a randomised control trial |
| Paintain et al., (2020) | Malaria | Pharmacological | Antenatal | Health facility - nurse administered | We aimed to estimate the incremental cost-effectiveness of intermittent preventive treatment with dihydroartemisinin-piperaquine compared with single screening and treatment with dihydroartemisinin-piperaquine | Cost-effectiveness analysis based on an RCT |
| Ray et al., (2020) | Iron deficiency | Pharmacological | Antenatal | Inpatient facility | The study objective was to conduct a cost-effectiveness analysis of IV iron sucrose over oral therapy for treatment of severe anaemia in pregnancy, alongside the RCT, to inform policy. | Cost-effectiveness analysis based on an RCT |
| Saha et al., (2024) | Iron deficiency | Pharmacological | Antenatal | Primary health care level | The study aimed to compare clinical efficacy and cost- effectiveness of the IVIS therapy with oral iron therapy among pregnant women with IDA in a programmatic setting at Banaskantha and Devbhoomi Dwarka district of Gujarat, India. | Cost-effectiveness analysis based on a prospective study |
| Scott et al., (2020) | Malaria | Various | Antenatal | Various (looking only at interventions for pregnant women however it is community supplementation) | Sustainable Development Goal (SDG) 2.2 calls for an end to all forms of malnutrition, with 2025 targets of a 40% reduction in stunting (relative to 2012), for wasting to occur in less than 5% of children, and for a 50% reduction in anaemia in women (15–49 years). We assessed the likelihood of countries reaching these targets by scaling up proven interventions and identified priority interventions, based on cost-effectiveness. | Cost-effectiveness analysis based on modelling |
| Sicuri et al., (2015) | Malaria | Pharmacological | Antenatal | Health facility - nurse administered | To determine the cost-effectiveness of IPTp with alternative drugs | Cost-effectiveness based on an open label randomised trial (HIV- women) and a double blind placebo controlled trial (HIV+) |
| Verney et al., (2023) | Iron deficiency | Pharmacological | Antenatal | Not specified | The aim of this paper is to describe the underling methodology of the MMS Tool, present the results of a hypothetical MMS scale up scenario for four focus countries and 29 additional countries with pre‐loaded data, and discuss the application of the MMS Tool for supporting the translation of evidence into action. | Cost-effectiveness analysis based on modelling |

**Table 4. Economic and cost information**

| Study | Economic measure | Key outcome composite | Outcome - Clinical | Outcome measure data source / methodology | Source of cost data | Costs included | Cost effectiveness time horizon | CE perspective (description) | Year of costs | Currency of costs |
| --- | --- | --- | --- | --- | --- | --- | --- | --- | --- | --- |
| Aftab et al., (2021) | ICERs | - | Various clinical outcomes (Table 2) | Prospective observational study | Latifa Women and Children Hospital | Cost of treatment  Hospital admission/detention/delivery charges  Laboratory charges  NICU admission Miscellaneous charges | Not specified | Unspecified | Unspecified | USD |
| Baltussen et al., (2004) | ICERs | DALYs | Maternal anaemia | Population model | Cost of fortification is based on the addition of elemental iron powders to cereal flours; this was based on estimates from the US Agency for International Development Micronutrient Program. Costs of iron for supplementation were estimated by following WHO guidelines; drug prices for A regions were retrieved from a web based drugstore, all other regions were retrieved from the international drug price indicator. Various other sources for facility and misc costs. | Cost of iron supplements, cost of antenatal visits, program level costs (central administration, training, health nutrition, education and supervision) Fortified flour costs, program level costs (management, legislation, health nutrition education, supervision costs) | Not specified | Unspecified | 2000 | USD |
| Choi et al., (2017) | ICERs | DALYs | Maternal anaemia, anaemia, LBW, ICERs | Literature - various sources (Table 1) | Literature - various sources | Drug costs, labour costs, antenatal visit costs for patients (direct and indirect) | Not specified | Societal perspective | 2015 | USD |
| Cirera et al., (2023) | ICERs | DALYs | Maternal anaemia, malaria, LBW, neonatal mortality | Literature various sources | Micro-costing or ingredients based costing, data based on project delivery activities | Delivery costs, unit costs | Not specified | Health provider | 2018 | USD |
| Eeesha et al., (2022) | ACERs | - | Rise in Hb, ferritin | Trial data | Not stated | Cost of intervention (supplement) | 14-24 weeks gestation | Unspecified | Unspecified | INR |
| Fernandes et al., (2015) | ICERs | DALYs | LBW, severe/moderate anaemia, clinical malaria | Meta-analysis of seven trials | We calculated cost estimates from data obtained in observational studies, exit surveys, and from public procurement databases | Health care worker costs, drug cost (SP), household costs (direct and indirect antenatal care visit costs) | Lifetime horizon | Societal perspective | 2012 | USD |
| Fernandes et al., (2016) | ICERs | DALYs | LBW, severe/moderate anaemia, clinical malaria | Clinical trail data | Cost estimates were obtained from observational studies, health facility costings and public procurement databases. | Health care worker time costs, drug costs (SP, AL, RDT), costs from consequences | Lifetime horizon | Health provider perspective | 2012 | USD |
| Fernandes et al., (2020) | ICERs | DALYs | Composite of morbidities and neonatal outcomes | Clinical trial data from 3 studies | Cost estimates were obtained from data collected in observational studies, health-facility costings, and from international drug procurement databases. | Health care worker time costs, drug costs (SP and DP), costs from adverse health outcomes | Lifetime horizon | Health care provider perspective | 2018 | USD |
| Hansen et al., (2012) | ICERs | DALYs | LBW, maternal anaemia | Clinical trial data | Five of the seven health centres in the study collected cost data in 2005 | SP administration during ANC, bed net impregnation and distribution, outpatient visit for malaria treatment - all various components | Lifetime horizon | Providers perspective | 2004/2005 | USD |
| Kashi et al., (2019) | ICERs | DALYs | various maternal and neonatal outcomes | World Bank Open Data Base (16), UNICEF (15), WHO Burden of Disease (20), and the Demographic and Health Survey (21) and meta-analyses | UNICEF supply catalogue | Cost of supplements, cost to patient, program cost | Various - averted maternal anaemia was assumed to be 1 year - averted infant ortality and morbidity were lifelong; life expectancy at birth for each country was used | Unspecified | 2016 | USD |
| Kurzawa et al., (2021) | ICERs | DALYs | IFA consumption and adherence | Clinical trial data | Nutrition International costing data | IPC and health promotion material costs (research, modification, production of materials), frontline health care worker training costs | Program period 2012-2014 | Unspecified | 2018 | USD |
| Mbonye et al., (2008) | ICERs | DALYs | anaemia, parasitaemia, low birth weight | Clinical trial data | Interviews with pregnant women, community based resource persons, visits to health centres | SP tablets, costs related to the supply of SP, costs borne by pregnant women | Costs calculated for 6 months of the intervention | Unspecified | 2003/2004 | USD |
| Murugesan et al., (2023) | ACERs | - | Increase in Hb% | Clinical trial data | Pharmacy | Cost of intervention (supplement) | 60 days | Unspecified | 2019/2020 | INR |
| Paintain et al., (2020) | ICERs | DALYs | Fetal loss, neonatal death, LWB, moderate and severe maternal anaemia, clinical malaria | Clinical trial data | Observational studies, health facility costings and public procurement databases | Health worker time cost, midwife's monthly labour cost, drug costs, other costs, costs from consequences | Lifetime horizon | Health provider perspective | 2016 | USD |
| Ray et al., (2020) | ICERs | - | Safe delivery | Clinical trial data | Clinical trial data, literature, personal communication | Hospital costs, user costs | Duration of intervention to 6 weeks postpartum | Limited societal perspective | 2014-2017 but all costs adjusted to the price year of 2018 | INR |
| Saha et al., (2024) | ICERs | QALY | Mean change in Hb level; changes in hemoglobin, place of delivery (institutional or home delivery), normal delivery, cesarean section delivery, pre-term birth, still births, live births, low birth-weight, and normal birth weight babies | Prospective study data | Study data - financial records and field interviews, administrative records | Financial records and field interviews were used to gather costs associated with various heads, including therapy expenses, consumables, healthcare resources, out-of-pocket expenditures, and lost wages etc. Therapy costs for OI and IVIS were gathered from government-approved rate contracts and from rates notified in case of local bulk procurement. Consumables data were collected from the facility, including materials, supplies, quantity used per test, and unit price. Administrative records were reviewed, while research costs were excluded. Travel and wage losses in case of referral or in case of follow-up visits were obtained from field records. | One year | Societal perspective | Unspecified - study ran over 2020-2021 | USD and INR |
| Scott et al., (2020) | ICERs | - | Cases of anaemia averted | Literature | Various sources of literature and databases | - | Not specified | Unspecified | Data from various dates, presented in 2017 USD | USD |
| Sicuri et al., (2015) | ICERs | DALYs | Clinical malaria, anaemia at delivery and non-obstetric hospital admissions | Clinical trial data | Clinical trial data, literature, databases | Labour costs, MQ and SP tablets | Not specified | Health system perspective | Various but expressed in 2012 | USD |
| Verney et al., (2023) | ICERs | DALYs | maternal anaemia (third trimester haemoglobin <110g per litre), preterm delivery, small for gestational age (SGA) newborns (defined by authors of trials), LBW, stillbirths, and maternal, neonatal (death in the first 28 days of life; disaggregated by sex), and infant mortality (death in the first year of life). | Literature | Literature (input into MMS tool) | The unit cost of the supplements and programme transition costs | Lifetime horizon | Unspecified | 2021 | USD |

**Table 5. Iron deficiency anaemia: expanded cost-effectiveness results & sensitivity analyses**

| Study | Treatment Option 1 | Treatment Option 2 | Treatment Option 3 | Treatment Option 4 | ICER | Cost Effectiveness Threshold | Cost Effective (Y/N) | Main Findings | Conclusion | Additional Consideration |
| --- | --- | --- | --- | --- | --- | --- | --- | --- | --- | --- |
| Aftab et al., (2021) | Oral liposomal iron supplementation | IV Iron supplementation | - | - | USD $108,633 per rise to desired Hb | USD $75,000-100,000 | N | The oral liposomal iron was cost-effective when compared to IV iron. The ICER for IV iron was USD 108,633/rise to desire Hb meaning that there is an additional cost of this value that has to be spent to achieve the desired rise in Hb. | Oral liposomal iron may be cost-effective in terms of increases in Hb levels at 4 weeks and have fewer side effects than IV iron. | - |
| Baltussen et al., (2004) | Iron fortification | Iron supplementation | No treatment | - | Compared to 'no treatment': Africa Subregion - Iron fortification 50%: $I 27 - Iron fortification 80%: $I 21 - Iron fortification 95%: $I 20  - Iron supplementation 50%: $I 30 - Iron supplementation 80%: $I 59 - Iron supplementation 95%: $I 66 | - | Y | Iron supplementation has a larger impact than fortification. Supplementation could avert 2.5 million DALYS in the African and Southeast Asian subregions but <12,500 in the European subregion. The cost effectiveness of fortification is preferred economically to iron supplementation. | Iron fortification has the lowest cost effectiveness ratio and is economically more attractive; challenge is ensuring appropriate dissemination and choice of fortified foods which is a key barrier in countries with large, rural populations and for low income groups. Iron supplementation however is more effective in reducing iron deficiency anaemia. When using the benchmark of an icer below 3xGDP they are both cost-effective options however. | Both cost effective when using the 3xGDP as a reference; fortification is cheaper but supplementation more effective |
| Eeesha et al., (2022) | Branded Ferrous Ascorbate | Generic Ferrous Ascorbate | - | - | ACER: Rs. 250.77 for Branded vs 269.38 for generic | - | Y | We observed that there was a significant rise in Hb in both groups but the rise was greater in the Branded group than in the Generic (11.86 +- 0.14 Vs 10.75 +- 0.14, p.001) at the end of 60 days. The average cost-effectiveness ratio (ACER) for Branded group was Rs. 250.77, less than that of Generic Rs 269.38 per increase in Hb gm%. | Ferrous Ascorbate caused significant rise in Hb in gm% in both Branded and Generic groups but Branded Ferrous Ascorbate had better efficacy and was the favourable drug for treatment, as ACER was less and reported less number of adverse events. | ICER displayed visibly, not reported |
| Kashi et al., (2019) | Iron and folic acid supplementation | Multiple micronutrient supplementation | - | - | The Cochrane Scenario (per DALY averted): - USD $41.54 (Pakistan) - USD $31.62 (India) - USD $21.26 (Bangladesh)  The Lancet Scenario (per DALY averted): - USD $9.61 (Pakistan) - USD $14.99 (India) - USD $10.74 (Bangladesh) | WHO 3x GDP per capita rule | Y | The ICER of transitioning from IFA to MMS was 41.54, 31.62, and 21.26 US dollars (USD 2016) per DALY averted for Pakistan, India and Bangladesh respectively. | Despite discrepancies in the overall effect of MMS depending on the meta-analysis used, MMS is cost-effective and generates positive health outcomes for both infants and pregnant women | - |
| Kurzawa et al., (2020) | Standard care | Training healthcare workers | - | - | ICER of USD $47.11 per DALY averted | WHO 3x GDP per capita rule | Y | The difference-in-differences regression analysis comparing outcomes in an intervention and comparison group concluded that the programme increased IFA consumption by an average of 45.05 supplements (P value = 0.018) and increased the share of women that reported adherence to a regime of at least 90 supplements by 40.35 percentage points (P value = 0.020). Knowledge of IFA supplement dosage and benefits also increased among frontline health care workers and pregnant women. The programme cost $47.11 USD (2018) per disability-adjusted life year averted, which is considered highly cost-effective when evaluated against several cost-effectiveness thresholds | This study suggests that the capacity building of frontline health care workers is an effective and cost-effective method of preventing and controlling anaemia among pregnant women in resource-constrained areas. | - |
| Murugesan et al., (2023) | Ferrous sulfate 200 mg twice daily for 60 days | Ferrous ascorbate 200 mg twice daily for 60 days | Ferrous fumarate 200 mg twice daily for 60 days | Iron sucrose 200 mg, based on iron requirement in divided doses and administered once in 2 weeks for a period of 60 days | ACERs: 1: Ferrous sulfate 200mg twice orally for 60 days: Rs. 675 2: Ferrous ascorbate 200mg twice orally for 60 days: Rs. 1782.9 3: Ferrous fumarate 200mg twice orally for 60 days: Rs. 1110.7 4: Iron sucrose 200mg for 60 days as per Hb deficiency status the dose was calculated, administered once every 15 days for 60 days (4 doses in total): Rs. 786.7 | - | Y (ferrous sulfate) | The average cost effectiveness ratio, with respect to Groups 1, 2, 3, and 4 was Rs. 675, Rs. 1782.9, Rs. 1110.7, and Rs. 786.7 per increase in Hb%, respectively. Our results are favoring ferrous sulfate when compared to parenteral iron sucrose economically. | On analyzing the cost-effectiveness ratio, it was found out that the cost incurred per increase in Hb% was less in ferrous sulfate group, followed by iron sucrose, ferrous fumarate, and ferrous ascorbate. | - |
| Ray et al., (2020) | Oral iron supplementation | IV iron supplementation | - | - | ICER of INR 31 951 (USD $445.2) per safe delivery | Half GNI per capita | Y | IV iron sucrose was found to be more costly but more effective than the oral therapy for treatment of severe anaemia. The ICER was calculated at INR 31 951 (USD 445.2) per safe delivery. | We considered a threshold of half the gross national income for decision-making. Considering this threshold of India (INR 57 230, USD 797.4), IV iron-sucrose remained cost-effective in 67% of the iterations in the model. At the current ICER, for every 32 severely anaemic pregnant woman treated with IV iron sucrose one additional pregnant woman will have a safe delivery. Such analyses can complement the national strategy to support evidence-based action. | - |
| Saha et al., (2024) | Intravenous iron sucrose | Iron supplementation | - | - | The incremental cost-effectiveness ratio (ICER) was US$ 9.84 per QALY gained | Approximately 0.49% of India’s per capita GDP | Y | The discounted cost per beneficiary for IVIS was US$ 87, while that for OI was US$ 49. The incremental cost-effectiveness ratio (ICER) was US$ 9.84, which is 0.049% of India’s per capita GDP. | IVIS therapy was more clinically effective and cost-effective than OI therapy among pregnant women for management of moderate and severe anaemia. | To conduct a sensitivity analysis, a one-way approach was adopted. The simulations conducted as part of this analysis are illustrated in Fig. 3. The tornado dia- gram in the one-way sensitivity analysis indicates that the ICER is minimally impacted when certain input parameters are varied. Specifically, the cost of the intervention arm, the incidence of low birth-weight, and pre-term birth reported in the control arm were identified as the key parameters influencing the model |
| Verney et al., (2023) | Iron and folic acid supplementation | Multiple micronutrient supplementation | - | - | Indonesia: $23.55, Nigeria: $13, Pakistan: $9, Tanzania: $15 | WHO GDP per capita threshold | Y | The analysis of transitioning to MMS from IFAS for pregnant women demonstrates substantial additional positive effects with respect to averted child morbidity, as indicated by LBW and SGA and preterm birth, and mortality, as shown by stillbirth and neonatal mortality, in all the four focus countries over 10 years | The cost per DALY averted averages at US$ 23.61 and benefit–cost ratio ranges from US $ 41–US$ 1304: $1.0, which suggest MMS is good value for money compared with IFAS. | BCR: Indonesia: $380, Nigeria: $717, Pakistan: $441, Tanzania: $173 |

**Table 6. Malaria: expanded cost-effectiveness results & sensitivity analyses**

| Study | Treatment Option 1 | Treatment Option 2 | ICER | Cost Effectiveness Threshold | Cost Effective (Y/N) | Expanded cost-effectiveness information | Main Findings | Conclusion | Additional Consideration |
| --- | --- | --- | --- | --- | --- | --- | --- | --- | --- |
| Choi et al., (2017) | 2-IPT Low | 3-IPT Low | Ghana: dominated  Malawi: dominated  Kenya: dominated  Mozambique: dominated  Tanzania: dominated | Willingness to pay (WTP) of less than each country's gross domestic (GDP) per capita (WHO source 38) | Y | - | Compared with the 2-IPT Low Strategy, women receiving CTX had 22.5% fewer LBW infants (95% CI 22.3– 22.7), 13.5% fewer anaemia cases (95% CI 13.4–13.5), and 13.6% fewer maternal malaria cases (95% CI 13.6–13.7). In all simulated countries, CTX was the preferred strategy, with incremental cost-effectiveness ratios ranging from cost saving to $3.9 per DALY averted from a societal perspective. CTX was less effective than the 3-IPT High Strategy when more than 18% of women stopped taking CTX during the pregnancy. | A strategy of providing daily CTX to HIV-infected pregnant women in malaria-endemic regions is generally more effective and less costly than strategies that provide 2 or 3 doses of IPTp-SP. | CTX was less effective than the 3-IPT High Strategy when more than 18% of women stopped taking CTX during the pregnancy |
|  | 2-IPT Low | 3-IPT High | Ghana: dominated  Malawi: dominated  Kenya: dominated  Mozambique: dominated  Tanzania: dominated | Willingness to pay (WTP) of less than each country's gross domestic (GDP) per capita (WHO source 38) | Y | - |  |  |  |
|  | 2-IPT Low | CTX | Ghana: ICER of $0.37  Malawi: ICER of $0.84  Kenya: ICER of $1.99  Mozambique: cost-saving  Tanzania: ICER of $3.85 | Willingness to pay (WTP) of less than each country's gross domestic (GDP) per capita (WHO source 38) | Y | - |  |  |  |
| Cirera et al., (2023) | "Standard" IPTp-SP at ANC clinics | C-IPTp TIPTOP mode (IPTp-SP through ANCs and CHWs) | DRC: $119 MDG: $53 MOZ: $543 NGA: $66 | WHO Threshold (1-3 times GDP per capita) | Y | All CE; all below upper (1x GDP) threshold except MOZ | Net incremental costs of C-IPTp ranged between US$6138–US$47 177 (DRC), US$5552–US$31 552 (MDG), US$10 202–US$53 221 (MOZ) and US$667–US$28 645 (NGA) per 1000 pregnant women, under scenarios (1) and (2), respectively. Incremental cost-effectiveness ratios (ICERs) ranged between US$15–US$119 in DRC, US$9– US$53 in MDG, US$104–US$543 in MOZ and US$2– US$66 in NGA per DALY averted, under scenarios (1) and (2), respectively. ICERs fall below the WHO recommended CE threshold based on the gross domestic product per capita. | Findings suggest that C-IPTp is a highly cost- effective intervention. Results can inform policy decisions on adopting and optimising effective interventions for preventing malaria in pregnancy. | - |
|  | "Standard" IPTp-SP at ANC clinics | C-IPTp Programmatic mode (IPTp-SP through ANCs and CHWs) | DRC: $15 MDG: $9 MOZ: $104 NGA: $2 | WHO Threshold (1-3 times GDP per capita) | Y | All CE: all bellow upper (1x GDP) threshold |  |  |  |
| Fernandes et al., (2015) | 2 doses of IPTp-SP | Monthly doses of IPTp-SP (3 or more) during the second and third trimesters | ICER of $7.28 per DALY averted - HIV- women: $6.2 per DALY averted - Low risk of low birthweight: $19.4 per DALY averted - Moderate risk of low birthweight: $7.7 per DALY averted - High risk of low birthweight: $4.0 per DALY averted | Three frequently applied policymaker willingness-to-pay thersholds: - Low (US$39.72) - Middle (US$238.33) - High (US$756.09) | Y | - | The delivery of IPTp-SP3+ to 1000 pregnant women averted 113·4 DALYs at an incremental cost of$825·67 producing an incremental cost-eff ectiveness ratio (ICER) of $7·28 per DALY averted. | Our findings lend strong support to the WHO guidelines that recommend a monthly dose of IPTp-SP from the second trimester onwards. | - |
| Fernandes et al., (2016) | ISTp-SP | ISTp-AL | ICER of -$177.1 per DALY averted | Three frequently applied policymaker willingness-to-pay thersholds: - Low (US$39.72) - Middle (US$238.33) - High (US$861.33) | N | - | Relative to IPTp-SP, delivering ISTp-AL to 1000 pregnant women cost US$ 4966.25 more (95 % CI US$ 3703.53; 6376.83) and led to a small excess of 28.36 DALYs (95 % CI −75.78; 134.18), with LBW contributing 81.3 % of this difference. The incremental cost-effectiveness ratio was −175.12 (95 % CI −1166.29; 1267.71) US$/DALY averted. | At the current level of SP efficacy and transmission intensity found in this trial in West Africa, switching from IPTp-SP to ISTp-AL is not recommended as ISTp-AL is not more effective and costs considerably more per woman. However, as our modelling suggests, in settings with >10 % prevalence of the sextuple mutant, where IPTp has reduced or no efficacy, ISTp-AL has the potential to be a viable and cost-effective option | The negative ICER is not related to cost savings but because here was a lower (but statistically insignificant) point estimate of efficacy in the AL group. The level at which AL becomes cost effective compared with IPTp is varies depending on the degree to which bed nets are able to reduce the burden of MiP and decision maker WTP. I.E at the highest WTP threshold of $861 per DALY averted ISTP-AL becomes cost-effective at levels of IPTp efficacy ranging from 69 to 79% of current levels. At the lowest threshold of $40 per DALY averted it must fall to 12-36% of current levels. If commodity costs are halved these thresholds change; to between 71 and 80% for the highest and 35-54% efficacy for the lowest. The low and middle WTPs are the historic WHO thresholds of US25 and US150 adjusted for inflation, the high threshold is the unweighted mean GDP per capita calculated across the four countries |
| Fernandes et al., (2020) | 3 doses of IPTp-SP | 3 doses of IPTp-DP | Meta-analysis: ICER of $8 (2 to 29) per DALY averted Kenya: ICER of $7 (2 to 22) per DALY averted Uganda-I: ICER of $-9 (-110 to 93) per DALY averted | Cost-effectiveness thresholds using estimates of country-level thresholds by Woods et al. (27) and Ochalek et al. (28) adjusted for inflation - CET1: Kenya $79.84 , Uganda $30.62 - CET2: Kenya $676.47 , Uganda $411.79 - CET3: Kenya $1273.1 , Uganda $792.95 - CET4: Kenya $520.22 , Uganda $123.96 - CET5: Kenya $685.51 , Uganda $163.17 | Y | Cost-effective in 97.6% of all simulations ran | Compared with three doses of sulfadoxine–pyrimethamine, three doses of dihydroartemisinin–piperaquine, delivered to a hypothetical cohort of 1000 pregnant women, averted 892 DALYs (95% credibility interval 274 to 1517) at an incremental cost of US$7051 (2653 to 13 038) generating an incremental cost-effectiveness ratio (ICER) of $8 (2 to 29) per DALY averted. Compared with monthly doses of sulfadoxine–pyrimethamine, monthly doses of dihydroartemisinin–piperaquine averted 534 DALYS (–141 to 1233) at a cost of $13 427 (4994 to 22 895), resulting in an ICER of $25 (–151 to 224) per DALY averted. Both results were highly robust to most or all variations in the deterministic sensitivity analysis. | In summary, our data suggest that IPTp-DP3 and IPTp-DP monthly are likely to be highly cost-effective in areas of high malaria transmission and sulfadoxine– pyrimethamine resistance, in HIV-negative pregnant women with a high uptake of long-lasting insecticidal nets. | Bracket ranges are 95% credibility estimates IPTP-DP monthly was more costly and less effective than IPTp-DP3 and IPTp-SP3 when all outcomes are included If the model includes a long term cost of low birthweight in the ICER, the ICER of IPTp-DP3 versus IPTP-SP3 changes to $24 per DALY averted when the cost is $1000 and $94 per DALY averted when the cost is $5000 Omitting stillbirth and neonatal death from outcome measurement and then modelling neonatal death from low birth weight with a case fatality rate of 6.93% (29) resulted in IPTp-DP3 being less effective and more costly than IPTp-SP3.  The same modifications resulted in IPTp-DP monthly to produce an ICER of $26 per DALY averted when compared to IPTp-DP3; originally it was less effective and more costly (ICER $-158 per DALY averted) When halving the risk of neonatal death, stillbirth or both in the IPTp-DP monthly group, omitting neonatal death and modelling neonatal death from low birthweight, all lead to IPTp-DP monthly being more cost effective than IPTp-DP3 for all or most cost-effectiveness thresholds Regardless of deterministic changes, IPTp-DP monthly versus IPTp-SP monthly remained cost-effective for at least two cost-effectiveness thresholds with all changes; some changes produced higher but still cost-effective ICERs |
|  | Monthly doses of IPTp-SP | Monthly doses of IPTp-DP | Uganda-II: ICER of $25 (-151 to 224) per DALY averted | Cost-effectiveness thresholds using estimates of country-level thresholds by Woods et al. (27) and Ochalek et al. (28) adjusted for inflation - CET1: Kenya $79.84 , Uganda $30.62 - CET2: Kenya $676.47 , Uganda $411.79 - CET3: Kenya $1273.1 , Uganda $792.95 - CET4: Kenya $520.22 , Uganda $123.96 - CET5: Kenya $685.51 , Uganda $163.17 | Y | Cost-effective in 58.8-93.2% of simulations |  |  |  |
|  | Monthly doses of IPTp-DP | 3 doses of IPTp-SP | Uganda-I: ICER of $-19 (-219 to 192) | Cost-effectiveness thresholds using estimates of country-level thresholds by Woods et al. (27) and Ochalek et al. (28) adjusted for inflation - CET1: Kenya $79.84 , Uganda $30.62 - CET2: Kenya $676.47 , Uganda $411.79 - CET3: Kenya $1273.1 , Uganda $792.95 - CET4: Kenya $520.22 , Uganda $123.96 - CET5: Kenya $685.51 , Uganda $163.17 | N | Cost-effective in 4.8-13.9% of simulations ran |  |  |  |
|  | Monthly doses of IPTp-DP | 3 doses of IPTp-DP | Uganda-I: ICER of $-158 (-146 to 166) | Cost-effectiveness thresholds using estimates of country-level thresholds by Woods et al. (27) and Ochalek et al. (28) adjusted for inflation - CET1: Kenya $79.84 , Uganda $30.62 - CET2: Kenya $676.47 , Uganda $411.79 - CET3: Kenya $1273.1 , Uganda $792.95 - CET4: Kenya $520.22 , Uganda $123.96 - CET5: Kenya $685.51 , Uganda $163.17 | N | Cost-effective in 35.2-46.9% of simulations |  |  |  |
| Hansen et al., (2012) | 2 doses of IPTp-SP | Insecticide-treated bed nets (ITN) | ICER of $54 per DALY averted | Willingness to pay (WTP) thresholds implemented: - Low (US$25) - Middle (US$150) - High (US$500) | Y | Cost effective in 25% of simulations when measured against the low WTP, 64% for the middle one, 69% at the high one | The level of maternal anaemia and the number of LBW babies born in the three intervention arms were not significantly different | In conclusion, in the absence of any demonstrable difference in efficacy between the three interventions on maternal and fetal outcomes,19 this economic evaluation did not provide any additional evidence on economic grounds for replacing IPTp-SP by ITNs alone or by a combined intervention in a setting of low and unstable transmission | The WTPs used are the historical WHO ones albeit not mentioned by the paper The cost-effectiveness did not change markedly even when altering various cost inputs; the largest was when a LLIN was used at a price 30% lower than conventional ITN; it would be cost-effective at 68, 71 and 72% of the low middle and high WTP thresholds respectively |
|  | 2 doses of IPTp-SP | Both ITNs and IPTp-SP | ICER of $-53 per DALY averted | Willingness to pay (WTP) thresholds implemented: - Low (US$25) - Middle (US$150) - High (US$500) | N | Cost-effective in 12% of simulations when compared against the high WTP threshold |  |  |  |
| Mbonye et al., (2008) | Health centre based delivery of ITPp-SP | Community based delivery of IPTp-SP | ICER $1.10 per DALY averted | References: WHO, 1993. Implementation of the global malaria control strategy. World Health Organization, Geneva, Technical Report Series No. 839. Can't find threshold in the text however and none explicitly mentioned. | Y | - | The incremental cost-effectiveness ratio of the community delivery system was Uganda shillings 1869 (US$1.10) per lost disability-adjusted life-year (DALY) averted. | In conclusion, community-based delivery increased access and adherence to IPTp and was cost-effective. | - |
| Paintain et al., (2020) | ISTP-DP | IPTp-DP | ICER $53 per DALY averted | Used the historic WHO thresholds ($25 and $150) adjusted for inflation ($42 and $249)  For the high threshold, Ochalek et al. supply side threshold which for Indonesia was $542 was applied | Y | Cost-effective in 48% of iterations when using the $42 threshold and 'consistent at around 60%' for the middle and upper thresholds of $249 and $542 | Relative to single screening and treatment, intermittent preventive treatment resulted in an incremental cost of US$5657 (95% CI 1827 to 9448) and 107·4 incremental DALYs averted (–719·7 to 904·1) per 1000 women; the average incremental cost-effectiveness ratio was $53 per DALY averted. | Intermittent preventive treatment with dihydroartemisinin-piperaquine offers a cost-effective alternative to single screening and treatment for the prevention of the adverse effects of malaria infection in pregnancy in the context of the moderate malaria transmission setting of Papua. The higher cost of intermittent preventive treatment was driven by monthly administration, as compared with single-administration single screening and treatment. However, acceptability and feasibility considerations will also be needed to inform decision making. | The similarity in the likelihood of IPTP-DP being cost effective at the middle and higher thresholds represents that the use of a considerably higher one times per capita GDP threshold ($3604 for Indonesia in 2016) would not increase the probability of it being cost effective |
| Scott et al., (2020) | IPTp | Standard care | US$9 per case of anaemia averted | Author discretion | Y | - | - | For reducing stunting, IPTp, IYCF education and vitamin A supplementation were the most cost-effective interventions globally. IPTp can lead to improved birth outcomes in areas with malaria risk [66], therefore reducing stunting risk, but it was its low cost rather than high impact that made it the most cost-effective intervention—even at high coverage it produced limited total gains | - |
| Sicuri et al., (2015) | IPTp-SP | 2-dose IPTp-MQ | ICER $136.3 per DALY averted ICER $237.78 per DALY if Gabon is included | World Bank threshold from 1993 adjusted to 2012 prices | Y | - | For HIV-negative women, the ICER for IPTp-MQ versus IPTp-SP was 136.30 US$ (2012 US$) (95%CI 131.41; 141.18) per disability-adjusted life-year (DALY) averted, or 237.78 US$ (95%CI 230.99; 244.57), depending on whether estimates from Gabon were included or not. For HIV-positive women, the ICER per DALY averted for IPTp-MQ added to CTXp, versus CTXp alone was 6.96 US$ (95%CI 4.22; 9.70). | Addition of IPTp with an effective antimalarial to CTXp was very cost-effective in HIV-positive women. IPTp with an efficacious antimalarial was more cost-effective than IPTp-SP in HIV-negative women. However, the poor tolerability of MQ does not favour its use as IPTp. Regardless of HIV status, prevention of malaria in pregnancy with a highly efficacious, well tolerated antimalarial would be cost-effective despite its high price. | In HIV-negative women, moderate shifts of variables such as malaria incidence, drug cost, and IPTp efficacy increased the ICERs above the cost-effectiveness threshold. In HIV-positive women the intervention remained cost-effective for a substantial (up to 21 times) increase in cost per tablet. |
|  | CTXp | 3-dose IPTp-MQ + CTX | ICER $6.96 per DALY averted | World Bank threshold from 1993 adjusted to 2012 prices | Y | - |  |  |  |
