# Supplementary material for: The cost-effectiveness of interventions used for the screening, diagnosis and management of anaemia in pregnancy: A systematic review
Source: PLOS Glob Public Health. 2025 Apr 24;5(4):e0004392. doi: 10.1371/journal.pgph.0004392 (PMC12021152; doi:10.1371/journal.pgph.0004392)
Supplement: S6 Appendix — (DOCX) [file pgph.0004392.s006.docx]

# **S6 Appendix. Criteria for quality assessment of included studies using the Extended Consensus Health Economic Criteria (CHEC-E)**

Source: Odnoletkova I, Goderis G, Pil L, Nobels F, Aertgeerts B, et al. (2014) Cost-Effectiveness of Therapeutic Education to Prevent the Development and Progression of Type 2 Diabetes: Systematic Review. J Diabetes Metab 5: 438 (Appendix)

|  | **Question** |
| --- | --- |
| 1 | Is the study population clearly described? |
| 2 | Are competing alternatives clearly described? |
| 3 | Is a well-defined research question posed in answerable form? |
| 4 | Is the economic study design appropriate to the stated objective? |
| 5 | Are the structural assumptions and the validation methods of the model properly reported? |
| 6 | Is the chosen time horizon appropriate to include relevant costs and consequences? |
| 7 | Is the actual perspective chosen appropriate? |
| 8 | Are all important and relevant costs for each alternative identified? |
| 9 | Are all costs measured appropriately in physical units? |
| 10 | Are costs valued appropriately? |
| 11 | Are all important and relevant outcomes for each alternative identified? |
| 12 | Are all outcomes measured appropriately? |
| 13 | Are outcomes valued appropriately? |
| 14 | Is an incremental analysis of costs and outcomes of alternatives performed? |
| 15 | Are all future costs and outcomes discounted appropriately? |
| 16 | Are all important variables, whose values are uncertain, appropriately subjected to sensitivity analysis? |
| 17 | Do the conclusions follow from the data reported? |
| 18 | Does the study discuss the generalizability of the results to other settings and patient/client groups? |
| 19 | Does the article indicate that there is no potential conflict of interest of study researcher(s) and funder(s)? |
| 20 | Are ethical and distributional issues discussed appropriately? |
