# Supplementary material for: The cost-effectiveness of interventions used for the screening, diagnosis and management of anaemia in pregnancy: A systematic review
Source: PLOS Glob Public Health. 2025 Apr 24;5(4):e0004392. doi: 10.1371/journal.pgph.0004392 (PMC12021152; doi:10.1371/journal.pgph.0004392)
Supplement: S7 Appendix — (DOCX) [file pgph.0004392.s007.docx]

**S7 Appendix. CHEC-E scores.**

| **Author, date** | **Overall rating** | **Patient population** | **Competing alternatives** | **Research question** | **Economic study design** | **Model description** | **Time horizon** | **Study perspective** | **Costs identification** | **Costs measurement** | **Costs valuation** | **Outcomes identification** | **Outcomes measurement** | **Outcomes valuation** | **Incremental CE analysis** | **Discounting** | **Uncertainty analysis** | **Conclusions** | **Generalizability** | **Conflict of interests** | **Ethical issues** |
| --- | --- | --- | --- | --- | --- | --- | --- | --- | --- | --- | --- | --- | --- | --- | --- | --- | --- | --- | --- | --- | --- |
| Aftab, 2021 | **6/18** | Y | Y | Y | N | N/A | N | N | N | N | N | Y | N | N/A | Y | N | N | N | N | Y | N |
| Baltussen, 2004 | **15/20** | Y | Y | Y | Y | Y | Y | N | Y | Y | Y | Y | Y | N | Y | N | Y | Y | Y | N | N |
| Cirera, 2023 | **18/20** | Y | Y | Y | Y | Y | N | Y | N | Y | Y | Y | Y | Y | Y | N | Y | Y | Y | Y | Y |
| Choi, 2017 | **17/20** | Y | Y | Y | Y | Y | N | Y | N | Y | Y | Y | Y | Y | Y | Y | Y | Y | Y | Y | N |
| Eeesha, 2022 | **8/18** | Y | Y | Y | Y | N/A | N | N | Y | N | N | Y | Y | N/A | N | N | N | N | N | Y | N |
| Fernandes, 2020 | **19/20** | Y | Y | Y | Y | Y | Y | Y | Y | Y | Y | Y | Y | Y | Y | Y | Y | Y | Y | Y | N |
| Fernandes, 2016 | **20/20** | Y | Y | Y | Y | Y | Y | Y | Y | Y | Y | Y | Y | Y | Y | Y | Y | Y | Y | Y | Y |
| Fernandes, 2015 | **19/20** | Y | Y | Y | Y | Y | Y | Y | Y | Y | Y | Y | Y | Y | Y | Y | Y | Y | Y | Y | N |
| Hansen, 2012 | **17/19** | Y | Y | Y | Y | N/A | N | Y | Y | Y | Y | N | Y | Y | Y | Y | Y | Y | Y | Y | N |
| Henriquez-Trujillo, 2017 | **13/20** | N | Y | Y | Y | Y | N | N | Y | Y | Y | N | N | Y | Y | Y | N | Y | Y | Y | N |
| Kashi, 2019 | **19/20** | Y | Y | Y | Y | Y | Y | N | Y | Y | Y | Y | Y | Y | Y | Y | Y | Y | Y | Y | Y |
| Kurzawa, 2021 | **14/19** | Y | Y | Y | Y | N/A | N | N | Y | N | N | Y | Y | Y | Y | Y | Y | Y | Y | Y | N |
| Lubinga, 2015 | **19/20** | Y | Y | Y | Y | Y | N | Y | Y | Y | Y | Y | Y | Y | Y | Y | Y | Y | Y | Y | Y |
| Matangila, 2014 | **9/18** | Y | Y | Y | Y | N/A | N | N | N | Y | Y | Y | Y | N/A | N | N | N | N | Y | N | N |
| Mbonye, 2008 | **16/19** | Y | Y | Y | Y | N/A | Y | N | Y | Y | Y | Y | Y | N | Y | Y | Y | Y | N | Y | N |
| Miller, 2015 | **16/19** | Y | Y | Y | Y | N/A | Y | Y | Y | Y | Y | Y | Y | Y | Y | N | Y | Y | N | Y | N |
| Murugesan, 2023 | **7/18** | Y | Y | Y | Y | N/A | N | N | N | N | N | N | Y | N/A | N | Y | N | Y | N | N | N |
| Mvundura, 2016 | **16/19** | Y | Y | Y | Y | N/A | N | N | Y | Y | Y | Y | Y | Y | Y | N | Y | Y | Y | Y | N |
| Paintain, 2020 | **18/19** | Y | Y | Y | Y | N/A | Y | Y | Y | Y | Y | Y | Y | Y | Y | Y | Y | Y | Y | Y | N |
| Prick, 2014 | **14/18** | Y | Y | Y | Y | N/A | Y | Y | Y | Y | Y | N | Y | N/A | Y | N | Y | Y | N | Y | N |
| Ray, 2020 | **16/18** | Y | Y | Y | Y | N/A | Y | Y | Y | Y | Y | Y | Y | N/A | Y | Y | Y | Y | N | Y | N |
| Saha, 2024 | **16/20** | Y | Y | Y | Y | Y | Y | Y | Y | Y | N | N | Y | Y | Y | Y | Y | Y | N | Y | N |
| Scott, 2020 | **15/19** | Y | Y | Y | Y | Y | N | N | Y | Y | Y | Y | Y | N/A | Y | N | N | Y | Y | Y | N |
| Sicuri, 2015 | **17/19** | Y | Y | Y | Y | N/A | U | N | Y | Y | Y | Y | Y | Y | Y | Y | Y | Y | Y | Y | N |
| Sutherland, 2009 | **13/19** | Y | Y | Y | Y | Y | N | N | Y | Y | Y | Y | Y | N/A | Y | N | N | Y | Y | N | N |
| Sutherland, 2013 | **14/20** | N | Y | N | Y | Y | N | N | Y | Y | Y | Y | Y | Y | Y | N | Y | Y | Y | Y | N |
| Sutherland, 2010 | **13/20** | Y | Y | Y | Y | Y | N | N | N | Y | Y | Y | Y | Y | Y | Y | N | Y | N | N | N |
| Verney, 2023 | **18/20** | Y | Y | Y | Y | Y | Y | N | Y | Y | Y | Y | Y | Y | Y | Y | Y | Y | Y | Y | N |
