# Supplementary material for: The cost-effectiveness of interventions used for the screening, diagnosis and management of anaemia in pregnancy: A systematic review
Source: PLOS Glob Public Health. 2025 Apr 24;5(4):e0004392. doi: 10.1371/journal.pgph.0004392 (PMC12021152; doi:10.1371/journal.pgph.0004392)
Supplement: S8 Appendix — (DOCX) [file pgph.0004392.s008.docx]

# **S8 Appendix. Effectiveness comparisons.**

Background and rationale for effectiveness mapping:

Studies calculate the cost-effectiveness of interventions using a combination of economic and effectiveness data. The body of evidence for most interventions’ effectiveness’ has likely grown over time, this could significantly impact the accuracy of cost-effectiveness calculations reported in studies. To ensure that cost-effectiveness calculations in each of our included studies is congruent with most up-to-date effectiveness data, we compared the effectiveness data for each intervention described in included studies to the most recent and robust data available in the literature. Effectiveness data for interventions and outcomes of interest (those used to determine cost-effectiveness) were sought from recent Cochrane and systematic reviews that addressed relevant interventions and outcomes relating to our aetiologies of interest. The degree to which effectiveness data reported by included studies aligned with data from these sources was compared.

This document includes:

- Current WHO recommendations addressing the specific aetiologies of anaemia in pregnancy included in this review.
- A list of interventions identified in our review relevant to the aetiologies encompassed by the above recommendations.
- An overview of the most recent systematic and Cochrane reviews for interventions included in our review available in the literature.
- A comparison of effectiveness data found reported by included studies and those reported by recent systematic and Cochrane reviews.

**Table of Contents**

[1. Iron Deficiency Anaemia 21](#_Toc181094415)

[1.1. Current WHO recommendations for the prevention and management of iron deficiency anaemia in pregnancy 21](#_Toc181094416)

[1.2. Interventions used for the prevention and management of iron deficiency anaemia identified in the cost-effectiveness review 22](#_Toc181094417)

[1.3. Intravenous versus oral iron therapy 24](#_Toc181094418)

[1.4. No intervention versus iron fortification versus iron supplementation 27](#_Toc181094419)

[1.5. Multiple micronutrient supplements versus iron and folic acid supplements. 28](#_Toc181094420)

[1.6. Branded versus generic formulations of iron supplements. 30](#_Toc181094421)

[1.7. Training of health care workers to deliver education around iron supplementation versus no intervention. 31](#_Toc181094422)

[1.8. Comparison of effectiveness data for interventions used for the management of iron deficiency anaemia in pregnancy reported in the literature and the studies included in this review (Table 1.8). 33](#_Toc181094423)

[2. Malaria 44](#_Toc181094424)

[2.1. Current WHO recommendations for the prevention of malaria in pregnancy 44](#_Toc181094425)

[2.2. Current WHO recommendations for the treatment of malaria in pregnancy 45](#_Toc181094426)

[2.3. Interventions used for the prevention and treatment of malaria identified in this review 46](#_Toc181094427)

[2.4. Effectiveness evidence of different doses of IPTp-SP 48](#_Toc181094428)

[2.5. IPTp vs other interventions 51](#_Toc181094429)

[2.6. Community vs health-centre delivery of IPTp 57](#_Toc181094430)

[2.7. Comparison of effectiveness data for interventions used for the prevention and management of malaria-related anaemia in pregnancy reported in the literature and the studies included in this review (Table 2.7). 59](#_Toc181094431)

[References 69](#_Toc181094432)

1. Iron Deficiency Anaemia

- This section provides an overview of:
  - Current WHO recommendations for the prevention and management of iron deficiency anaemia in pregnancy.
  - Interventions used for the prevention and management of iron deficiency anaemia identified in our cost-effectiveness review.
  - Mapping of effectiveness evidence for each intervention included in our cost-effectiveness review.
    - Oral versus intravenous iron.
    - No intervention versus iron fortification versus iron supplementation.
    - Iron and folic acid supplementation versus multiple micronutrient supplementation.
    - Branded versus generic formulations of iron supplements.
    - Training of health care workers to deliver education around iron supplementation versus no intervention.
  1. Current WHO recommendations for the prevention and management of iron deficiency anaemia in pregnancy
- Daily oral iron and folic acid supplementation is recommended for all pregnant women.
  - - Supplementation should generally include 30-60 mg of elemental iron and 400 μg (0.4 mg) of folic acid [1].
    - Where daily supplementation is not tolerated due to side-effects, intermittent oral iron and folic acid supplementation with 120 mg of elemental iron and 2800 μg (2.8 mg) of folic acid once weekly is recommended [1].
    - In settings where anaemia (defined as haemoglobin [Hb] <110 g/L) prevalence among pregnant women is less than 20%, intermittent oral iron and folic acid supplementation with 120 mg of elemental iron and 2800 μg (2.8 mg) of folic acid once weekly is recommended [1].
    - In settings where anaemia prevalence among pregnant women is more than 40%, a daily dose of 60 mg of elemental iron is recommended [1].
    - The equivalent of 60 mg of elemental iron is 300 mg ferrous sulfate heptahydrate, 180 mg ferrous fumarate or 500 mg of ferrous gluconate [1].
- The dose of daily elemental iron should be increased if a diagnosis of anaemia is made during pregnancy [1].
  - - The recommended dose of elemental iron should be 120 mg until Hb concentration rises to at least 110 g/L. Once this is achieved, the standard dose of iron supplementation can be resumed [1].
- Multiple micronutrient supplements are recommended in the context of rigorous research [2].
  1. Interventions used for the prevention and management of iron deficiency anaemia identified in the cost-effectiveness review
     - 7 studies [3-9] in our review included data on the cost-effectiveness of interventions used for the prevention and management of iron deficiency anaemia in pregnancy; they are described below in the following table:

Table 1A: List of interventions used for the prevention or management of iron deficiency anaemia included in our review.

| **Interventions compared** | **Included studies** | **Outcome(s) for which CE was calculated** |
| --- | --- | --- |
| Oral versus intravenous iron | Aftab et al., 2021 [3] | Per rise to desired Hb (unspecified value) |
|  | Ray et al., 2020 [8] | Per additional safe deliveries:   - Defined by the absence of composite maternal and foetal/neonatal adverse clinical outcomes - The composite maternal outcome comprised of postpartum haemorrhage (PPH), need for blood transfusion during delivery and in the postpartum period, puerperal sepsis, shock, a prolonged hospital stay, ICU admission or referral to high centres of care - The composite foetal/neonatal outcome comprised of perinatal death, preterm labour and low birth weight (birth weight <2500 g) |
|  | Murugesan et al., 2023 [7] | Per increase in mean Hb level gm% |
|  | Saha et al., 2024 [9] | Per QALY gained:   - Probability of a normal (vaginal) delivery, c-section, low birth weight, preterm birth, live birth, still birth |
| No intervention versus iron fortification versus iron supplementation | Baltussen et al., 2004 [10] | Per DALY averted:   - Maternal mortality - Perinatal mortaility |
| Iron and folic acid supplementation versus multiple micronutrient supplementation | Kashi et al., 2019 [5] | Per DALY averted:   - Maternal anemia - Preterm delivery - Small for gestional age (SGA) newborns - LBW - Stillbirths - Maternal mortality - Neonatal mortality (death in the first 28 days of life) - Infant mortality (death in the first year of life) |
|  | Verney et al., 2023 [11] | Per DALY averted:   - Maternal anaemia - Preterm delivery - Small for gestional age (SGA) newborns - LBW - Stillbirths - Maternal mortality - Neonatal mortality (death in the first 28 days of life) - Infant mortality (death in the first year of life) |
| Branded versus generic formulations of iron supplements | Eeesha et al., 2022 [4] | Per increase in 1g/dL in Hb |
| Training of health care workers to deliver education around iron supplementation versus no intervention | Kurzawa et al., 2020 [6] | Per DALY averted:   - Maternal anaemia - Preterm delivery - LBW - Maternal mortality - Neonatal mortality |

- 1. Intravenous versus oral iron therapy
     - Two recent systematic reviews [12, 13] and a Cochrane review [14] present effectiveness data for intravenous versus oral iron supplementation in pregnancy for which four studies [3, 7-9] included in our review calculated cost-effectiveness.
       - A 2024 systematic review [13] included 34 RCTs.
       - A 2019 systematic review [12] included 20 RCTs.
       - A 2011 Cochrane review [14] included 23 RCTs of interventions used for the treatment of iron-deficiency anaemia in pregnancy; seven of which compared oral and IV iron.

Table 1.3A: Effectiveness evidence for intravenous versus oral iron therapy relating to haemoglobin: used by two studies [3, 7] to calculate CE.

| **Outcome** | **Statistical method** | **Effect size** | **Source** |
| --- | --- | --- | --- |
| Change in mean Hb from baseline to day 7 | MD* | 0.03 gm/dl | Pandey et al. 2024 [13] |
| Change in mean Hb from baseline to day 14 | MD | 0.55 gm/dl | Pandey et al. 2024 [13] |
| Change in mean Hb from baseline to day 21 | MD | 0.65 gm/dl | Pandey et al. 2024 [13] |
| Change in mean Hb from baseline to day 28 | MD | 0.89 gm/dl | Pandey et al. 2024 [13] |
| Change in mean Hb from baseline to day 42 | MD | 0.89 gm/dl | Pandey et al. 2024 [13] |
| Change in mean Hb from baseline to delivery | MD | 0.78 gm/dl | Pandey et al. 2024 [13] |
| Change in mean Hb from baseline to post delivery | MD | 0.78 gm/dl | Pandey et al. 2024 [13] |
| Hb at delivery | WMD (95% CI) | 0.66 g/dL (0.31, 1.02) | Lewkowitz et al. 2019 [12] |
| Hb 2-6 weeks after treatment | WMD (95% CI) | 0.67 g/dL (0.44, 0.90) | Lewkowitz et al. 2019 [12] |
| Maternal Hb at birth | MD (95% CI) | 0.75 (0.34, 1.16) | Reveiz et al., 2011 [14] |
| Mean maternal Hb at 4 weeks | MD (95% CI) | 0.44 g/dL (0.05, 0.82) | Reveiz et al., 2011 [14] |
| Hb level >12g.dL at 30 days | RR (95% CI) | 0.72 (0.18, 2.87) | Reveiz et al., 2011 [14] |
| Hb level >11g.dL at birth | RR (95% CI) | 1.54 (1.21, 1.94) | Reveiz et al., 2011 [14] |
| Summary   - IV iron therapies either resulted in higher levels of maternal Hb compared to oral treatments or there were no significant differences compared to oral iron treatments. | | | |

WMD: Weighted mean difference, MD: Mean difference, RR: Relative risk, CI: Confidence interval.

*MD were calculated by subtracting the change in mean of mean Haemoglobin levels displayed in Figure 6 in the manuscript by Pandey et al. 2024 [13].

Table 1.3B: Effectiveness evidence for intravenous versus oral iron supplementation relating to still birth: used by one study [9] as a component of a QALY

| **Outcome** | **Statistical method** | **Effect size** | **Source** |
| --- | --- | --- | --- |
| Still birth | OR (95% CI) | 0.92 (0.55, 1.54) | Pandey et al. 2024 [13] |
| Summary   - In one review, there was no difference in still birth between oral and intravenous iron supplements. | | | |

OR: Odds ratio

Table 1.3C: Effectiveness evidence for intravenous versus oral iron supplementation relating to preterm birth and labour: used by one study [9] as a component of a QALY to calculate CE

| **Outcome** | **Statistical method** | **Effect size** | **Source** |
| --- | --- | --- | --- |
| Pre-term births | OR (95% CI) | 0.97 (0.79, 1.18) | Pandey et al. 2024 [13] |
| Summary   - In two reviews, there was no difference in preterm births and labour between oral and intravenous iron supplements. | | | |

OR: Odds ratio

Table 1.3D: Effectiveness evidence for intravenous versus oral iron supplementation relating to low birth weight used by one study [8] as a component of a composite outcome and another study[9] as a component of a QALY to calculate CE

| **Outcome** | **Statistical method** | **Effect size** | **Source** |
| --- | --- | --- | --- |
| Summary   - No effectiveness data for this outcome was available in relevant systematic reviews. | | | |

Table 1.3E: Effectiveness evidence for intravenous versus oral iron supplementation relating to probability of a vaginal delivery: used by one study [9] as a component of a QALY to calculate CE

| **Outcome** | **Statistical method** | **Effect size** | **Source** |
| --- | --- | --- | --- |
| Summary   - No effectiveness data for this outcome was available in relevant systematic reviews. | | | |

Table 1.3F: Effectiveness evidence for intravenous versus oral iron supplementation relating to probability of a caesarean section: used by one study [9] as a component of a QALY to calculate CE

| **Outcome** | **Statistical method** | **Effect size** | **Source** |
| --- | --- | --- | --- |
| Caesarean section | OR (95% CI) | 1.10 (0.86, 1.42) | Pandey et al., 2024[13] |
| Caesarean section | RR (95% CI) | 1.03 (0.94, 1.13) | Lewkowitz et al., 2019[12] |
| Caesarean section | RR (95% CI) | 0.88 (0.46, 1.67) | Reveiz et al., 2011[14] |
| Summary   - There was no significant difference in the rate of caesarean sections between oral and intravenous iron treatment groups in all three reviews. | | | |

RR: Relative risk, OR: Odds ratio

Table 1.3G: Effectiveness evidence for intravenous versus oral iron supplementation relating to probability of a live births: used by one study [9] as a component of a QALY to calculate CE

| **Outcome** | **Statistical method** | **Effect size** | **Source** |
| --- | --- | --- | --- |
| Summary   - No effectiveness data for this outcome was available in relevant systematic reviews. | | | |

- 1. No intervention versus iron fortification versus iron supplementation
     - Two recent systematic reviews [15, 16] compared the effectiveness of no intervention, iron fortification and iron supplementation for which cost-effectiveness was determined in one[10] of our included studies.
       - A 2022 systematic review [16] of 7 RCTs.
       - A 2020 systematic review [15]of 13 RCTs.

Table 1.4A: Effectiveness evidence for no intervention versus iron fortification versus iron supplementation relating to rate of maternal mortality: used by one study [10] as a component of a DALY to calculate CE

| **Outcome** | **Statistical method** | **Effect size** | **Source** |
| --- | --- | --- | --- |
| Summary   - No effectiveness data for this outcome was available in relevant systematic reviews. | | | |

Table 1.4B: Effectiveness evidence for no intervention versus iron fortification versus iron supplementation relating to rate of perinatal mortality: used by one study [10] as a component of a DALY to calculate CE

| **Outcome** | **Statistical method** | **Effect size** | **Source** |
| --- | --- | --- | --- |
| Summary   - No effectiveness data for this outcome was available in relevant systematic reviews. | | | |

- 1. Multiple micronutrient supplements versus iron and folic acid supplements.
     - Two systematic reviews and a Cochrane review [17] present data on this for which two studies in our review determined cost-effectiveness:
       - A 2019 Cochrane review [17] of 19 studies.

Table 1.5A: Effectiveness evidence for multiple micronutrient supplements versus iron and folic acid supplements relating to maternal anaemia: used by two studies [5, 11] as a component of a DALY to determine CE.

| **Outcome** | **Statistical method** | **Effect size** | **Source** |
| --- | --- | --- | --- |
| Maternal anaemia | RR (95% CI) | 1.04 (0.94, 1.15) | Keats et al., 2019 [17] |
| Summary   - - One review found that MMS did not significantly reduce the risk of maternal anaemia compared to IFA. | | | |

RR: Relative risk

Table 1.5B: Effectiveness evidence for multiple micronutrient supplements versus iron and folic acid supplements relating to preterm birth: used by two studies [5, 11] as a component of a DALY to determine CE.

| **Outcome** | **Statistical method** | **Effect size** | **Source** |
| --- | --- | --- | --- |
| Preterm birth | RR (95% CI) | 0.95 (0.90, 1.01) | Keats et al., 2019 [17] |
| Summary   - - Two reviews found that MMS did not significantly reduce the risk of preterm birth compared to IFA. | | | |

RR: Relative risk

Table 1.5C: Effectiveness evidence for multiple micronutrient supplements versus iron and folic acid supplements relating to SGA: used by two studies [5, 11] as a component of a DALY to determine CE.

| **Outcome** | **Statistical method** | **Effect size** | **Source** |
| --- | --- | --- | --- |
| SGA | RR (95% CI) | 0.92 (0.88, 0.97) | Keats et al., 2019 [17] |
| Summary   - - Two reviews found that MMS did not significantly reduce the risk of SGA compared to IFA. | | | |

RR: Relative risk

Table 1.5D: Effectiveness evidence for multiple micronutrient supplements versus iron and folic acid supplements relating to LBW: used by two studies [5, 11] as a component of a DALY to determine CE.

| **Outcome** | **Statistical method** | **Effect size** | **Source** |
| --- | --- | --- | --- |
| LBW | RR (95% CI) | 0.88 (0.85, 0.91) | Keats et al., 2019 [17] |
| Summary   - - Two reviews found that MMS did not significantly reduce the risk of LBW compared to IFA. | | | |

RR: Relative risk

Table 1.5E: Effectiveness evidence for multiple micronutrient supplements versus iron and folic acid supplements relating to still birth: used by two studies [5, 11] as a component of a DALY to determine CE.

| **Outcome** | **Statistical method** | **Effect size** | **Source** |
| --- | --- | --- | --- |
| Stillbirth | RR (95% CI) | 0.95 (0.86, 1.04) | Keats et al., 2019 [17] |
| Summary   - - Two reviews found that MMS did not significantly reduce the risk of stillbirth compared to IFA. | | | |

RR: Relative risk

Table 1.5F: Effectiveness evidence multiple micronutrient supplements versus iron and folic acid supplements relating to maternal mortality: used by two studies [5, 11] as a component of a DALY to determine CE.

| **Outcome** | **Statistical method** | **Effect size** | **Source** |
| --- | --- | --- | --- |
| Maternal mortality | RR (95% CI) | 1.06 (0.72, 1.54) | Keats et al., 2019 [17] |
| Summary   - - One review found that MMS did not significantly reduce the risk of maternal mortality compared to IFA. | | | |

RR: Relative risk

Table 1.5G: Effectiveness evidence multiple micronutrient supplements versus iron and folic acid supplements relating to neonatal mortality: used by two studies [5, 11] as a component of a DALY to determine CE.

| **Outcome** | **Statistical method** | **Effect size** | **Source** |
| --- | --- | --- | --- |
| Neonatal mortality | RR (95% CI) | 1.00 (0.89, 1.12) | Keats et al., 2019 [17] |
| Summary   - - Two reviews found that MMS did not significantly reduce the risk of neonatal mortality compared to IFA. | | | |

RR: Relative risk

Table 1.5H: Effectiveness evidence for multiple micronutrient supplements versus iron and folic acid supplements relating to infant mortality: used by two studies [5, 11] as a component of a DALY to determine CE.

| **Outcome** | **Statistical method** | **Effect size** | **Source** |
| --- | --- | --- | --- |
| Perinatal mortality | RR (95% CI) | 1.00 (0.90, 1.11) | Keats et al., 2019 [17] |
| Summary   - - Two reviews found that MMS did not significantly reduce the risk of perinatal mortality compared to IFA. | | | |

RR: Relative risk

- 1. Branded versus generic formulations of iron supplements.
     - No systematic/Cochrane reviews were identified for this comparison made by one study [4] in our review.

Table 1.6A: Effectiveness evidence for branded versus generic iron supplements relating to Hb: used by one study [4] to determine CE.

| **Outcome** | **Statistical method** | **Effect size** | **Source** |
| --- | --- | --- | --- |
| Summary   - - No systematic review comparing these interventions was identified. | | | |

- 1. Training of health care workers to deliver education around iron supplementation versus no intervention.
     - One systematic review [18] found zero studies comparing the effectiveness of health care worker initiated versus self-management of iron and folic acid supplements during pregnancy which related to how one study [6] included in our review determined cost-effectiveness.

Table 1.7A: Effectiveness evidence for health worker education around iron supplementation during pregnancy vs no intervention relating to maternal mortality: used by one study [6] as part of a DALY.

| **Outcome** | **Statistical method** | **Effect size** | **Source** |
| --- | --- | --- | --- |
| Summary   - No effectiveness data for this outcome was available in relevant systematic reviews | | | |

Table 1.7B: Effectiveness evidence for health worker education around iron supplementation during pregnancy vs no intervention relating to neonatal mortality: used by one study [6] as part of a DALY.

| **Outcome** | **Statistical method** | **Effect size** | **Source** |
| --- | --- | --- | --- |
| Summary   - No effectiveness data for this outcome was available in relevant systematic reviews | | | |

Table 1.7C: Effectiveness evidence for health worker education around iron supplementation during pregnancy vs no intervention relating to maternal anaemia: used by one study [6] as part of a DALY.

| **Outcome** | **Statistical method** | **Effect size** | **Source** |
| --- | --- | --- | --- |
| Summary   - No effectiveness data for this outcome was available in relevant systematic reviews | | | |

Table 1.7D: Effectiveness evidence for health worker education around iron supplementation during pregnancy vs no intervention relating to preterm birth: used by one study [6] as part of a DALY.

| **Outcome** | **Statistical method** | **Effect size** | **Source** |
| --- | --- | --- | --- |
| Summary   - No effectiveness data for this outcome was available in relevant systematic reviews | | | |

Table 1.7E: Effectiveness evidence for health worker education around iron supplementation during pregnancy vs no intervention relating to LBW: used by one study [6] as part of a DALY.

| **Outcome** | **Statistical method** | **Effect size** | **Source** |
| --- | --- | --- | --- |
| Summary   - No effectiveness data for this outcome was available in relevant systematic reviews | | | |

- 1. Comparison of effectiveness data for interventions used for the management of iron deficiency anaemia in pregnancy reported in the literature and the studies included in this review (Table 1.8).

| Included study (Intervention/s & Comparator/s) | Outcome(s) used to calculate CE | Model parameters/outcome probabilities/effectiveness evidence cited in each study | Source of effectiveness evidence cited in each study | Corresponding effectiveness evidence from recent systematic reviews identified in this report | Comparison between effectiveness evidence from studies included in our cost-effectiveness review with recent effectiveness systematic reviews | Comments on similarities/discrepancies with effectiveness evidence from recent systematic reviews |
| --- | --- | --- | --- | --- | --- | --- |
| Intravenous vs oral iron treatment | | | | | | |
| Aftab et al., 2021 [3]   1. Oral liposomal iron 2. Intravenous iron sucrose | % of patients who achieved desired Hb (unspecified value) after 1 month of treatment | OI: 84.4%  IVIS: 90%  Calculated RR: 1.06 | Study data | Table 1.3A | This study does not specify what desired Hb used to measure intervention effectiveness is. As a result, identical corresponding effectiveness data was not able to be sought. Similar data described below was available.  The RR used in this study is **higher** than reported by Reveiz et al., 2011 [14] for Hb level >12g/dL at 30 days (0.72).  The RR used in this study falls **within** of the 95% CI reported by Reveiz et al., 2011 [14] for Hb level >12g/dL at 30 days (0.18, 2.87). | The effectiveness described by the study is **congruent** with current evidence. |
| Ray et al., 2020 [8]   1. Oral iron 2. Intravenous iron sucrose | Probability (95%CI) of a safe delivery   - Safe delivery is defined as the absence of composite maternal and foetal/neonatal adverse clinical outcomes. | OI: 0.481 (0.417, 0.546)  IVIS: 0.467 (0.400, 0.534) | Neogi et al., 2019 [19] | N/A | The composite outcome used to determine cost-effectiveness in this study was not found elsewhere in the literature, therefore, corresponding effectiveness data was not able to be sought. | N/A |
| Murugesan et al., 2023 [7]   1. Ferrous sulfate 200mg twice orally for 60 days 2. Ferrous ascorbate 200mg twice orally for 60 days 3. Ferrous fumarate 200mg twice orally for 60 days 4. Iron sucrose 200mg for 60 days as per Hb deficiency status the dose was calculated, administered once every 15 days for 60 days (4 doses in total) intravenously | MD (95%CI) in mean Hb level gm% from baseline to the end of treatment | Ferrous sulfate: 0.53 (0.30, 0.75)  Ferrous ascorbate: 0.73 (0.43, 1.03)  Ferrous fumarate: 0.92 (0.58, 1.26)  IVIS: 2.34 (2.05, 2.63) | Study data | Table 1.3A | The MD representing the effectiveness of IVIS in this study is **significantly higher** than effectiveness estimates described by Lewkowitz et al., 2019 [12] (WMD: 0.66 [at delivery] & 0.67 [2-6 weeks after treatment]) and Reveiz et al., 2011 [14] (MD: 0.75 [at birth] & 0.44 [at 4 weeks]).  The MD representing the effectiveness of IVIS in this study falls **outside** of the 95% CI described by Lewkowitz et al., 2019 [12] (0.31, 1.02 [at delivery] & 0.44, 0.90 [2-6 weeks after treatment]) and Reveiz et al., 2011 [14] (0.34, 1.16 [at birth] & 0.05, 0.82 [at 4 weeks]). | The effectiveness of IVIS in increasing mean Hb levels may have been **significantly** **overestimated** in this study.  This may have resulted in IVIS appearing **significantly more cost-effective.** |
| Saha et al., 2024 [9]   1. Oral iron 2. Intravenous iron sucrose | QALYs gained | | | | | |
|  | Probability of still birth | OI: 0.052 (3 out of 58)  IVIS: 0.000 (0 out of 64)  Calculated OR: 0 | Study data | Table 1.3B | The OR used in this study is **lower** than the OR reported in Pandey et al. 2024 [13] (0.92) and falls **outside** of the 95% CI (0.55, 1.54). | The effectiveness of IVIS in reducing the probability of still birth may have been **overestimated** in this study.  This may have resulted in IVIS appearing **more cost-effective** compared to OI. |
|  | Probability of preterm birth: | OI: 0.155 (9 out of 58)  IVIS: 0.109 (7 out of 64)  Calculated RR: 0.703  Calculated OR: 0.668 | Study data | Table 1.3C | The OR used in this study is **lower** than the OR reported in Pandey et al. 2024 [13] (0.97) and falls **outside** of the 95% CI (0.79, 1.18). | The effectiveness of IVIS in reducing the probability of preterm birth may have been **overestimated** in this study.  This may have resulted in IVIS appearing **more cost-effective** compared to OI. |
|  | Probability of low birth weight: | OI: 0.100 (5 out of 46)  IVIS: 0.109 (5 out of 57)  Calculated RR: 1.090 | Study data | Table 1.3D | No effectiveness data for this outcome was available in relevant systematic reviews. | N/A |
|  | Probability of a normal (vaginal) delivery | OI: 0.760 (44 out of 58)  IVIS: 0.850 (61 out of 72) | Study data | Table 1.3E | No effectiveness data for this outcome was available in relevant systematic reviews. | N/A |
|  | Probability of C-section | OI: 0.240 (14 out of 58)  IVIS: 0.150 (11 out of 72)  Calculated RR: 0.625  Calculated OR: 0.566 | Study data | Table 1.3F | The OR used in the study is **lower** than the OR reported in Pandey et al. 2024 [13] (1.10). This falls **outside** of the 95% CI in Pandey et al. 2024 [13] (0.86, 1.42).  The RR used in the study is **lower** than the RR reported in Lewkowitz et al. 2019 [12] (1.03) and Reveiz et al., 2011 [14] (0.88).  This falls **outside** of the 95% CI in Lewkowitz et al. 2019 [12] (0.94, 1.13) but **within** the 95% CI in Reveiz et al., 2011 [14] (0.46, 1.67). | The effectiveness of IVIS in reducing the probability of caesarean sections may have been **overestimated** in this study.  This may have resulted in IVIS appearing **more cost-effective** compared to OI. |
|  | Probability of live birth | OI: 0.793 (46 out of 58)  IVIS: 0.891 (57 out of 64) | Study data | Table 1.3G | No effectiveness data for this outcome was available in relevant systematic reviews. | N/A |
| No intervention vs iron fortification vs iron supplementation | | | | | | |
| Baltussen et al., 2004 [10]   1. No intervention 2. Iron supplementation 95% | DALYs averted: | | | | | |
|  | Probability of maternal mortality | African subregion   - No intervention: 0.0124. - Iron: 0.0107.   South American subregion   - No intervention: 0.0027. - Iron: 0.0025.   European subregion   - No intervention: 0.0000. - Iron: 0.0000.   Southeast Asian subregion   - No intervention: 0.0053. - Iron: 0.0043. | Stoltzfus et al., 2003 [20] | Table 1.4A | No effectiveness data for this outcome was available in relevant systematic reviews. | N/A |
|  | Probability of perinatal mortality | African subregion   - No intervention: 0.0466. - Iron: 0.0369.   South American subregion   - No intervention: 0.0089. - Iron: 0.0083.   European subregion   - No intervention: 0.0020. - Iron: 0.0019.   Southeast Asian subregion   - No intervention: 0.0325. - Iron: 0.0277. | Stoltzfus et al., 2003 [20] | Table 1.4B | No effectiveness data for this outcome was available in relevant systematic reviews. | N/A |
| Multiple micronutrient supplements versus iron and folic acid supplements | | | | | | |
| Kashi et al., 2019 [5]   1. Iron and folic acid supplements 2. Multiple micronutrient supplements | DALYs averted | | | | | |
|  | RR (SE) of maternal anaemia | MMS vs IFA: 1.03 (0.11) | Haider et al., 2017 [21] | Table 1.5A | The RR used in this study is almost **identical** to the RR described by Keats et al., 2019 [17] (1.04) and falls **within** the 95% CI (0.94, 1.15). | The effectiveness described by the study is **congruent** with current evidence. |
|  | RR (SE) of preterm delivery | MMS vs IFA: 0.96 (0.04) | Haider et al., 2017 [21] | Table 1.5B | The RR used in this study is almost **identical** to the RR described by Keats et al., 2019 [17] (1.04) and falls **within** the 95% CI (0.94, 1.15). | The effectiveness described by the study is **congruent** with current evidence. |
|  |  | MMS vs IFA: 0.92 (0.02) | Smith et al., 2017 [22] | Table 1.5B | The RR used in this study is almost **lower** to the RR described by Keats et al., 2019 [17] (1.04) and falls **outside** the 95% CI (0.94, 1.15). | The effectiveness described by the study is **congruent** with current evidence. |
|  | RR (SE) of SGA | MMS vs IFA: 0.92 (0.03) | Haider et al., 2017 [21] | Table 1.5C | The RR used in this study is **identical** to the RR described by Keats et al., 2019 [17] (0.92). | The effectiveness described by the study is **congruent** with current evidence. |
|  |  | MMS vs IFA: 0.97 (0.01) | Smith et al., 2017 [22] | Table 1.5C | The RR used in this study is almost **identical** to the RR described by Keats et al., 2019 [17] (0.92) and falls **within** the 95% CI (0.88, 0.97). | The effectiveness described by the study is **congruent** with current evidence. |
|  | RR (SE) of LBW | MMS vs IFA: 0.88 (0.02) | Haider et al., 2017 [21] | Table I.5D | The RR used in this study is **identical** to the RR described by Keats et al., 2019 [17] (0.88). | The effectiveness described by the study is **congruent** with current evidence. |
|  |  | MMS vs IFA: 0.88 (0.01) | Smith et al., 2017 [22] | Table I.5D | The RR used in this study is **identical** to the RR described by Keats et al., 2019 [17] (0.88). | The effectiveness described by the study is **congruent** with current evidence. |
|  | RR (SE) of stillbirths | MMS vs IFA: 0.97 (0.06) | Haider et al., 2017 [21] | Table I.5E | The RR used in this study is almost **identical** to the RR described by Keats et al., 2019 [17] (0.95) and falls **within** the 95% CI (0.86, 1.04). | The effectiveness described by the study is **congruent** with current evidence. |
|  |  | MMS vs IFA: 0.92 (0.04) | Smith et al., 2017 [22] | Table I.5E | The RR used in this study is almost **identical** to the RR described by Keats et al., 2019 [17] (0.95) and falls **within** the 95% CI (0.86, 1.04). | The effectiveness described by the study is **congruent** with current evidence. |
|  | RR (SE) of maternal mortality | MMS vs IFA: 0.97 (0.26) | Haider et al., 2017 [21] | Table 1.5F | The RR used in this study is almost **identical** to the RR described by Keats et al., 2019 [17] (1.06) and falls **within** the 95% CI (072, 1.54). | The effectiveness described by the study is **congruent** with current evidence. |
|  | RR (SE) of neonatal mortality | MMS vs IFA: 1.06 (0.08) | Haider et al., 2017 [21] | Table 1.5G | The RR used in this study is almost **identical** to the RR described by Keats et al., 2019 [17] (1.00) and falls **within** the 95% CI (0.89, 1.12). | The effectiveness described by the study is **congruent** with current evidence. |
|  |  | MMS vs IFA: 0.98 (0.04) | Smith et al., 2017 [22] | Table I.5G | The RR used in this study is almost **identical** to the RR described by Keats et al., 2019 [17] (1.00) and falls **within** the 95% CI (0.89, 1.12). | The effectiveness described by the study is **congruent** with current evidence. |
|  | RR (SE) of infant mortality | MMS vs IFA: 0.95 (0.06) | Haider et al., 2017 [21] | Table 1.5H | The RR used in this study is almost **identical** to the RR described by Keats et al., 2019 [17] (1.00) and falls **within** the 95% CI (0.90, 1.11). | The effectiveness described by the study is **congruent** with current evidence. |
|  |  | MMS vs IFA: 0.97 (0.05) | Smith et al., 2017 [22] | Table 1.5H | The RR used in this study is almost **identical** to the RR described by Keats et al., 2019 [17] (1.00) and falls **within** the 95% CI (0.90, 1.11). | The effectiveness described by the study is **congruent** with current evidence. |
| Verney et al., 2023 [11]   1. Iron and folic acid supplements 2. Multiple micronutrient supplements | DALYs averted | | | | | |
|  | RR (95% CI) of maternal anaemia | MMS vs IFA: 1.04 (0.94, 1.15) | Keats et al., 2019 [17] | Table 1.5A | The RR used in this study is the RR described by Keats et al., 2019 [17] | The effectiveness described by the study is **congruent** with current evidence. |
|  | RR (95% CI) of preterm delivery | MMS vs IFA: 0.95 (0.90, 1.01) | Keats et al., 2019 [17] | Table 1.5B | The RR used in this study is the RR described by Keats et al., 2019 [17] | The effectiveness described by the study is **congruent** with current evidence. |
|  |  | MMS vs IFA: 0.92 (0.88, 0.95) | Smith et al., 2017 [22] | Table 1.5B | The RR used in this study is **almost identical** to the RR described by Keats et al., 2019 [17] (0.95) and falls **within** the 95% CI (0.90, 1.01). | The effectiveness described by the study is **congruent** with current evidence. |
|  | RR (95% CI) of SGA | MMS vs IFA: 0.92 (0.88, 0.97) | Keats et al., 2019 [17] | Table 1.5C | The RR used in this study is the RR described by Keats et al., 2019 [17] | The effectiveness described by the study is **congruent** with current evidence. |
|  |  | MMS vs IFA: 0.97 (0.96, 0.99) | Smith et al., 2017 [22] | Table 1.5C | The RR used in this study is **almost** **identical** to the RR described by Keats et al., 2019 [17] (0.92) and falls **within** the 95% CI (0.88, 0.97). | The effectiveness described by the study is **congruent** with current evidence. |
|  | RR (95% CI) of LBW | MMS vs IFA: 0.88 (0.85, 0.91) | Keats et al., 2019 [17] | Table I.5D | The RR used in this study is the RR described by Keats et al., 2019 [17] | The effectiveness described by the study is **congruent** with current evidence. |
|  |  | MMS vs IFA: 0.88 (0.85, 0.90) | Smith et al., 2017 [22] | Table I.5D | The RR used in this study is **identical** to the RR described by Keats et al., 2019 [17] (0.88). | The effectiveness described by the study is **congruent** with current evidence. |
|  | RR (95% CI) of stillbirths | MMS vs IFA: 0.95 (0.86, 1.04) | Keats et al., 2019 [17] | Table I.5E | The RR used in this study is the RR described by Keats et al., 2019 [17] | The effectiveness described by the study is **congruent** with current evidence. |
|  |  | MMS vs IFA: 0.92 (0.86, 0.99) | Smith et al., 2017 [22] | Table I.5E | The RR used in this study is almost **identical** to the RR described by Keats et al., 2019 [17] (0.95) and falls **within** the 95% CI (0.86, 1.04). | The effectiveness described by the study is **congruent** with current evidence. |
|  | RR (95% CI) of maternal mortality | MMS vs IFA: 1.06 (0.72, 1.54) | Keats et al., 2019 [17] | Table 1.5F | The RR used in this study is the RR described by Keats et al., 2019 [17] | The effectiveness described by the study is **congruent** with current evidence. |
|  | RR (95% CI) of neonatal mortality | MMS vs IFA: 1.00 (0.89, 1.12) | Keats et al., 2019 [17] | Table 1.5G | The RR used in this study is the RR described by Keats et al., 2019 [17] | The effectiveness described by the study is **congruent** with current evidence. |
|  |  | MMS vs IFA: 0.98 (0.90, 1.05) | Smith et al., 2017 [22] | Table I.5G | The RR used in this study is **almost** **identical** to the RR described by Keats et al., 2019 [17] (1.00) and falls **within** the 95% CI (0.89, 1.12). | The effectiveness described by the study is **congruent** with current evidence. |
|  | RR (95% CI) of infant mortality | MMS vs IFA: 1.00 (0.90, 1.11) | Keats et al., 2019 [17] | Table 1.5H | The RR used in this study is the RR described by Keats et al., 2019 [17] | The effectiveness described by the study is **congruent** with current evidence. |
|  |  | MMS vs IFA: 0.97 (0.88, 1.06) | Smith et al., 2017 [22] | Table 1.5H | The RR used in this study is almost **identical** to the RR described by Keats et al., 2019 [17] (1.00) and falls **within** the 95% CI (0.90, 1.11). | The effectiveness described by the study is **congruent** with current evidence. |
| Branded versus generic formulations of iron supplements | | | | | | |
| Eeesha et al., 2022 [4]   1. Branded iron supplements 2. Generic iron supplements | MD in Hb after 30 days of treatment | Branded iron: 2.45 g/dL  Generic iron: 1.225 g/dL | Study data | Table 1.6A | No systematic review comparing these interventions was identified. | N/A |
| Training of health care workers to delivery education around iron supplementation versus no intervention | | | | | | |
| Kurzawa et al., 2021 [6]   1. No intervention 2. Training of health care workers to delivery education around iron supplementation | DALYs averted | | | | | |
|  | RR (SE) of maternal mortality | 0.33 (1.69) | Peña-Rosas et al., 2015 [23] | Table 1.7A | No effectiveness data for this outcome was available in relevant systematic reviews | N/A |
|  | RR (SE) of neonatal mortality | 0.91 (0.25) | Peña-Rosas et al., 2015 [23] | Table 1.7B | No effectiveness data for this outcome was available in relevant systematic reviews | N/A |
|  | RR (SE) of maternal anaemia | 0.30 (0.25) | Peña-Rosas et al., 2015 [23] | Table 1.7C | No effectiveness data for this outcome was available in relevant systematic reviews | N/A |
|  | RR (SE) of preterm birth | 0.93 (0.11) | Peña-Rosas et al., 2015 [23] | Table 1.7D | No effectiveness data for this outcome was available in relevant systematic reviews | N/A |
|  | RR (SE) of LBW | 0.84 (0.08) | Peña-Rosas et al., 2015 [23] | Table 1.7E | No effectiveness data for this outcome was available in relevant systematic reviews | N/A |

Abbreviations: IVIS: IV Iron sucrose, OI: Oral iron, MMS: Multiple micronutrient supplements, IFA: Iron and folic acid supplements

*Baltussen et al., compared seven interventions however only provided effectiveness data for two: no intervention and 95% iron supplementation

1. Malaria

- This section provides an overview of:
  - Current WHO recommendations for the prevention and management of malaria related anaemia in pregnancy.
  - Interventions used for the prevention and management of prevention and management of malaria related anaemia identified in our cost-effectiveness review.
  - Mapping of effectiveness evidence for each intervention included in our cost-effectiveness review.
    - Different doses/coverage of IPTp-SP.
    - IPTp vs other interventions.
    - Community vs health-centre delivery of IPTp.
  1. Current WHO recommendations for the prevention of malaria in pregnancy
     - - In malaria-endemic areas, intermittent preventive treatment with sulfadoxine-pyrimethamine (IPTp-SP) is recommended for all pregnant women [24].
         - IPTp-SP should start as early as possible within the second trimester and not before week 13 of pregnancy due to risk of foetal malformation. [24]
         - At least three doses of IPTp-SP, each containing 500 mg/25 mg SP, should be received during pregnancy [24].
         - Alternatives to antenatal care (ANC) contact delivery of IPTp, such as community health worker delivery, may be explored where inequities in ANC services exist [24].
         - IPTp-SP should not be given to pregnant women receiving a sulfa-based medicine either therapeutically or prophylactically. This includes co-trimoxazole (trimethoprim–sulfamethoxazole) used in the setting of HIV [24].
         - Other contraindications for IPTp include women with severe acute illness, those unable to take oral medication, women who have received any drugs used for the purposes of IPTp within the last 30 days, or individuals allergic to any component of SP [24].
         - High doses of folic acid (≥ 5 mg daily) have been shown to reduce the effectiveness of SP as an antimalarial. Therefore, only low-dose formulations (e.g., 0.4 mg daily) should be co-administered with SP [24].
       - Where malaria transmission has decreased to below-endemic levels, the value of sustained use of IPTp-SP is unclear [24].
       - IPTp-SP should remain in use in areas with high SP resistance until more effective malaria chemoprevention alternatives are available [24].
  2. Current WHO recommendations for the treatment of malaria in pregnancy
     - - Pregnant women with uncomplicated P. falciparum malaria in their first trimester should be treated with artemether lumefantrine [24].
       - Pregnant women with uncomplicated P. falciparum malaria in their second and third trimesters should be treated with: [24]
         - artemether-lumefantrine (AL)
         - artesunate-amodiaquine (AS+AQ)
         - artesunate-mefloquine (ASMQ)
         - dihydroartemisinin-piperaquine (DHAP)
         - artesunate + sulfadoxine-pyrimethamine (AS+SP)
         - artesunate-pyronaridine (ASPY)
       - Uncomplicated malaria caused by P. vivax, P. ovale, P. malariae or P. knowlesi
         - In women who are pregnant or breastfeeding, weekly chemoprophylaxis with chloroquine can be given until delivery and breastfeeding are completed, then, as long as they are not G6PD deficient, primaquine can be given to prevent future relapse [24].
       - Treating severe malaria
         - Pregnant and lactating women should be treated with intravenous or intramuscular artesunate for at least 24 h and until they can tolerate oral medication. Once a patient has received at least 24 h of parenteral therapy and can tolerate oral therapy, treatment should be completed with 3 days of an ACT [24].
         - If artesunate is unavailable, intramuscular artemether should be given, and if this is unavailable then parenteral quinine should be started immediately until artesunate is obtained [24].
  3. Interventions used for the prevention and treatment of malaria identified in this review

| **Interventions compared** | **Included studies** | **Outcome(s) for which CE was calculated** |
| --- | --- | --- |
| Comparing different doses/coverage of IPTp-SP | | |
| Two doses of IPTp-SP Low coverage vs three doses of IPTp-SP Low coverage (HIV positive) | Choi et al., 2017 [25] | Per DALY averted:   - LBW, maternal malaria parasitaemia, maternal anaemia |
| Two doses of IPTp-SP Low coverage vs three doses of IPTp-SP High coverage (HIV positive) | Choi et al., 2017 [25] | Per DALY averted:   - LBW, maternal malaria parasitaemia, maternal anaemia |
| Two doses of IPTp-SP Low coverage vs CTX (HIV positive) | Choi et al., 2017 [25] | Per DALY averted:   - LBW, maternal malaria parasitaemia, maternal anaemia |
| Two doses of IPTp-SP vs three doses of IPTp-SP | Fernandes et al., 2015 [26] | Per DALY averted:   - LBW, moderate/severe maternal anaemia, maternal malaria parasitaemia |
| Baseline coverage of IPTp vs 95% coverage IPTp | Scott et al., 2020 [27] | Per case of anaemia averted |
| IPTp vs other interventions | | |
| IPTp-SP vs ISTp-AL | Fernandes et al., 2016 [28] | Per DALY averted:   - LBW, moderate/severe anaemia, clinical malaria |
| Three doses of IPTp-SP vs three doses of IPTp-DP | Fernandes et al., 2020 [29] | Per DALY averted:   - Stillbirths, neonatal mortality, LBW, mild anaemia, moderate anaemia, clinical malaria |
| Monthly doses of IPTp-SP vs monthly doses of IPTp-DP | Fernandes et al., 2020 [29] | Per DALY averted:   - Stillbirths, neonatal mortality, LBW, mild anaemia, moderate anaemia, clinical malaria |
| Two doses of IPTp-SP vs insecticide treated bed nets (ITNs) | Hansen et al., 2012 [30] | Per DALY averted:   - Maternal anaemia, LBW |
| Two doses of IPTp-SP vs ITNs and IPTp-SP | Hansen et al., 2012 [30] | Per DALY averted:   - Maternal anaemia, LBW |
| SST-DP vs monthly IPTp-DP | Paintain et al., 2020 [31] | Per DALY averted:   - Fetal loss/infant death, LBW, moderate/severe anaemia, clinical malaria |
| Three doses of IPTp-SP vs two doses of IPTp-MQ (HIV negative) | Sicuri et al., 2015 [32] | Per DALY averted:   - Non-obstetric hospital admissions |
| Daily CTX, ITNs and three doses of IPTp-MQ vs daily CTX, ITNs and IPTp-placebo (HIV positive) | Sicuri et al., 2015 [32] | Per DALY averted:   - Clinical malaria, anaemia at delivery, non-obstetric hospital admissions |
| Community vs health-centre delivery of IPTp | | |
| Combined C-IPTp + H-IPTp vs H-IPTp | Cirera et al., 2023 [33] | Per DALY averted:   - Maternal anaemia, maternal malaria parasitaemia, LBW, neonatal mortality |
| C-IPTp vs H-IPTp | Mbonye et al., 2008 [34] | Per DALY averted:   - Maternal anaemia, maternal malaria parasitaemia, LBW |

- 1. Effectiveness evidence of different doses of IPTp-SP
  - A 2013 systematic review [35] of seven trials compared two doses of IPTp-SP to ≥Three doses IPTp-SP for which two studies [25, 26] calculated cost-effectiveness

Table 2.4A: Effectiveness evidence for 2 dose IPTp-SP vs zero or one doses of IPTp-SP relating to LBW: used by one study [25] as a component of a DALY

| **Outcome** | **Statistical method** | **Effect size** | **Source** |
| --- | --- | --- | --- |
| Summary   - No systematic review comparing these interventions was identified. | | | |

Table 2.4B: Effectiveness evidence for two doses of IPTp-SP vs zero or one doses of IPTp-SP relating to maternal malaria parasitaemia: used by one study [25] as a component of a DALY

| **Outcome** | **Statistical method** | **Effect size** | **Source** |
| --- | --- | --- | --- |
| Summary   - No systematic review comparing these interventions was identified. | | | |

Table 2.4C: Effectiveness evidence for two doses of IPTp-SP vs zero or one doses of IPTp-SP relating to maternal anaemia: used by one study [25] as a component of a DALY

| **Outcome** | **Statistical method** | **Effect size** | **Source** |
| --- | --- | --- | --- |
| Summary   - No systematic review comparing these interventions was identified. | | | |

Table 2.4D: Effectiveness evidence for three doses of IPTp-SP vs two doses of IPTp-SP relating to LBW: used by two studies [25, 26] as a component of a DALY

| **Outcome** | **Statistical method** | **Effect size** | **Source** |
| --- | --- | --- | --- |
| LBW | RR (95% CI) | 0.80 (0.69, 0.94) | Kayentao et al., 2013 [35] |
| LBW (HIV positive) | RR (95% CI) | 0.86 (0.53, 1.39) | Kayentao et al., 2013 [35] |
| Summary   - In one review, three doses of IPTp-SP reduced the risk of LBW compared to two doses of IPTp-SP. | | | |

RR: Relative risk

Table 2.4E: Effectiveness evidence for three doses of IPTp-SP vs two doses of IPTp-SP relating to maternal malaria parasitaemia: used by two studies [25, 26] as a component of a DALY

| **Outcome** | **Statistical method** | **Effect size** | **Source** |
| --- | --- | --- | --- |
| Maternal parasitaemia | RR (95% CI) | 0.68 (0.52, 0.89) | Kayentao et al., 2013 [35] |
| Maternal parasitaemia (HIV positive) | RR (95% CI) | 0.26 (0.15, 0.46) | Kayentao et al., 2013 [35] |
| Summary   - In one review, three doses of IPTp-SP reduced the risk of maternal malaria parasitaemia compared to two doses of IPTp-SP. | | | |

RR: Relative risk

Table 2.4F: Effectiveness evidence for three doses of IPTp-SP vs two doses of IPTp-SP relating to maternal anaemia: used by one study [25] as a component of a DALY

| **Outcome** | **Statistical method** | **Effect size** | **Source** |
| --- | --- | --- | --- |
| Maternal anaemia | RR (95% CI) | 0.95 (0.90, 1.01) | Kayentao et al., 2013 [35] |
| Maternal anaemia (HIV positive) | RR (95% CI) | 0.96 (0.87, 1.07) | Kayentao et al., 2013 [35] |
| Summary   - In one review, three doses of IPTp-SP did not significantly reduce the risk of maternal anaemia compared to two doses of IPTp-SP. | | | |

RR: Relative risk

Table 2.4G: Effectiveness evidence for three doses of IPTp-SP vs two doses of IPTp-SP relating to moderate/severe maternal anaemia: used by one study[26] as a component of a DALY

| **Outcome** | **Statistical method** | **Effect size** | **Source** |
| --- | --- | --- | --- |
| Moderate/severe maternal anaemia all gravidae women | RR (95% CI) | 0.73 (0.48, 1.11) | Kayentao et al., 2013 [35] |
| Moderate/severe maternal anaemia all gravidae women (HIV positive) | RR (95% CI) | 0.60 (0.06, 5.85) | Kayentao et al., 2013 [35] |
| Moderate/severe maternal anaemia all G1-G2 women | RR (95% CI) | 0.60 (0.36, 0.99) | Kayentao et al., 2013 [35] |
| Moderate/severe maternal anaemia all G1-G2 women (HIV positive) | RR (95% CI) | 0.26 (0.06, 1.23) | Kayentao et al., 2013 [35] |
| Summary   - In one review, three doses of IPTp-SP reduced the risk of moderate/severe maternal anaemia in G1-G2 women however not in women of all gravidae or with HIV compared to two doses of IPTp-SP. | | | |

RR: Relative risk

Table 2.4H: Effectiveness evidence for two doses of IPTp-SP vs placebo relating to neonatal mortality: used by one study [33] as a component of a DALY

| **Outcome** | **Statistical method** | **Effect size** | **Source** |
| --- | --- | --- | --- |
| Summary   - No systematic review comparing these interventions was identified. | | | |

- 1. IPTp vs other interventions
- A 2015 systematic review [36] of 19 studies compared CTX to IPTp in pregnant women for which cost-effectiveness was calculated by one included study [25], however did not specify against which dose or drug used as IPTp effectiveness was determined.
- A 2021 systematic review [37] of five studies compared ISTp using artemisinin-combination therapies to IPTp-SP for which cost-effectiveness was calculated by one included study [28].
- A 2021 systematic review [38] of three studies compared IPTp-DP and IPTp-SP for which cost-effectiveness was calculated by one included study [29].
- One 2024 Cochrane review [39] of 14 RCTs compared IPTp to CTX for which cost-effectiveness was calculated in one study [32].
- One 2018 Cochrane review [40] of six RCTs compared IPTp-MQ to IPTp-SP and CTX for which cost-effectiveness was calculated in one study [32].

Table 2.5A: Effectiveness evidence for CTX vs two doses of IPTp-SP relating to LBW: used by one study [25] as a component of a DALY

| **Outcome** | **Statistical method** | **Effect size** | **Source** |
| --- | --- | --- | --- |
| Summary   - No systematic review comparing these interventions was identified. | | | |

Table 2.5B: Effectiveness evidence for CTX vs two doses of IPTp-SP relating to maternal malaria parasitaemia: used by one study [25] as a component of a DALY

| **Outcome** | **Statistical method** | **Effect size** | **Source** |
| --- | --- | --- | --- |
| Summary   - No systematic review comparing these interventions was identified. | | | |

Table 2.5C: Effectiveness evidence for CTX vs two doses of IPTp-SP relating to maternal anaemia: used by one study [25] as a component of a DALY

| **Outcome** | **Statistical method** | **Effect size** | **Source** |
| --- | --- | --- | --- |
| Summary   - No systematic review comparing these interventions was identified. | | | |

Table 2.5D: Effectiveness evidence for ISTp-AL vs IPTp-SP relating to: LBW used by one study [28] as a component of a DALY

| **Outcome** | **Statistical method** | **Effect size** | **Source** |
| --- | --- | --- | --- |
| LBW | RR (95% CI) | 1.08 (0.97, 1.20) | Gutman et al., 2021 [37] |
| Summary   - In one review, there was no difference in neonatal mortality between oral and intravenous iron supplements. | | | |

RR: Relative risk

Table 2.5E: Effectiveness evidence for ISTp-AL vs IPTp-SP relating to: moderate/severe maternal anaemia used by one study [28] as a component of a DALY

| **Outcome** | **Statistical method** | **Effect size** | **Source** |
| --- | --- | --- | --- |
| Moderate/severe anaemia | RR (95% CI) | 1.15 (0.96, 1.38) | Gutman et al., 2021 [37] |
| Summary   - In one review, there was no difference in neonatal mortality between oral and intravenous iron supplements. | | | |

RR: Relative risk

Table 2.5F: Effectiveness evidence for ISTp-AL vs IPTp-SP relating to: clinical malaria cases used by one study [28] as a component of a DALY

| **Outcome** | **Statistical method** | **Effect size** | **Source** |
| --- | --- | --- | --- |
| Clinical malaria | RR (95% CI) | 1.21 (1.04, 1.42) | Gutman et al., 2021 [37] |
| Summary   - In one review, there was no difference in neonatal mortality between oral and intravenous iron supplements. | | | |

RR: Relative risk

Table 2.5G: Effectiveness evidence for three doses of IPTp-DP vs three doses of IPTp-SP relating to: stillbirths used by one study [29] as a component of a DALY

| **Outcome** | **Statistical method** | **Effect size** | **Source** |
| --- | --- | --- | --- |
| Summary   - No effectiveness data for this outcome was available in relevant systematic reviews.. | | | |

Table 2.5H: Effectiveness evidence for three doses of IPTp-DP vs three doses of IPTp-SP relating to: neonatal mortality used by one study [29] as a component of a DALY

| **Outcome** | **Statistical method** | **Effect size** | **Source** |
| --- | --- | --- | --- |
| Summary   - No effectiveness data for this outcome was available in relevant systematic reviews. | | | |

Table 2.5I: Effectiveness evidence for three doses of IPTp-DP vs three doses of IPTp-SP relating to: LBW used by one study [29] as a component of a DALY

| **Outcome** | **Statistical method** | **Effect size** | **Source** |
| --- | --- | --- | --- |
| LBW | OR (95% CI) | 1.20 (0.73, 1.97) | Olaleye et al., 2021 [38] |
| Summary   - One review found that three doses of IPTp-DP reduced the risk of LBW compared to three doses of IPTp-SP. | | | |

OR: Odds ratio

Table 2.5J: Effectiveness evidence for three doses of IPTp-DP vs three doses of IPTp-SP relating to: mild maternal anaemia used by one study [29] as a component of a DALY

| **Outcome** | **Statistical method** | **Effect size** | **Source** |
| --- | --- | --- | --- |
| Summary   - No effectiveness data for this outcome was available in relevant systematic reviews. | | | |

Table 2.5K: Effectiveness evidence for three doses of IPTp-DP vs three doses of IPTp-SP relating to: moderate maternal anaemia used by one study [29] as a component of a DALY

| **Outcome** | **Statistical method** | **Effect size** | **Source** |
| --- | --- | --- | --- |
| Summary   - No effectiveness data for this outcome was available in relevant systematic reviews. | | | |

Table 2.5L: Effectiveness evidence for three doses of IPTp-DP vs three doses of IPTp-SP relating to: clinical malaria cases used one study [29] as a component of a DALY

| **Outcome** | **Statistical method** | **Effect size** | **Source** |
| --- | --- | --- | --- |
| Clinical malaria | OR (95% CI) | 0.17 (0.10, 0.29) | Olaleye et al., 2021 [38] |
| Summary   - In one review, three doses of IPTp-DP significantly reduced clinical malaria compared to three doses of IPTp-SP. | | | |

OR: Odds ratio

Table 2.5M: Effectiveness evidence for monthly IPTp-DP vs monthly IPTp-SP relating to: stillbirths used one study [29] as a component of a DALY

| **Outcome** | **Statistical method** | **Effect size** | **Source** |
| --- | --- | --- | --- |
| Summary   - No effectiveness data for this outcome was available in relevant systematic reviews. | | | |

Table 2.5N: Effectiveness evidence for monthly IPTp-DP vs monthly IPTp-SP relating to: neonatal mortality used by one study [29] as a component of a DALY

| **Outcome** | **Statistical method** | **Effect size** | **Source** |
| --- | --- | --- | --- |
| Summary   - No effectiveness data for this outcome was available in relevant systematic reviews. | | | |

Table 2.5O: Effectiveness evidence for monthly IPTp-DP vs monthly IPTp-SP relating to: LBW used by one study [29] as a component of a DALY

| **Outcome** | **Statistical method** | **Effect size** | **Source** |
| --- | --- | --- | --- |
| Summary   - No effectiveness data for this outcome was available in relevant systematic reviews. | | | |

Table 2.5P: Effectiveness evidence for monthly IPTp-DP vs monthly IPTp-SP relating to: moderate maternal anaemia used by one study [29] as a component of a DALY

| **Outcome** | **Statistical method** | **Effect size** | **Source** |
| --- | --- | --- | --- |
| Summary   - No effectiveness data for this outcome was available in relevant systematic reviews. | | | |

Table 2.5Q: Effectiveness evidence for monthly IPTp-DP vs monthly IPTp-SP relating to: clinical malaria cases used by one study [29] as a component of a DALY

| **Outcome** | **Statistical method** | **Effect size** | **Source** |
| --- | --- | --- | --- |
| Summary   - No effectiveness data for this outcome was available in relevant systematic reviews. | | | |

Table 2.5R: Effectiveness evidence for ITNs two doses of IPTp-SP relating to: maternal anaemia used by one study [30] as a component of a DALY

| **Outcome** | **Statistical method** | **Effect size** | **Source** |
| --- | --- | --- | --- |
| Summary   - No systematic review comparing these interventions was identified. | | | |

Table 2.5S: Effectiveness evidence for ITNs vs two doses of IPTp-SP relating to: LBW used by one study [30] as a component of a DALY

| **Outcome** | **Statistical method** | **Effect size** | **Source** |
| --- | --- | --- | --- |
| Summary   - No systematic review comparing these interventions was identified. | | | |

Table 2.5T: Effectiveness evidence for two doses of IPTp-SP and ITNs vs two doses of IPTp-SP relating to: maternal anaemia used by one study [30] as a component of a DALY

| **Outcome** | **Statistical method** | **Effect size** | **Source** |
| --- | --- | --- | --- |
| Summary   - No systematic review comparing these interventions was identified. | | | |

Table 2.5U: Effectiveness evidence for two doses of IPTp-SP vs two doses of IPTp-SP and ITNs relating to: LBW used by one study [30] as a component of a DALY

| **Outcome** | **Statistical method** | **Effect size** | **Source** |
| --- | --- | --- | --- |
| Summary   - No systematic review comparing these interventions was identified. | | | |

Table 2.5V: Effectiveness evidence for SST-DP vs monthly IPTp-DP relating to: fetal loss/infant death used by one study [31] as a component of a DALY

| **Outcome** | **Statistical method** | **Effect size** | **Source** |
| --- | --- | --- | --- |
| Summary   - No systematic review comparing these interventions was identified. | | | |

Table 2.5X: Effectiveness evidence for SST-DP vs monthly IPTp-DP relating to: LBW used by one study [31] as a component of a DALY

| **Outcome** | **Statistical method** | **Effect size** | **Source** |
| --- | --- | --- | --- |
| Summary   - No systematic review comparing these interventions was identified. | | | |

Table 2.5W: Effectiveness evidence for SST-DP vs monthly IPTp-DP relating to: moderate/severe maternal anaemia used by one study [31] as a component of a DALY

| **Outcome** | **Statistical method** | **Effect size** | **Source** |
| --- | --- | --- | --- |
| Summary   - No systematic review comparing these interventions was identified. | | | |

Table 2.5Y: Effectiveness evidence for SST-DP vs monthly IPTp-DP relating to: clinical malaria cases used by one study [31] as a component of a DALY

| **Outcome** | **Statistical method** | **Effect size** | **Source** |
| --- | --- | --- | --- |
| Summary   - No systematic review comparing these interventions was identified. | | | |

Table 2.5Z: Effectiveness evidence for two doses of IPTp-MQ vs three doses of IPTp-SP relating to: clinical malaria cases used by one study [32] as a component of a DALY

| **Outcome** | **Statistical method** | **Effect size** | **Source** |
| --- | --- | --- | --- |
| Clinical malaria episodes during pregnancy | IRR (95% CI) | 0.83 (0.65, 1.05) | González et al., 2018 [40] |
| Summary   - In one review, IPTp-MQ reduced clinical malaria cases compared to IPTp-SP. | | | |

IRR= incidence rate ratio

Table 2.5AA: Effectiveness evidence for Daily CTX, ITNs and three doses of IPTp-MQ vs daily CTX, ITNs and IPTp-placebo relating to: maternal malaria parasitaemia used by one study [25] as a component of DALY

| **Outcome** | **Statistical method** | **Effect size** | **Source** |
| --- | --- | --- | --- |
| Summary   - No systematic review comparing these interventions was identified. | | | |

Table 2.5AB: Effectiveness evidence for two doses of IPTp-MQ vs three doses of IPTp-SP relating to: anaemia at delivery used by one study [32] as a component of a DALY

| **Outcome** | **Statistical method** | **Effect size** | **Source** |
| --- | --- | --- | --- |
| Maternal anaemia at delivery | RR (95% CI) | 0.84 (0.76, 0.94) | González et al., 2018 [40] |
| Summary   - In one review, IPTp-MQ reduced maternal anaemia at delivery compared to IPTp-SP. | | | |

RR: Relative risk

Table 2.5AC: Effectiveness evidence for three doses of IPTp-MQ vs IPTp-placebo relating to: non-obstetric hospital admissions used by one study [32] as a component of a DALY

| **Outcome** | **Statistical method** | **Effect size** | **Source** |
| --- | --- | --- | --- |
| Summary   - No effectiveness data for this outcome was available in relevant systematic reviews. | | | |

- 1. Community vs health-centre delivery of IPTp
  - One 2024 systematic review of 23 studies [41] assessed the effectiveness of C-IPTp for which cost-effectiveness was determined in two included studies [33, 34].

Table 2.6A: Effectiveness evidence for C-IPTp vs H-IPTp relating to increases in the coverage of three doses of IPTp-SP: used by one study [33] as a component of a DALY

| **Outcome** | **Statistical method** | **Effect size** | **Source** |
| --- | --- | --- | --- |
| Proportion of women receiving 3 or more doses of IPTp-SP | RR (95% CI) | 1.73 (1.19, 2.50) | Koita et al., 2024 [41] |
| Summary   - No effectiveness data for this outcome was available in relevant systematic reviews. | | | |

RR: Relative risk

Table 2.6B: Effectiveness evidence for C-IPTp vs H-IPTp relating to maternal anaemia: used one study [34] as a component of a DALY

| **Outcome** | **Statistical method** | **Effect size** | **Source** |
| --- | --- | --- | --- |
| Summary   - No effectiveness data for this outcome was available in relevant systematic reviews. | | | |

Table 2.6C: Effectiveness evidence for C-IPTp vs H-IPTp relating to maternal malaria parasitaemia: used one study [34] as a component of a DALY

| **Outcome** | **Statistical method** | **Effect size** | **Source** |
| --- | --- | --- | --- |
| Summary   - No effectiveness data for this outcome was available in relevant systematic reviews. | | | |

Table 2.6D: Effectiveness evidence for C-IPTp vs H-IPTp relating to LBW: used by used one study [34] as a component of a DALY

| **Outcome** | **Statistical method** | **Effect size** | **Source** |
| --- | --- | --- | --- |
| Summary   - No effectiveness data for this outcome was available in relevant systematic reviews. | | | |

- 1. Comparison of effectiveness data for interventions used for the prevention and management of malaria-related anaemia in pregnancy reported in the literature and the studies included in this review (Table 2.7).

| Included study (Intervention/s & Comparator/s) | Outcome(s) used to calculate CE | Model parameters/outcome probabilities/effectiveness evidence cited in each study | Source of effectiveness evidence cited in each study | Corresponding effectiveness evidence from recent systematic reviews identified in this report | Comparison between effectiveness evidence from studies included in our cost-effectiveness review with recent effectiveness systematic reviews | Comments on similarities/discrepancies with effectiveness evidence from recent systematic reviews |
| --- | --- | --- | --- | --- | --- | --- |
| Comparing different doses/coverage of IPTp-SP | | | | | | |
| Choi et al., 2017 [25]   1. 2-IPT Low (HIV positive) 2. 3-IPT Low   (HIV positive)   1. 3-IPT High   (HIV positive)   1. CTX   (HIV positive) | DALYs averted | | | | | |
|  | RR (95% CI) of LBW | Two doses of IPTp-SP vs zero or one doses of IPTp-SP: 3.25 (95% CI not stated) | Gutman et al., 2013 [42] | Table 2.4A | No systematic review comparing these interventions was identified. | N/A |
|  |  | Three doses of IPTp-SP vs two doses of IPTp-SP: 0.86 (0.53, 1.39) | Kayentao et al., 2013 [35] | Table 2.4D | The RR used in this study is the RR described by Kayentao et al., 2013 [35]. | The effectiveness described by the study is **congruent** with current evidence. |
|  |  | CTX vs two doses of IPTp-SP: 1.1 (0.86, 1.34) | Suthar et al., 2015 [43], Klement et al., 2014 [44] | Table 2.5A | No systematic review comparing these interventions was identified. | N/A |
|  | RR (95% CI) of maternal malaria parasitaemia | Two doses of IPTp-SP vs zero or one doses of IPTp-SP: 1.4 (95% CI not stated) | Kapito-Tembo et al., 2011 [45] | Table 2.4B | No systematic review comparing these interventions was identified. | N/A |
|  |  | Three doses of IPTp-SP vs two doses of IPTp-SP: 0.26 (0.15, 0.46) | Kayentao et al., 2013 [35] | Table 2.4E | The RR used in this study is the RR described by Kayentao et al., 2013 [35]. | The effectiveness described by the study is **congruent** with current evidence. |
|  |  | CTX vs two doses of IPTp-SP: 0.43 (0.19, 1.10) | Suthar et al., 2015 [43], Klement et al., 2014 [44] | Table 2.5B | No systematic review comparing these interventions was identified. | N/A |
|  |  | CTX+ART vs CTX: 0.38 (95% CI not stated) | Mermin et al., 2006 [46] | Table 2.7H | No systematic review comparing these interventions was identified. | N/A |
|  | RR (95% CI) of maternal anaemia | Two doses of IPTp-SP vs of zero or one doses of IPTp-SP: 1.03 (95% CI not stated) | Gutman et al., 2013 [42] | Table 2.4C | No systematic review comparing these interventions was identified. | N/A |
|  |  | Three doses of IPTp-SP vs two doses of IPTp-SP: 0.96 (0.87, 1.07) | Kayentao et al., 2013 [35] | Table 2.4F | The RR used in this study is the RR described by Kayentao et al., 2013 [35]. | The effectiveness described by the study is **congruent** with current evidence. |
|  |  | CTX vs two doses of IPTp-SP 0.72 (0.61, 0.83) | Kapito-Tembo et al., 2011 [45] | Table 2.5C | No systematic review comparing these interventions was identified. | N/A |
| Fernandes et al., 2015 [26]   1. Two doses of IPTp-SP 2. ≥Three doses of IPTp-SP | DALYs averted | | | | | |
|  | RR (95%CI) of LBW | G1/G2: 0.80 (0.68, 0.95)  G3+: 0.79 (0.49, 1.27) | Kayentao et al., 2013 [35] | Table 2.4D | The RR used in this study is the RR described by Kayentao et al., 2013 [35]. | The effectiveness described by the study is **congruent** with current evidence. |
|  | RR (95%CI) of moderate/severe maternal anaemia | G1/G2: 0.60 (0.36, 0.99)  G3+: 1.18 (0.56, 2.48) | Kayentao et al., 2013 [35] | Table 2.4G | The RR used in this study is the RR described by Kayentao et al., 2013 [35]. | The effectiveness described by the study is **congruent** with current evidence. |
|  | RR (95%CI) of maternal malaria parasitaemia | G1/G2: 0.54 (0.37, 0.80)  G3+: 0.97 (0.75, 1.24) | Kayentao et al., 2013 [35] | Table 2.4E | The RR used in this study is the RR described by Kayentao et al., 2013 [35]. | The effectiveness described by the study is **congruent** with current evidence. |
| Scott et al., 2020 [27]   1. Baseline coverage of IPTp 2. 95% coverage coverage of IPTp | RR (95%CI) of anaemia averted | 0.83 (0.74,0.93) | Radeva-Petrova et al., 2014 [47] | N/A | The comparator (baseline coverage of IPTp) was unspecified and therefore was not able to be compared to the literature. | N/A |
| IPTp vs other interventions | | | | | | |
| Fernandes et al., 2016 [28]   1. IPTp-SP 2. ISTp-AL | DALYs averted | | | | | |
|  | Risk of LBW per 1000 women (95% CI) | IPTp-SP: 151 (137, 167)  ISTP-AL: 156 (141, 172)  Calculated RR: 1.03 | Tagbor et al., 2015 [48] | Table 2.5D | The calculated RR for this outcome is **almost identical** to the RR reported by Gutman et al., 2021 [37] (1.08) and falls **within** the 95%CI (0.97, 1.20). | The effectiveness described by the study is **congruent** with current evidence. |
|  | Cases of moderate/severe anaemia per 1000 women (95% CI) | IPTp-SP: 17 (12, 25)  ISTp-AL: 23 (17, 32)  Calculated RR: 1.35 | Tagbor et al., 2015 [48] | Table 2.5E | The calculated RR for this outcome is **similar** to the RR of reported by Gutman et al., 2021 [37] (1.15) and falls **within** the 95%CI (0.96, 1.38). | The effectiveness described by the study is **congruent** with current evidence. |
|  | Episodes of clinical malaria at unscheduled visits (95% CI) | IPTp-SP: 74 (61, 92)  ISTp-AL: 115 (96, 144)  Calculated RR: 1.55 | Tagbor et al., 2015 [48] | Table 2.5F | The calculated RR for this outcome is **higher** than the RR reported by Gutman et al., 2021 [37] (1.21) and falls **outside** of the 95%CI (1.04, 1.42). | The effectiveness of ISTp-AL in reducing episodes of clinical malaria has been **overestimated** compared to most recent systematic reviews.  This may have resulted in ISTp-AL appearing **more cost-effective.** |
| Fernandes et al., 2020 [29]   1. Three doses of IPTp-SP 2. Three doses of IPTp-DP 3. Monthly IPTp-SP 4. Monthly IPTp-DP | DALYs averted | | | | | |
|  | Number of stillbirths | Monthly IPTp-DP: 2/341  Monthly IPTp-SP: 5/339 | Kajubi et al., 2019 [49] | Table 2.5M | No effectiveness data for this outcome was available in relevant systematic reviews. | N/A |
|  |  | Three doses of IPTp-DP: 5/541  Three doses of IPTp-SP: 17/552  RR (95%CI): 0.30 (0.11, 0.80) | Study meta-analysis | Table 2.5G | No effectiveness data for this outcome was available in relevant systematic reviews. | N/A |
|  | Number of neonatal deaths | Monthly IPTp-DP: 4/341  Monthly IPTp-SP: 6/335 | Kajubi et al., 2019 [49] | Table 2.5N | No effectiveness data for this outcome was available in relevant systematic reviews. | N/A |
|  |  | Three doses of IPTp-DP: 6/536  Three doses of IPTp-SP: 12/535  RR (95%CI): 0.52 (0.21, 1.32) | Study meta-analysis | Table 2.5H | No effectiveness data for this outcome was available in relevant systematic reviews. | N/A |
|  | Cases of LBW | Monthly IPTp-DP: 24/361  Monthly IPTp-SP: 29/358 | Kajubi et al., 2019 [49] | Table 2.5O | No effectiveness data for this outcome was available in relevant systematic reviews. | N/A |
|  |  | Three doses of IPTp-DP: 33/500  Three doses of IPTp-SP: 26/493  RR (95%CI): 1.32 (0.80, 2.17)  Calculated OR: 1.25 | Study meta-analysis | Table 2.5I | The calculated OR for this outcome is **very similar** to the OR described by Olaleye et al., 2021 [38] (1.20) and falls **within** the 95%CI (0.73, 1.97). | The effectiveness described by the study is **congruent** with current evidence. |
|  | Cases of mild anaemia | Monthly IPTp-DP: not recorded  Monthly IPTp-SP: not recorded | Kajubi et al., 2019 [49] | N/A | Cases of mild anaemia for this intervention were not described in the included study. | N/A |
|  |  | Three doses of IPTp-DP: 214/460  Three doses of IPTp-SP: 209/473  RR: 1.05 (0.91, 1.21) | Study meta-analysis | Table 2.5J | No effectiveness data for this outcome was available in relevant systematic reviews. | N/A |
|  | Cases of moderate anaemia | Monthly IPTp-DP: 89/893  Monthly IPTp-SP: 171/870 | Kajubi et al., 2019 [49] | Table 2.5P | No effectiveness data for this outcome was available in relevant systematic reviews. | N/A |
|  |  | Three doses of IPTp-DP: 22/460  Three doses of IPTp-SP: 39/473  RR (95%CI): 0.58 (0.35, 0.96) | Study meta-analysis | Table 2.5K | No effectiveness data for this outcome was available in relevant systematic reviews. | N/A |
|  | Cases of clinical malaria | Monthly IPTp-DP: 3/349  Monthly IPTp-SP: 75/338 | Kajubi et al., 2019 [49] | Table 2.5Q | No effectiveness data for this outcome was available in relevant systematic reviews. | N/A |
|  |  | Three doses of IPTp-DP: 21/579    Three doses of IPTp-SP: 83/583  RR (95%CI): 0.23 (0.15, 0.36)  Calculated OR: 0.25 | Study meta-analysis | Table 2.5L | The calculated OR for this outcome is **similar** to the OR of described by Olaleye et al., 2021 [38] (0.17) and falls **within** the 95%CI (0.10, 0.29). | The effectiveness described by the study is **congruent** with current evidence. |
| Hansen et al., 2012 [30]   1. Two doses of IPTp-SP 2. ITNs 3. Two doses of IPTp-SP and ITNs | DALYs averted | | | | | |
|  | Cases of maternal anaemia per 1000 pregnancies | Two doses of IPTp-SP: 142  ITN: 163  Two doses of IPTp-SP + ITN: 136 | Ndyomugyenyi et al., 2011 [50] | Tables 2.5R, 2.5T | No systematic review comparing these interventions was identified. | N/A |
|  | Cases of LBW per 1000 pregnancies | Two doses of IPTp-SP: 65  ITN: 63  Two doses of IPTp-SP + ITN: 69 | Ndyomugyenyi et al., 2011 [50] | Tables 2.5S, 2.5U | No systematic review comparing these interventions was identified. | N/A |
| Paintain et al., 2020 [31]   1. SST-DP 2. Monthly IPTp-DP | DALYs averted | | | | | |
|  | Cases of fetal loss or infant deaths per 1000 women (number of events) | SST: 35.1 13/370  IPTP: 33.0 10/303 | Ahmed et al., 2019 [51] | Table 2.5V | No systematic review comparing these interventions was identified. | N/A |
|  | Cases of LBW per 1000 women (number of events) | SST-DP: 129.8 44/339  IPTp: 115.2 31/269 | Ahmed et al., 2019 [51] | Table 2.5W | No systematic review comparing these interventions was identified. | N/A |
|  | Cases of moderate/severe anaemia per 1000 women (number of events) | SST: 152.0 52/342  IPTp: 96.3 26/270 | Ahmed et al., 2019 [51] | Table 2.5X | No systematic review comparing these interventions was identified. | N/A |
|  | Cases of clinical malaria per 1000 women (number of events) | SST: 36.5 (13/356)  IPTp: 6.3 (2/319) | Ahmed et al., 2019 [51] | Table 2.5Y | No systematic review comparing these interventions was identified. | N/A |
| Sicuri et al., 2015 [32]   1. Three doses of IPTp-SP (HIV negative) 2. Two doses of IPTp-MQ (HIV negative) 3. Daily CTX, ITNs and IPTp-placebo (HIV positive) 4. Daily CTX, ITNs and three doses of IPTp-MQ (HIV positive) | DALYs averted | | | | | |
|  | RR (95%CI) of clinical malaria | Two doses of MQ vs three doses of SP (HIV negative): 0.67 (0.52, 0.88) | González et al., 2014 [52] | Table 2.5AA | The RR used in this study is **similar** to the RR described by Gonzalez et al., 2018 [40] (0.83) and falls within the 95% CI (0.65, 1.05). | The effectiveness described by the study is **congruent** with current evidence. |
|  | RR (95%CI) of anaemia at delivery | Two doses of MQ vs three doses of SP (HIV negative): 0.92 (0.85, 0.99) | González et al., 2014[52] | Table 2.5AB | The RR used in this study is **similar** to the RR described by Gonzalez et al., 2018 [40] (0.84) and falls within the 95% CI (0.76, 0.94). | The effectiveness described by the study is **congruent** with current evidence. |
|  | RR (95%CI) of non-obstetric hospital admissions | Daily CTX, ITNs and three doses of IPTp-MQ vs daily CTX, ITNs and IPTp-placebo (HIV positive): 0.59 (0.37, 0.95) | González et al., 2014 [53] | Table 2.5AC | No systematic review comparing these interventions was identified. | N/A |
| Community vs health-centre delivery of IPTp | | | | | | |
| Cirera et al., 2023 [33]   1. H-IPTp 2. Combined C-IPTp + H-IPTp | Impact of C-IPTp and H-IPTp on the coverage of three doses of IPTp-SP | | | | | |
|  | Average increase in the coverage of three doses of IPTp-SP across 3 sites in the Democratic Republic of the Congo (DRC) | 208% | Household surveys conducted within the study | Table 2.6A | The increase in coverage of three doses of IPTp-SP described by this study is **higher** than the increase reported by Koita et al., 2024 [41] (73%) described by and falls **outside** of the 95% CI (19%, 150%). | The effectiveness of combined C-IPTp + H-IPTp in increasing the coverage of three doses of IPTp-SP in the DRC may have been **overestimated** in this study.    This may have resulted in combined C-IPTp + H-IPTp appearing **more cost-effective.** |
|  | Average increase in the coverage of three doses of IPTp-SP across 3 sites in Madagascar | 169% | Household surveys conducted within the study | Table 2.6A | The increase in coverage of three doses of IPTp-SP described by this study is **higher** than the increase reported by Koita et al., 2024 [41] (73%) described by and falls **outside** of the 95% CI (19%, 150%). | The effectiveness of combined C-IPTp + H-IPTp in increasing the coverage of three doses of IPTp-SP in Madagascar may have been **overestimated** in this study.    This may have resulted in combined C-IPTp + H-IPTp appearing **more cost-effective.** |
|  | Average increase in the coverage of three doses of IPTp-SP across 3 sites in Mozambique | 11% | Household surveys conducted within the study | Table 2.6A | The increase in coverage of three doses of IPTp-SP described by this study is **lower** than the increase reported by Koita et al., 2024 [41] (73%) described by and falls **outside** of the 95% CI (19%, 150%). | The effectiveness of combined C-IPTp + H-IPTp in increasing the coverage of three doses of IPTp-SP in Mozambique may have been **underestimated** in this study.    This may have resulted in combined C-IPTp + H-IPTp appearing **less cost-effective.** |
|  | Average increase in the coverage of three doses of IPTp-SP across 3 sites in Nigeria | 448% | Household surveys conducted within the study | Table 2.6A | The increase in coverage of three doses of IPTp-SP described by this study is **higher** than the increase reported by Koita et al., 2024 [41] (73%) described by and falls **outside** of the 95% CI (19%, 150%). | The effectiveness of combined C-IPTp + H-IPTp in increasing the coverage of three doses of IPTp-SP in Nigeria may have been **overestimated** in this study.    This may have resulted in combined C-IPTp + H-IPTp appearing **more cost-effective.** |
|  | DALYs averted | | | | | |
|  | Protective efficacy of three doses of IPTp-SP in reducing maternal anaemia | Modelled using data comparing 2 or more doses of IPTp-SP to placebo: 8% | Menéndez et al., 2008 [54] | Table 2.4C | No systematic review comparing these interventions was identified. | N/A |
|  | Protective efficacy of three doses of IPTp-SP in reducing maternal malaria parasitaemia | Modelled using data comparing 2 or more doses of IPTp-SP to placebo: 40% | Menéndez et al., 2008 [54] | Table 2.4B | No systematic review comparing these interventions was identified. | N/A |
|  | Protective efficacy of three doses of IPTp-SP in reducing LBW | Modelled using data comparing 3 or more doses of IPTp-SP to two doses of IPTp-SP: 21% | Kayentao et al., 2013 [35], Eisele et al., 2012 [55] | Table 2.4D | The RR used in this study is almost **identical** to the RR described by Kayentao et al., 2013 [35] (0.80) and falls **within** the 95% CI (0.69, 0.94). | The effectiveness described by the study is **congruent** with current evidence. |
|  | Protective efficacy of three doses of IPTp-SP in reducing neonatal mortality | Modelled using data comparing 2 or more doses of IPTp-SP to placebo: 18% | Eisele et al., 2012 [55] | Table 2.4H | No systematic review comparing these interventions was identified. | N/A |
| Mbonye et al., 2008 [34]   1. H-IPTp 2. C-IPTp | DALYs averted | | | | | |
|  | Cases of maternal anaemia | C-IPTp: 80/193  H-IPTp: 17/35 | Study data | Table 2.6B | No systematic review comparing these interventions was identified. | N/A |
|  | Cases of maternal malaria parasitaemia | C-IPTp: 102/619  H-IPTp: 3/33 | Study data | Table 2.6C | No systematic review comparing these interventions was identified. | N/A |
|  | Cases of LBW | C-IPTp: 57/950  H-IPTp: 12/146 | Study data | Table 2.6D | No systematic review comparing these interventions was identified. | N/A |

References

1. World Health Organization. WHO recommendations on antenatal care for a positive pregnancy experience: World Health Organization; 2016.

2. World Health Organization. WHO antenatal care recommendations for a positive pregnancy experience. Nutritional interventions update: multiple micronutrient supplements during pregnancy: World Health Organization; 2020.

3. Aftab N, Faraz S, Hazari K, Fahad A, AlSawalhee N, AlQedrah A, et al. Evaluation of the impact of iron deficiency anemia during pregnancy on hospital admission and utilization of hospital resources in Latifa women and children hospital, Dubai, UAE. Dubai Medical Journal. 2021;4(3):242-7.

4. Eeesha A, Yogita K, Manju T. Pharmacoeconomic Evaluation of Generic Vs Branded preparation of Ferrous Ascorbate in 14 to 24 weeks of Gestational Women. 2022;24(1):51.

5. Kashi B, M Godin C, Kurzawa ZA, Verney AMJ, Busch-Hallen JF, De-Regil LM. Multiple Micronutrient Supplements Are More Cost-effective Than Iron and Folic Acid: Modeling Results from 3 High-Burden Asian Countries. The Journal of nutrition. 2019;149(7):1222-9.

6. Kurzawa Z, Cotton CS, Mazurkewich N, Verney A, Busch-Hallen J, Kashi B. Training healthcare workers increases IFA use and adherence: Evidence and cost-effectiveness analysis from Bangladesh. Maternal & child nutrition. 2021;17(2):e13124.

7. Murugesan S, Sudakshina K, Adhimoolam M, Arthi S. Comparative study on efficacy, tolerability, and cost of different iron supplements among antenatal women with iron-deficiency anemia. National Journal of Physiology, Pharmacy and Pharmacology. 2023;13(4):802-.

8. Ray S, Neogi SB, Singh R, Devasenapathy N, Zodpey S. Is IV iron sucrose a cost-effective option for treatment of severe anaemia in pregnancy as compared with oral iron? Health Policy and Planning. 2020;35(10):1339-46.

9. Saha S, Raval D, Shah K, Saxena D. Cost-effectiveness analysis of parenteral iron therapy compared to oral iron supplements in managing iron deficiency anemia among pregnant women. Health Economics Review. 2024;14(1):3.

10. Baltussen R, Knai C, Sharan M. Iron fortification and iron supplementation are cost-effective interventions to reduce iron deficiency in four subregions of the world. J Nutr. 2004;134(10):2678-84.

11. Verney AMJ, Busch-Hallen JF, Walters DD, Rowe SN, Kurzawa ZA, Arabi M. Multiple micronutrient supplementation cost-benefit tool for informing maternal nutrition policy and investment decisions. Maternal & child nutrition. 2023(101201025):e13523.

12. Lewkowitz AK, Gupta A, Simon L, Sabol BA, Stoll C, Cooke E, et al. Intravenous compared with oral iron for the treatment of iron-deficiency anemia in pregnancy: a systematic review and meta-analysis. Journal of Perinatology. 2019;39(4):519-32.

13. Pandey AK, Gautam D, Tolani H, Neogi SB. Clinical outcome post treatment of anemia in pregnancy with intravenous versus oral iron therapy: a systematic review and meta-analysis. Scientific Reports. 2024;14(1):179.

14. Reveiz L, Gyte GM, Cuervo LG, Casasbuenas A. Treatments for iron-deficiency anaemia in pregnancy. Cochrane Database Syst Rev. 2011(10):Cd003094.

15. Athe R, Dwivedi R, Pati S, Mazumder A, Banset U. Meta-analysis approach on iron fortification and its effect on pregnancy and its outcome through randomized, controlled trials. Journal of Family Medicine and Primary Care. 2020;9(2).

16. Skolmowska D, Głąbska D, Kołota A, Guzek D. Effectiveness of Dietary Interventions in Prevention and Treatment of Iron-Deficiency Anemia in Pregnant Women: A Systematic Review of Randomized Controlled Trials. Nutrients [Internet]. 2022; 14(15).

17. Keats EC, Haider BA, Tam E, Bhutta ZA. Multiple-micronutrient supplementation for women during pregnancy. Cochrane Database Syst Rev. 2019;3(3):Cd004905.

18. King SE, Yeh PT, Rhee DK, Tuncalp Ö, Rogers LM, Narasimhan M. Self-management of iron and folic acid supplementation during pre-pregnancy, pregnancy and postnatal periods: a systematic review. BMJ Global Health. 2021;6(5):e005531.

19. Neogi SB, Devasenapathy N, Singh R, Bhushan H, Shah D, Divakar H, et al. Safety and effectiveness of intravenous iron sucrose versus standard oral iron therapy in pregnant women with moderate-to-severe anaemia in India: a multicentre, open-label, phase 3, randomised, controlled trial. Lancet Glob Health. 2019;7(12):e1706-e16.

20. Stoltzfus RJ. Iron deficiency: global prevalence and consequences. Food and nutrition bulletin. 2003;24(4_suppl2):S99-S103.

21. Haider BA, Bhutta ZA. Multiple‐micronutrient supplementation for women during pregnancy. Cochrane Database of Systematic Reviews. 2017(4).

22. Smith ER, Shankar AH, Wu LS, Aboud S, Adu-Afarwuah S, Ali H, et al. Modifiers of the effect of maternal multiple micronutrient supplementation on stillbirth, birth outcomes, and infant mortality: a meta-analysis of individual patient data from 17 randomised trials in low-income and middle-income countries. The Lancet Global Health. 2017;5(11):e1090-e100.

23. Peña‐Rosas JP, De‐Regil LM, Garcia‐Casal MN, Dowswell T. Daily oral iron supplementation during pregnancy. Cochrane database of systematic reviews. 2015(7).

24. World Health Organization. WHO guidelines for malaria. Geneva: World Health Organization; 2023 16 October 2023.

25. Choi SE, Brandeau ML, Bendavid E. Cost-effectiveness of malaria preventive treatment for HIV-infected pregnant women in sub-Saharan Africa. Malaria Journal. 2017;16(1):403.

26. Fernandes S, Sicuri E, Kayentao K, van Eijk AM, Hill J, Webster J, et al. Cost-ffectiveness of two versus three or more doses of intermittent preventive treatment for malaria during pregnancy in sub-Saharan Africa: A modelling study of meta-analysis and cost data. The Lancet Global Health. 2015;3(3):e143-e53.

27. Scott N, Delport D, Hainsworth S, Pearson R, Morgan C, Huang S, et al. Ending malnutrition in all its forms requires scaling up proven nutrition interventions and much more: a 129-country analysis. BMC medicine. 2020;18(1):356.

28. Fernandes S, Sicuri E, Halimatou D, Akazili J, Boiang K, Chandramohan D, et al. Cost effectiveness of intermittent screening followed by treatment versus intermittent preventive treatment during pregnancy in West Africa: analysis and modelling of results from a non-inferiority trial. Malaria journal. 2016;15(1):493.

29. Fernandes S, De Brouwere V, Gutman J, et al. Cost-effectiveness of intermittent preventive treatment with dihydroartemisinin-piperaquine for malaria during pregnancy: an analysis using efficacy results from Uganda and Kenya, and pooled data. The Lancet Global Health. 2020;8(12):E1512-E23.

30. Hansen KS, Ndyomugyenyi R, Magnussen P, Clarke SE. Cost-effectiveness analysis of three health interventions to prevent malaria in pregnancy in an area of low transmission in Uganda. International health. 2012;4(1):38-46.

31. Paintain L, Hill J, Ahmed R, Umbu Reku Landuwulang C, Ansariadi A, Rini Poespoprodjo J, et al. Cost-effectiveness of intermittent preventive treatment with dihydroartemisinin-piperaquine versus single screening and treatment for the control of malaria in pregnancy in Papua, Indonesia: a provider perspective analysis from a cluster-randomised trial. The Lancet Global health. 2020;8(12):e1524-e33.

32. Sicuri E, Fernandes S, Macete E, et al. Economic evaluation of an alternative drug to sulfadoxine-pyrimethamine as intermittent preventive treatment of malaria in pregnancy. PLoS ONE. 2015;10(4).

33. Cirera L, Sacoor C, Meremikwu M, Ranaivo L, Manun'Ebo MF, Pons-Duran C, et al. Cost-effectiveness of community-based distribution of intermittent preventive treatment of malaria in pregnancy in Madagascar, Mozambique, Nigeria, and the Democratic Republic of Congo. BMJ global health. 2023;8(7).

34. Mbonye AK, Hansen KS, Bygbjerg IC, Magnussen P. Intermittent preventive treatment of malaria in pregnancy: the incremental cost-effectiveness of a new delivery system in Uganda. Transactions of the Royal Society of Tropical Medicine and Hygiene. 2008;102(7):685-93.

35. Kayentao K, Garner P, Maria van Eijk A, Naidoo I, Roper C, Mulokozi A, et al. Intermittent Preventive Therapy for Malaria During Pregnancy Using 2 vs 3 or More Doses of Sulfadoxine-Pyrimethamine and Risk of Low Birth Weight in Africa: Systematic Review and Meta-analysis. JAMA. 2013;309(6):594-604.

36. Suthar AB, Vitoria MA, Nagata JM, Anglaret X, Mbori-Ngacha D, Sued O, et al. Co-trimoxazole prophylaxis in adults, including pregnant women, with HIV: a systematic review and meta-analysis. The Lancet HIV. 2015;2(4):e137-e50.

37. Gutman JR, Khairallah C, Stepniewska K, Tagbor H, Madanitsa M, Cairns M, et al. Intermittent screening and treatment with artemisinin-combination therapy versus intermittent preventive treatment with sulphadoxine-pyrimethamine for malaria in pregnancy: a systematic review and individual participant data meta-analysis of randomised clinical trials. eClinicalMedicine. 2021;41.

38. Olaleye A, Okusanya BO, Oduwole O, Esu E, Meremikwu M. A systematic review and meta-analysis of dihydroartemisinin-piperaquine versus sulphadoxine-pyrimethamine for malaria prevention in pregnancy. International Journal of Gynecology & Obstetrics. 2019;146(1):43-55.

39. Pons-Duran C, Wassenaar MJ, Yovo KE, Marín-Carballo C, Briand V, González R. Intermittent preventive treatment regimens for malaria in HIV‐positive pregnant women. Cochrane Database of Systematic Reviews. 2024(9).

40. González R, Pons‐Duran C, Piqueras M, Aponte JJ, ter Kuile FO, Menéndez C. Mefloquine for preventing malaria in pregnant women. Cochrane Database of Systematic Reviews. 2018(11).

41. Koita K, Kayentao K, Worrall E, Van Eijk AM, Hill J. Community-based strategies to increase coverage of intermittent preventive treatment of malaria in pregnancy with sulfadoxine&#x2013;pyrimethamine in sub-Saharan Africa: a systematic review, meta-analysis, meta-ethnography, and economic assessment. The Lancet Global Health. 2024;12(9):e1456-e69.

42. Gutman J, Mwandama D, Wiegand RE, Ali D, Mathanga DP, Skarbinski J. Effectiveness of intermittent preventive treatment with sulfadoxine-pyrimethamine during pregnancy on maternal and birth outcomes in Machinga district, Malawi. J Infect Dis. 2013;208(6):907-16.

43. Suthar AB, Vitoria MA, Nagata JM, Anglaret X, Mbori-Ngacha D, Sued O, et al. Co-trimoxazole prophylaxis in adults, including pregnant women, with HIV: a systematic review and meta-analysis. Lancet HIV. 2015;2(4):e137-50.

44. Klement E, Pitché P, Kendjo E, Singo A, D'Almeida S, Akouete F, et al. Effectiveness of co-trimoxazole to prevent Plasmodium falciparum malaria in HIV-positive pregnant women in sub-Saharan Africa: an open-label, randomized controlled trial. Clin Infect Dis. 2014;58(5):651-9.

45. Kapito-Tembo A, Meshnick SR, van Hensbroek MB, Phiri K, Fitzgerald M, Mwapasa V. Marked reduction in prevalence of malaria parasitemia and anemia in HIV-infected pregnant women taking cotrimoxazole with or without sulfadoxine-pyrimethamine intermittent preventive therapy during pregnancy in Malawi. J Infect Dis. 2011;203(4):464-72.

46. Mermin J, Ekwaru JP, Liechty CA, Were W, Downing R, Ransom R, et al. Effect of co-trimoxazole prophylaxis, antiretroviral therapy, and insecticide-treated bednets on the frequency of malaria in HIV-1-infected adults in Uganda: a prospective cohort study. Lancet. 2006;367(9518):1256-61.

47. Radeva-Petrova D, Kayentao K, ter Kuile FO, Sinclair D, Garner P. Drugs for preventing malaria in pregnant women in endemic areas: any drug regimen versus placebo or no treatment. Cochrane Database Syst Rev. 2014;2014(10):Cd000169.

48. Tagbor H, Cairns M, Bojang K, Coulibaly SO, Kayentao K, Williams J, et al. A Non-Inferiority, Individually Randomized Trial of Intermittent Screening and Treatment versus Intermittent Preventive Treatment in the Control of Malaria in Pregnancy. PLoS One. 2015;10(8):e0132247.

49. Kajubi R, Ochieng T, Kakuru A, Jagannathan P, Nakalembe M, Ruel T, et al. Monthly sulfadoxine&#x2013;pyrimethamine versus dihydroartemisinin&#x2013;piperaquine for intermittent preventive treatment of malaria in pregnancy: a double-blind, randomised, controlled, superiority trial. The Lancet. 2019;393(10179):1428-39.

50. Ndyomugyenyi R, Clarke SE, Hutchison CL, Hansen KS, Magnussen P. Efficacy of malaria prevention during pregnancy in an area of low and unstable transmission: an individually-randomised placebo-controlled trial using intermittent preventive treatment and insecticide-treated nets in the Kabale Highlands, southwestern Uganda. Trans R Soc Trop Med Hyg. 2011;105(11):607-16.

51. Ahmed R, Poespoprodjo JR, Syafruddin D, Khairallah C, Pace C, Lukito T, et al. Efficacy and safety of intermittent preventive treatment and intermittent screening and treatment versus single screening and treatment with dihydroartemisinin&#x2013;piperaquine for the control of malaria in pregnancy in Indonesia: a cluster-randomised, open-label, superiority trial. The Lancet Infectious Diseases. 2019;19(9):973-87.

52. González R, Mombo-Ngoma G, Ouédraogo S, Kakolwa MA, Abdulla S, Accrombessi M, et al. Intermittent Preventive Treatment of Malaria in Pregnancy with Mefloquine in HIV-Negative Women: A Multicentre Randomized Controlled Trial. PLOS Medicine. 2014;11(9):e1001733.

53. González R, Desai M, Macete E, Ouma P, Kakolwa MA, Abdulla S, et al. Intermittent Preventive Treatment of Malaria in Pregnancy with Mefloquine in HIV-Infected Women Receiving Cotrimoxazole Prophylaxis: A Multicenter Randomized Placebo-Controlled Trial. PLOS Medicine. 2014;11(9):e1001735.

54. Menendez C, Bardají A, Sigauque B, Romagosa C, Sanz S, Serra-Casas E, et al. A randomized placebo-controlled trial of intermittent preventive treatment in pregnant women in the context of insecticide treated nets delivered through the antenatal clinic. PloS one. 2008;3(4):e1934.

55. Eisele TP, Larsen DA, Anglewicz PA, Keating J, Yukich J, Bennett A, et al. Malaria prevention in pregnancy, birthweight, and neonatal mortality: a meta-analysis of 32 national cross-sectional datasets in Africa. Lancet Infect Dis. 2012;12(12):942-9.
